# Healthy Dietary Pattern Cycling Affects Gut Microbiota and Cardiovascular Disease Risk Factors: Results from a Randomized Controlled Feeding Trial with Young, Healthy Adults

**DOI:** 10.3390/nu16213619

**Published:** 2024-10-25

**Authors:** Yu Wang, Tzu-Wen L. Cross, Stephen R. Lindemann, Minghua Tang, Wayne W. Campbell

**Affiliations:** 1 Department of Nutrition Science, Purdue University, West Lafayette, IN 47907, USA; yuwang1011@outlook.com (Y.W.); tlcross@purdue.edu (T.-W.L.C.); 2 Department of Food Science, Purdue University, West Lafayette, IN 47907, USA; lindems@purdue.edu; 3 Department of Food Science and Human Nutrition, Colorado State University, Fort Collins, CO 80523, USA; minghua.tang@colostate.edu

**Keywords:** healthy eating pattern, dietary adherence, gut microbiome

## Abstract

Background: Previous research demonstrates that adopting, abandoning, and re-adopting (i.e., cycling) a healthy dietary pattern (HDP) improved, reverted, and re-improved cardiovascular disease (CVD) risk factors. In addition, changes in CVD risk factors are associated with dietary modifications of gut microbiota. Objective: We sought to assess the effects of cycling an HDP on gut microbiota and CVD risk factors. Methods: Retrospectively, we used data from a randomized controlled, crossover trial with three 3-week controlled dietary interventions, each separated by a 5-week period of participant-chosen, uncontrolled food intake. Seventeen participants (10 males, 7 females, age 26 ± 4 years old, BMI 23 ± 3 kg/m^2^) all consumed intervention diets that followed healthy U.S.-style dietary patterns. Gut microbiota composition and cardiovascular risk factors were measured before and after each HDP. Results: Repeatedly adopting and abandoning an HDP led to a cycling pattern of changes in the gut microbial community and taxonomic composition. During the HDP cycles, relative abundances of several bacterial taxa (e.g., *Collinsella, Mediterraneibacter*, *Romboutsia*, and *Dorea*) decreased and returned to baseline repeatedly. Similar HDP cycling occurred for multiple CVD risk factors (i.e., serum total cholesterol and LDL-C concentrations). Consistent negative associations were observed between changes in *Mediterraneibacter* or *Collinsella* and serum total cholesterol/HDL-C ratio. Conclusions: These results support previous findings that HDP cycling affected multiple CVD risk factors and expand the HDP cycling phenomenon to include several bacterial taxa. Young adults are encouraged to adopt and sustain a healthy dietary pattern to improve cardiovascular health, potentially through modifying gut microbiota composition.

## 1. Introduction

The 2020–2025 Dietary Guidelines for Americans (DGA) continues to recommend following a healthy dietary pattern (HDP) for health promotion and disease prevention [1]. In line with the dietary recommendation, accumulating research evidence suggests negative associations between the healthy eating index (HEI) and cardiovascular disease (CVD) risk [2,3,4]. The recommended U.S.-style HDP, which can be vegetarian or omnivorous, features the consumption of nutrient-dense, high-quality protein foods that may include lean and unprocessed meats. However, the U.S. population on average only meets about half of the dietary recommendations, as indicated by the averaged HEI scores between 50 and 60 out of 100 [5]. In the meantime, the prevalence of CVD and its risk factors continues to rise among the U.S. population, with heart disease remaining the leading cause of mortality in the country [6].

Gut microbiota respond rapidly to dietary alterations and correlate with CVD health indexes [7,8,9,10]. While considerable evidence has associated disease indexes in the host with microbial alterations, the dynamics in gut microbiota induced by external perturbations (e.g., dietary changes) over time is less understood [11]. Previous research demonstrated a cycling effect of adopting and abandoning an HDP (Mediterranean-style or Dietary Approaches to Stop Hypertension diets) on improving and reverting cardiovascular risk factors, respectively, in a similar cycling pattern [12]. In these studies, adults characterized as being of middle to old age with elevated cardiometabolic risk consumed HDPs two times in a crossover design [12]. Given the associations between changes in gut microbiota and CVD risk factors observed in the previous work [7], it is unclear whether changes in CVD risk factors induced by adopting and abandoning an HDP, independent of its type, also occur in gut microbiota. Using data collected from a previous three-arm, crossover, randomized controlled trial (RCT) in healthy young adults without diagnosed diseases [7], we aim to 1) assess the effects of cycling an HDP on gut microbiota composition (primary) and CVD risk factors (secondary), and 2) associate changes during adopting and abandoning an HDP between gut microbiota and CVD risk factors.

## 2. Materials and Methods

### 2.1. Participant Recruitment

A previous crossover study was conducted to assess the effects of having a Healthy Vegetarian Dietary Pattern (lacto-ovo), without or with the addition of unprocessed or processed lean red meat, on gut microbiota in healthy young adults [7]. Results indicated that the inclusion and exclusion of red meat in an otherwise vegetarian diet did not impact changes in the gut microbiota profile attributable to the HDP. Because there was no differential response among the three dietary interventions, we used these data to assess the HDP cycling phenomenon on a chronological basis independent of the type of dietary interventions. Individuals aged 20–35 years old, with body mass index (BMI) between 20 and 29.9 kg/m^2^ and without any diagnosed disease, were recruited between 2019 and 2020 from the surrounding area of Purdue University, West Lafayette, IN, USA. Additional details of subject recruitment and inclusion criteria are published [7]. Participants were compensated with monetary stipends at study completion. The study procedures and materials were reviewed and approved by the Purdue University Biomedical Institutional Review Board (IRB protocol # 1709019738A003). The study was registered on ClinicalTrials.gov (NCT03885544).

### 2.2. Experimental Design

**Figure 1:**
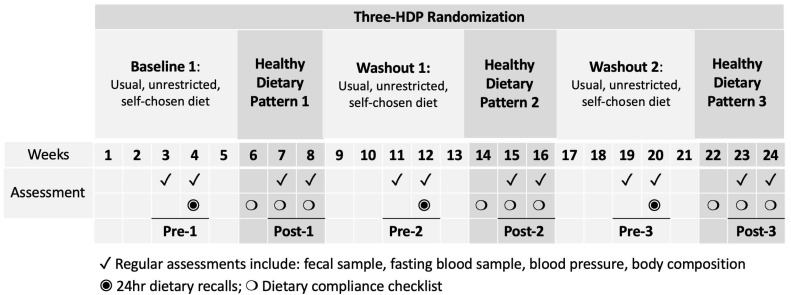
Schematic of study design and measurements. HDP, healthy dietary pattern. The HDP interventions are indicated in dark grey. The assessment timepoints before and after the three HDPs (Pre-1, Post-1, Pre-2, Post-2, Pre-3, and Post-3) are underlined for the corresponding study weeks.

Following a 5-week baseline period, participants were randomized to consume an HDP for three 3-week controlled dietary interventions, each separated by a 5-week washout (Figure 1). The weight-maintaining HDP followed a Healthy Vegetarian Dietary Pattern as recommended by the 2015–2020 DGA [1], with or without 3 ounces per day of unprocessed or processed lean red meat (beef and pork). All foods were provided to participants with menus and cooking instructions to rotate in a 4-day cycle. During baseline and washout periods, participants consumed their self-chosen, unrestricted, habitual diets. Habitual intake was assessed using 24 h dietary recalls by a research dietitian and validated using previously established protocols [13,14]. The nutrient composition was analyzed using the Nutrition Data System for Research (NDSR) Software Food and Nutrient Database (version 2013; Minneapolis, MN, USA) by a research dietitian [15] and converted into DGA food groups [1]. Self-reported dietary adherence was calculated based on completed menu booklets returned and available at the end of the study.

For each HDP, fecal and fasting blood samples were collected once per week for two consecutive weeks before starting each of the 3 HDPs (labeled “Pre”, including Pre-1, Pre-2, and Pre-3) and during the 2nd and 3rd weeks of each HDP (labeled “Post”, including Post-1, Post-2, and Post-3, Figure 1).

### 2.3. Outcome Assessments

Details of the analytical methods for gut microbiota composition and CVD risk factors were described previously [7]. Briefly, genomic DNA was extracted (FastDNA^®^ SPIN Kit and FastPrep^®^; MP Biomedicals, Irvine, CA, USA), the V4 region of the bacterial 16S rRNA gene was amplified (dual-index, primer sets 515F-806R) and sequenced (Illumina (San Diego, CA, USA) MiSeq 2 × 250), and fasting anthropometric parameters were measured, including body weight, waist-to-hip circumference ratio, and sagittal abdominal diameter; fasting cardiovascular risk factors were assessed, including serum lipids and lipoproteins, glucose, blood urea nitrogen, and creatinine (Mid America clinical Laboratories, Indianapolis, IN, USA), and blood pressures were measured in a supine position after 15 min of rest (BP785, HEM-7222-Z, Omron Healthcare, Inc., Kyoto, Japan).

### 2.4. Statistics

To assess the effects of HDP cycling on gut microbiota and CVD outcomes, we conducted statistical analyses to measure the following: (1) the effects of adopting an HDP (all Pre versus all Post); (2) the effects of adopting an HDP for the first, second, and third times (Pre-1 versus Post-1, Pre-2 versus Post-2, Pre-3 versus Post-3); (3) the effects of re-adopting an HDP (comparisons among 3 HDPs using Post, Pre, and Post–Pre change values); and (4) the effects of abandoning an HDP (Washout 1: Pre-2 versus Post-1, Washout 2: Pre-3 versus Post-2). As previously reported, the duplicate measurements from two consecutive weeks before or after treatment phases yielded comparable results [7]. The duplicate measurements were therefore pooled and treated together as Pre or Post timepoints for these analyses.

All data were tested with Shapiro–Wilk tests for normality assumptions. When normality was achieved, data were analyzed using the paired t-test or two-way repeated-measures ANOVA test (effects of time and time by HDP interaction). Box-Cox transformation was applied to achieve normality as necessary. Otherwise, data were analyzed using Kruskal–Wallis or pairwise Wilcoxon rank sum tests. Gut microbial sequencing reads were processed and analyzed using mothur (version 1.44.3) following online protocol with default settings (https://mothur.org/wiki/miseq_sop/, last access in 18 May 2022) [16]. Other statistical analyses were conducted and visualized using R (version 2022.02.0+443), SAS (version 9.4), and GraphPad Prism (version 9).

Statistical analysis plans were described previously in more detail [7]. Briefly, gut microbial composition was assessed at the community level using alpha and beta diversity measures, and at the taxonomic level using OTU- and phylotype-based approaches. Alpha diversity measures included richness indexes, Chao1 and abundance-based coverage estimator (ACE), as well as the Shannon and inverse Simpson metrics as implemented in mothur. Beta diversity matrices (computed in mothur using the “braycurtis”, “thetayc”, and “jaccard” calculators) were analyzed using the Analysis of Molecular Variance (AMOVA) and the Homogeneity of Molecular Variance (HOMOVA) to assess the separation between group clustering centroids and the difference between within-group variations, respectively. Next, the between-group difference at the taxonomic level was assessed using non-parametric linear discriminant analysis (LEfSe) and presented in LDA effect sizes [17]. Identified OTUs and genera from the LEfSe analysis were filtered to include the ones with at least 0.1% of averaged relative abundance across all samples. For bacterial taxonomic outcomes, zero-adjusted Post–Pre fold changes were used for comparisons among the 3 HDPs. For all other outcomes, Post–Pre changes were used for comparisons among the 3 HDPs. Data were presented as raw means ± standard deviations (mean ± SD) and, if applicable, as age-, sex-, and BMI-adjusted least-squares means ± standard errors (LS mean ± SE). Benjamin and Hochberg (for gut microbial outcomes) or Tukey–Kramer (for other outcomes) adjusted *p*-value < 0.05 was considered significant at the 95% confidence level.

## 3. Results

### 3.1. Participant Recruitment and Baseline Characteristics

**Figure 2:**
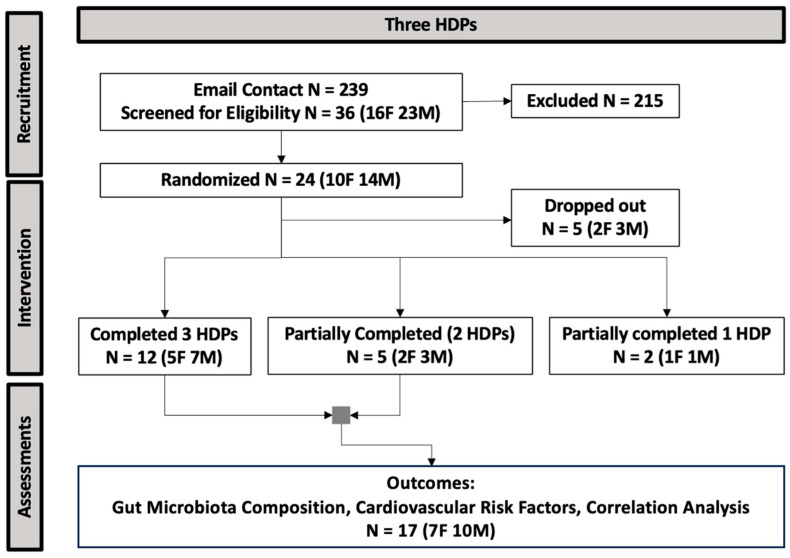
The CONSORT flow diagram of study enrollment (HDP, healthy dietary pattern; N, sample size; F, female; M, male).

The procedure of participant recruitment and enrollment is presented in Figure 2. Data from 17 participants (10 males, 7 females) who were randomized and consumed two or three HDPs were included in the analysis for fecal gut microbiota composition and CVD risk factors. Participant baseline characteristics are presented in Table 1.

### 3.2. Dietary Intakes

**Figure 3:**
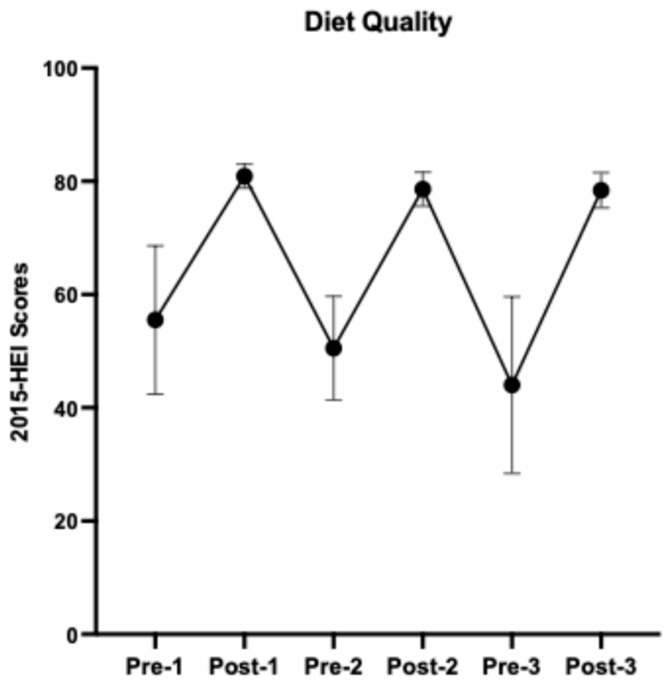
A cycling pattern of changes in diet quality from baseline 1 to healthy dietary pattern 3. Results are presented using group means and standard deviations.

Details of study menus, dietary and nutrient composition, and self-reported dietary adherence were described previously [7]. Overall, participants had an average of 95.7% dietary adherence to the prescribed HDPs. As indicated by the 2015-HEI scores (Figure 3), participants started with a relatively low diet quality (55.5 ± 13.1, mean ± SD, N = 12), similar to the U.S. averaged HEI score [1], and returned to the baseline after each HDP (washouts 1–2, 50.5 ± 9.2, N = 11; 44.0 ± 15.6, N = 7, respectively). The prescribed HDPs increased participants’ diet quality by an average of 62% (HDPs 1–3, 80.9 ± 2.1, N = 17; 78.6 ± 3.0, N = 17; 78.4 ± 3.1, N = 12, respectively). The nutrient composition and servings of DGA food groups consumed throughout the study are presented in Supplemental Tables S1 and S2, respectively.

### 3.3. Gut Microbiota Composition

Details of 16S rRNA amplicon sequencing results were described previously [7]. Of 175 fecal samples, the OTU-based approach generated 5779 OTUs. A subsampling depth at 3296 reads was used for rarefaction, which sampled 2152 OTUs for analysis at the OTU level. The phylotype-based approach identified 292 taxa for analyses across multiple taxonomic levels.

#### 3.3.1. Community-Level Characteristics

We assessed gut microbiota composition at the community level using alpha and beta diversity measures. No alpha diversity data met the normality assumption (Shapiro–Wilk tests *p* < 0.05). Adopting, abandoning, or re-adopting HDPs did not affect any of the four alpha diversity measures. Re-abandoning the HDP reduced the Chao1 and ACE indexes (Supplemental Table S3). Additionally, the Chao1 and ACE indexes were lower at Post-3 compared to Post-1 or Post-2, and the ACE index was also lower at Pre-3 compared to Pre-1 or Pre-2 (Kruskal–Wallis and pairwise Wilcoxon rank sum *p* < 0.05). Yet, the sample size for HDP 3 was smaller than the previous HDPs. Results from the first and second HDPs did not suggest a cycling pattern of changes in gut microbiota alpha diversity from cycling the HDP.

**Figure 4:**
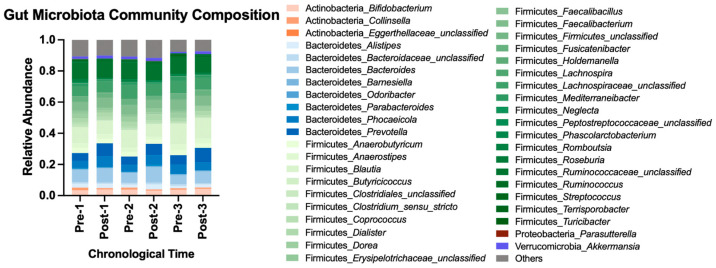
Gut microbiota community composition before and after each HDP over time. A total of 112 OTUs with at least 0.1% of abundance, representing 41 genera, are included and colored by phylum. The rest of the OTUs with less than 0.1% of relative abundance are categorized into “Others”. OTU, operational taxonomic unit.

Beta diversity metrics computed included Bray–Curtis [18], Jaccard [19], and Yue and Clayton’s theta [20]. With all data pooled for analysis, adopting the HDP shifted gut microbiota membership structure, and abandoning the HDP reverted the structure to the baseline state (AMOVA *p*-value < 0.05 using Bray–Curtis and theta metrices, Supplemental Table S4). A cycling pattern of changes in the bacterial community composition is visualized in Figure 4 using the most abundant bacterial taxa. Taken together, repeatedly adopting and abandoning the HDP affected gut microbiota community structure in a consistent cycling pattern.

#### 3.3.2. Taxonomic-Level Characteristics

Next, we assessed whether the cycling pattern of changes in gut microbiota composition also exists at the taxonomic and OTU levels. Using 2152 OTUs and 292 phylotypes generated from the Mothur pipeline, we conducted pairwise LEfSe analyses, and the results are presented in Table 2 and Table 3 and Supplemental Tables S5 and S6. The numbers of OTUs and genera that significantly differed within each HDP or washout and between HDPs at Post or at Pre are summarized in Table 2.

**Figure 5:**
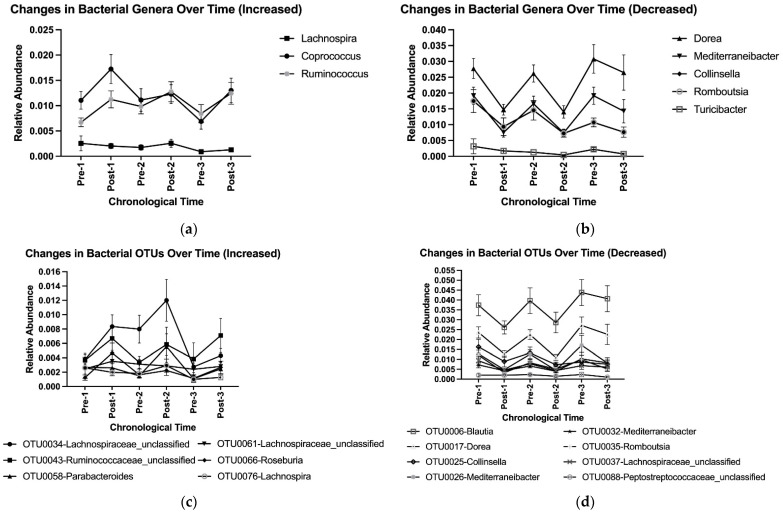
Cycling of bacterial genera that were (**a**) increased and (**b**) decreased by the prescribed healthy dietary pattern, and OTUs that were (**c**) increased and (**d**) decreased by the prescribed healthy dietary pattern, using group means and standard deviations with data pooled from the three HDPs. OTU, operational taxonomic unit.

Overall, adopting and abandoning an HDP affected microbial community composition at the genus and OTU levels (Table 3). Adoption, abandonment, re-adoption, and re-abandonment for the first time led to a cycling pattern of changes in the relative abundances of genera *Dorea*, *Mediterraneibacter*, *Collinsella*, and *Romboutsia*, and OTUs OTU0017-*Dorea*, OTU0026-*Mediterraneibacter*, and OTU0035-*Romboutsia* (Table 3, Figure 5a–d). No difference in Post–Pre fold changes in the selected genera and OTUs with at least 0.1% of relative abundance was observed among three HDPs or between two washouts (Supplemental Tables S7 and S8). Such a cycling pattern of changes was also observed at higher taxonomic levels (e.g., phyla Bacillota (previously, Firmicutes) and Bacteroidota (previously, Bacteroidetes), Figure 4, Supplemental Figure S1A–G).

### 3.4. CVD Risk Factors

**Figure 6:**
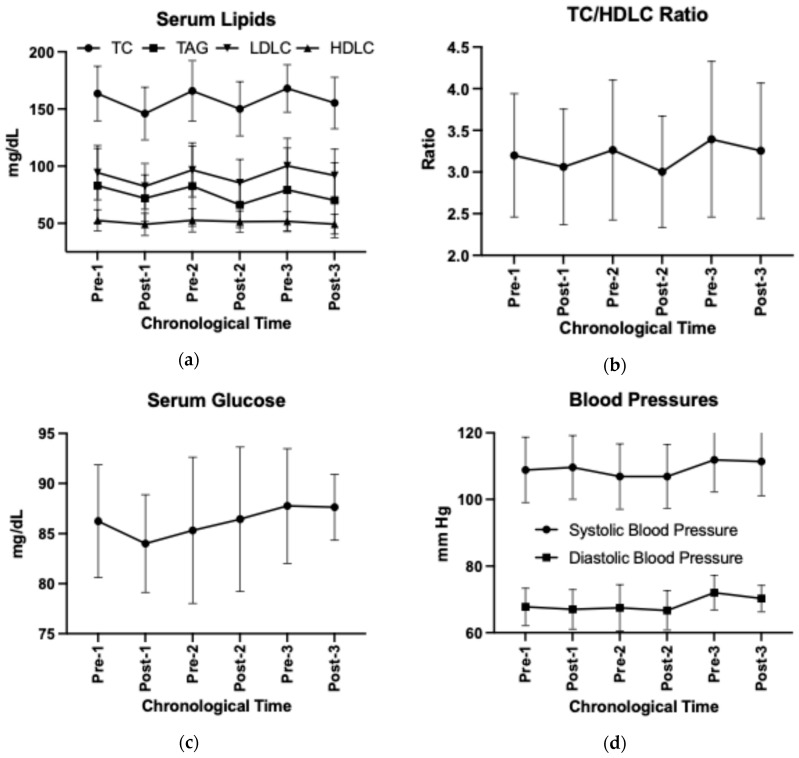
Changes in (**a**) serum lipids, (**b**) TC/HDL-C ratio, (**c**) serum glucose, and (**d**) blood pressures over time using unadjusted group means and standard deviations. TC, total cholesterol; TAG, triglycerides; HDL-C, high-density lipoprotein cholesterol; LDL-C, low-density lipoprotein cholesterol.

Adopting the HDP reduced serum total cholesterol and LDL-C concentrations (time *p* < 0.05, Supplemental Table S9), independent of the number of adoptions (time by HDP *p* > 0.05). Abandonment of HDP increased serum total cholesterol and LDL-C concentrations (Post–Pre change *p* < 0.05). The HDP cycling effect was not observed in other CVD risk factors including waist-to-hip circumference ratio, sagittal abdominal diameter, systolic or diastolic blood pressures, serum triglycerides, HDL-C, glucose, blood urea nitrogen, or creatinine concentrations (Figure 6a–d, Supplemental Table S9). The unadjusted results are visualized in Figure 6 and presented in Supplemental Table S10.

### 3.5. Individuality and Reproducibility

Notably, using data from the full completers who finished three HDPs (N = 12), we observed great individuality in gut microbial and cardiovascular responses to the HDP. The direction and magnitude of individual within-HDP Post–Pre fold changes in selected bacterial genera and OTUs appeared to be different among the three HDPs (Supplemental Figures S2B–D and S3B–D). Individual outliers existed with fold changes differing from the group averages (Supplemental Figures S2A and S3A). Similarly, individuality was observed in CVD risk factors with varying directions and magnitudes of Post–Pre changes among participants and among the three HDPs (Supplemental Figure S4A–H). Directional variability in individual responses was more frequently observed in CVD risk factors without significant changes at the group level (Supplemental Figure S4D–H) than in the ones (e.g., serum total cholesterol, LDL-C) with significant group average changes (Supplemental Figure S4A–C).

Given the variations observed in individual outcome responses, we assessed reproducibility using the selected bacterial genera and CVD risk factors. Four genera (*Dorea*, *Mediterraneibacter*, *Collinsella*, and *Romboutsia*) and two CVD outcomes (serum total cholesterol and LDL-C) were used as a cycling pattern of changes with statistical significance was observed in these outcomes. We found significant associations between HDPs at post for all four selected bacterial genera and two CVD outcomes (Supplemental Figures S5 and S6), except for the Post-HDP relative abundances for genera *Mediterraneibacter* and *Romboutsia* between HDP 3 and HDPs 1 or 2 (*p* > 0.05, Figure S5).

### 3.6. Correlations Between Changes in Bacterial Taxa and CVD Risk Factors

Lastly, we conducted correlation analyses to understand whether changes in gut microbiota were associated with changes in CVD risk factors within each HDP. The correlation coefficients (rho-values) for significant associations (*p* < 0.05) are presented in Supplemental Table S11. After adopting the HDP, changes in serum triglycerides were negatively associated with changes in OTU0025-*Collinsella*, OTU0037-*Lachnospiraceae_unclassified*, and OTU0066-*Roseburia*; changes in HDL-C were positively associated with OTU0066-*Roseburia*, *Anaerostipes*, *Collinsella*, *Romboutsia*, *Akkermansia*, and *Turicibacter*; changes in LDL-C were negatively associated with changes in OTU0008-*Fusicatenibacter*; changes in total cholesterol to HDL-C ratio were negatively associated with changes in OTU0057-*Dorea*, *Blautia*, *Mediterraneibacter*, *Collinsella*, and *Bacteroidetes_unclassified*.

After abandoning the HDP, changes in triglycerides were negatively associated with OTU0043-*Ruminococcaceae_unclassified*, and *Akkermansia*, but positively associated with *Parabacteroides*; changes in HDL-C were positively associated with *Blautia*, *Anaerostipes*, *Dorea*, *Mediterraneibacter*, *Collinsella*, *Ruminococcus*, and *Turicibacter*; changes in LDL-C were negatively associated with OTU0057-*Dorea*; changes in total cholesterol to HDL-C ratio were negatively associated with OTU0057-*Dorea*, *Blautia*, *Clostridiales_unclassified*, *Dorea*, *Mediterraneibacter*, *Collinsella*, *Akkermansia*, and *Turicibacter*. However, limited overlap in correlations was observed between the adoption and abandonment of HDP, among the three HDPs, or between the two washouts.

## 4. Discussion

Our study aimed to assess the effects of cycling an HDP on gut microbiota composition. We observed a compelling cycling pattern of changes in gut microbiota in response to repeatedly adopting and abandoning the healthy U.S.-style dietary pattern. This cycling pattern of changes exists at both community and taxonomic levels of gut microbial composition. In line with previous research that showed rapid diet-induced changes (within one day) and post-diet reversion (within three days) in gut microbial community composition (i.e., beta diversity) [10], we further demonstrated a cycling pattern of compositional changes at the genus and higher taxonomic levels. Unique to our study, re-adopting the HDP reproduced the diet-induced changes in several bacterial taxa (e.g., *Collinsella* and *Dorea*) within 3 weeks of each HDP. Our findings suggest reproducible changes in gut microbiota composition in response to HDP cycling.

Secondly, our results reproduced previous findings that adopting and abandoning an HDP two times led to a cycling pattern of improvements in CVD risk factors [12]. In contrast to previous research where CVD improvements were observed in individuals with elevated CVD risk within 5–6 weeks [12], we observed the HDP induced improvements in serum lipid profiles of healthy young adults within periods as short as 3 weeks. Furthermore, our results for serum lipids suggest a tendency of the body maintaining its ability to respond to HDP in a beneficial way for an additional third time of adopting the HDP. Particularly, previous work indicated blunted improvements in serum LDL-C (nearly halved) and weight loss after adopting HDP for the second time [12]. Yet, in our study, without changing participants’ body weights, we observed comparable improvements in serum LDL-C between adopting the HDP for the first and second times. Indirectly, our findings are in support of the potential associations between weight cycling and CVD risk [12,21].

Lastly, using exploratory post hoc analyses, we found significant associations between changes in the relative abundances of several bacterial taxa and changes in blood lipid concentrations. Some taxa demonstrated consistent associations with blood lipids during both adoption and abandonment of the HDP. For example, we found that when adopting and abandoning the HDP, changes in *Collinsella* and *Turicibacter* were positively and consistently associated with changes in serum HDL-C; changes in *Mediterraneibacter* and *Collinsella* were negatively and consistently associated with changes in TC/HDL-C ratio. These consistent associations during adoption and abandonment of HDP provide strengthened previous research on the potential involvement of gut microbiota in blood lipid metabolism [22].

Abandoning the HDP effectively returned the gut microbiota composition and CVD risk factors to baseline states within 5 weeks. Although the baseline relative abundances of a few bacteria taxa and CVD risk factors differed, the differences occurred primarily in comparison with the third HDP (HDP 3). In contrast, as indicated by the baseline correlations among HDPs, abandoning the HDP failed to reproducibly return the selected bacterial genera (e.g., *Collinsella* and *Dorea*) to the same baseline relative abundances when participants consumed self-chosen, habitual diets. However, repeatedly adopting the HDP effectively and reproducibly affected the selected genera leading to significant associations between HDPs at the post timepoint. Our findings emphasize the importance of controlling baseline dietary intake for repeated measures, as well as long-term adherence to dietary interventions to maintain and stabilize gut microbial composition for CVD benefits.

Gut microbial research in humans frequently identifies great variations within and among individuals in gut microbial profiles and responses to dietary interventions [23]. Yet, increasing evidence suggests environmental factors such as diet can predominantly shape the gut microbial composition despite the differences in highly individualized intrinsic factors [24]. Consistent with this body of literature, we showed that adopting and abandoning the HDP effectively shifted gut microbiota structure regardless of the individual differences in responses. Our previous finding suggested that diet-induced changes in two genera, *Collinsella* and *Mediterraneibacter*, were not affected by the type of study diets consumed by our participants (that is, with or without unprocessed or processed lean red meat) [7]. Although we observed some variations in the directions and magnitudes of changes among participants, on a group basis, responses of these two genera were comparable among the three HDPs. The significant correlations among HDPs at Post (after HDP) phases further demonstrate the reproducible effects of repeated adoption of HDP on gut microbiota.

Supporting and expanding upon our prior research with adults with elevated cardiometabolic risk [12], this study demonstrates the effects of repeatedly consuming (cycling) an HDP at the whole-food level on gut microbial composition with CVD associations in healthy human adults. Our study is novel in that we not only reproduced the HDP-induced CVD improvements among young adults with generally healthy metabolic profiles and stable body weights within a shorter duration, but also extended the findings to an additional cycle of HDP with reproducible gut microbial and CVD responses. We are mindful that the smaller sample size in our third HDP cycle reduced the statistical power for detecting significant changes in CVD risk factors. Although we did not have statistical significance to support the change in HDP 3, we observed a trend of cycling in blood lipids after adopting the HDP for the third time. Future research should explore the HDP-induced changes in gut microbiota at the species level for potential effects on CVD risk, as well as functional and therapeutic applications. Interestingly, weight cycling (i.e., yoyo dieting) was found to be associated with increased cardiometabolic risk [21]. Future study should consider including a control group that consumes participants’ usual self-chosen, unrestricted diet throughout the period of study as a negative control to explore how the pattern cycling compares to maintaining an unhealthy dietary pattern, especially in the long term.

## 5. Conclusions

Our results show that cyclically adopting and abandoning a healthy U.S.-style dietary pattern repeatedly changes gut microbial composition and improves, then worsens, cardiovascular risk factors. Changes in bacterial relative abundances were consistently associated with changes in blood lipids and lipoproteins. Health professionals and researchers should strategize the promotion of long-term adherence to healthy dietary patterns for sustainable cardiovascular health improvements, potentially through modifications of gut microbial composition. Repeated attempts to adopt a healthy dietary pattern are encouraged for individuals whose first attempts were not successful or sustained.

## Figures and Tables

**Table 1:** Participant baseline characteristics ^1^.

Outcomes	
**Anthropometrics**	
Sample size N (F/M)	N = 17 (7/10)
Age (y)	26 ± 4
Weight (kg)	69 ± 15
BMI (kg/m^2^)	23 ± 3
Waist-to-hip circumference (cm)	0.87 ± 0.05
Sagittal abdominal diameter (cm)	19.0 ± 2.4
Systolic blood pressure (mm Hg)	109 ± 9.8
Diastolic blood pressure (mm Hg)	68 ± 5.6
**Serum biomarkers**	
Total cholesterol (mg/dL)	164 ± 23.9
Triglycerides (mg/dL)	83 ± 32.5
HDL cholesterol (mg/dL)	53 ± 9.2
LDL cholesterol (mg/dL)	94 ± 23.8
Cholesterol/HDL-C ratio	3.2 ± 0.7
Glucose (mg/dL)	86.2 ± 5.6
Blood Urea Nitrogen (mg/dL)	13.1 ± 2.8
Creatinine (mg/dL)	0.9 ± 0.1

^1^ Results are presented as mean ± standard deviation.

**Table 2:** Numbers of genera and OTUs *.

Level	OTUs			Genera		
Comparisons	Mothur Output	LEfSe Output	RA > 0.1%	Mothur Output	LEfSe Output	RA > 0.1%
HDP 1, Post-1 vs. Pre-1	Total: 292 RA > 0.1%: 49	6	5	Total: 2152 RA > 0.1%: 90	10	8
Washout 1, Pre-2 vs. Post-1		9	4		7	6
HDP 2, Post-2 vs. Pre-2		7	4		5	4
Washout 2, Pre-3 vs. Post-2		25	13		17	10
HDP 3, Post-3 vs. Pre-3		6	3		1	1
All HDP, Post vs. Pre		17	8		18	14
All Washouts, Post vs. Pre		22	10		21	15
Post, Post-1 vs. Post 2		2	1		0	0
Post, Post-1 vs. Post-3		4	2		4	3
Post, Post-2 vs. Post-3		5	3		5	4
Pre, Pre-1 vs. Pre-2		3	1		2	0
Pre, Pre-1 vs. Pre-3		3	2		6	3
Pre, Pre-2 vs. Pre-3		3	1		4	2

* OTU, operational taxonomic unit; LEfSe, non-parametric linear discriminant analysis; RA, relative abundance. HDPs 1–3 are the chronological order in which participants consumed the healthy dietary patterns (HDPs). The Post vs. Pre for all HDPs represents the comparison of Post-HDP vs. Pre-HDP samples with data from three HDPs pooled for analysis; the Post vs. Pre for all Washouts represents the comparison of pre-next HDP vs. post-previous HDP with data from 2 washouts pooled for analysis.

**Table 3:** Bacterial genera and OTUs with significant changes or differences *.

Comparisons	Increased	Decreased
HDP 1, Post-1 vs. Pre-1	Genus: None OTUs: OTU0066-*Roseburia* OTU0071-*Ruminococcaceae_unclassified*	Genera: *Dorea*, *Mediterraneibacter*, *Collinsella*, *Romboutsia*, *Lachnospira* OTUs: OTU0017-*Dorea* OTU0025-*Collinsella* OTU0026-*Mediterraneibacter* OTU0035-*Romboutsia* OTU0057-*Dorea* OTU0076-*Lachnospira*
Washout 1, Pre-2 vs. Post-1	Genera: *Dorea*, *Mediterraneibacter*, *Collinsella*, *Romboutsia* OTUs: OTU0017-*Dorea* OTU0026-*Mediterraneibacter* OTU0032-*Mediterraneibacter* OTU0035-*Romboutsia* OTU0056-*Roseburia* OTU0057-*Dorea*	None
HDP 2, Post-2 vs. Pre-2	Genera: None OTUs: None	Genera: *Anaerostipes*, *Dorea*, *Mediterraneibacter*, *Romboutsia, Collinsella* OTUs: OTU0017-*Dorea* OTU0026-*Mediterraneibacter* OTU0032-*Mediterraneibacter* OTU0035-*Romboutsia*
Washout 2, Pre-3 vs. Post-2	Genera: *Blautia*, *Dorea*, *Mediterraneibacter*, *Streptococcus*, *Collinsella*, *Romboutsia*, *Faecalibacillus*, *Turicibacter* OTUs: OTU0006-*Blautia* OTU0017-*Dorea* OTU0026-*Mediterraneibacter* OTU0035-*Romboutsia* OTU0038-*Faecalibacillus* OTU0110-*Turicibacter*	Genera: *Clostridiales_unclassified*, *Parabacteroides*, *Bacteria_unclassified*, *Lachnospira*, *Bacteroidetes_unclassified* OTUs: OTU0034-*Lachnospiraceae_unclassified* OTU0044-*Ruminococcaceae_unclassified* OTU0058-*Parabacteroides* OTU0076-*Lachnospira*
HDP 3, Post-3 vs. Pre-3	Genus: *Lachnospira* OTUs: None	Genera: *Collinsella*, *Turicibacter* OTUs: OTU0026-*Mediterraneibacter*
All HDPs, Post vs. Pre	Genera: *Coprococcus*, *Ruminococcus*, *Lachnospira* OTUs: OTU0034-*Lachnospiraceae_unclassified* OTU0043-*Ruminococcaceae_unclassified* OTU0058-*Parabacteroides* OTU0061-*Lachnospiraceae_unclassified* OTU0066-*Roseburia* OTU0076-*Lachnospira*	Genera: *Dorea*, *Mediterraneibacter*, *Collinsella*, *Romboutsia*, *Turicibacter* OTUs: OTU0006-*Blautia* OTU0017-*Dorea* OTU0025-*Collinsella* OTU0026-*Mediterraneibacter* OTU0032-*Mediterraneibacter* OTU0035-*Romboutsia* OTU0037-*Lachnospiraceae_unclassified* OTU0088-*Peptostreptococcaceae_unclassified*
All Washouts, Post vs. Pre	Genera: *Blautia*, *Dorea*, *Mediterraneibacter*, *Collinsella*, *Romboutsia*, *Turicibacter* OTUs: OTU0008-*Fusicatenibacter* OTU0017-*Dorea* OTU0026-*Mediterraneibacter* OTU0032-*Mediterraneibacter* OTU0035-*Romboutsia* OTU0056-*Roseburia* OTU0057-*Dorea* OTU0110-*Turicibacter* OTU0006-*Blautia*	Genera: *Parabacteroides*, *Coprococcus*, *Akkermansia*, *Lachnospira* OTUs: OTU0034-*Lachnospiraceae_unclassified* OTU0039-*Alistipes* OTU0043-*Ruminococcaceae_unclassified* OTU0058-*Parabacteroides* OTU0066-*Roseburia* OTU0076-*Lachnospira*
At Post, Among HDPs (Post-1 vs. Post-2 vs. Post-3)	Genera: *Anaerostipes* (higher in Post-1 than Post-2) *Parabacteroides*, *Clostridium_IV* (higher in Post-1 than Post-3) *Dorea* (higher in Post-3 than Post-2) *Parabacteroides*, *Bacteria_unclassified* (higher in Post-2 than Post-3) OTUs: OTU0050-*Blautia*, OTU0057-*Dorea* (higher in Post-3 than Post-1) OTU0063-*Parabacteroides* (higher in Post-1 than Post-3) OTU0017-*Dorea*, OTU0057-*Dorea* (higher in Post-3 than Post-2) OTU0034-*Lachnospiraceae_unclassified*, OTU0063-*Parabacteroides* (higher in Post-2 than Post-3)	
At Pre, Among HDPs (Pre-1 vs. Pre-2 vs. Pre-3)	Genera: *Roseburia* (higher in Pre-2 than Pre-1) *Clostridiales_unclassified*, *Bacteria_unclassified* (higher in Pre-1 than Pre-3) *Streptococcus* (higher in Pre-3 than Pre-2) OTUs: OTU0023-*Blautia*, OTU0050-*Blautia*, OTU0073-*Lachnospiraceae_unclassified* (higher in Pre-3 than Pre-1) OTU0012-*Bacteroides*, OTU0078-*Faecalibacterium* (higher in Pre-2 than Pre-3)	

* OTU, operational taxonomic unit. HDPs 1–3 is the chronological order in which participants consumed the healthy dietary patterns (HDPs). The Post vs. Pre for All HDPs represents the comparison of post-HDP vs. pre-HDP samples with data from three HDPs pooled for analysis; the Post vs. Pre for All Washouts represents the comparison of pre-next HDP vs. post-previous HDP with data from 2 washouts pooled for analysis.

## Data Availability

The raw data (deidentified) supporting the conclusions of this article will be made available by the authors on request.

## Supplementary Materials

The following supporting information can be downloaded at: https://www.mdpi.com/article/10.3390/nu16213619/s1, Figure S1: Taxa that were differentially represented based on linear discriminant analyses between post versus pre samples; Figure S2. Heatmaps of log2-delta values (post-/pre-fold changes) of selected genera with at least 0.1% of relative abundance with three HDPs combined (A), within HDP 1 (B), HDP 2 (C), and HDP 3 (D); Figure S3. Heatmaps of log2-delta values (post-pre fold changes) of selected OTUs with at least 0.1% of relative abundance with three HDPs combined (A), within HDP 1 (B), HDP 2 (C), and HDP 3 (D); Figure S4. Individual responses of serum total cholesterol (A), LDL-C (B), HDL-C (C), Triglycerides (D), TC/HDLC ratio (E), glucose (F), and systolic blood pressure (G) and diastolic blood pressure (H); Figure S5. Pairwise correlation analyses of selected bacterial genera using post-HDP values among the 3 HDPs; Figure S6. Pairwise correlation analyses of selected bacterial genera using pre-HDP values among the 3 HDPs; Figure S7. Pairwise correlation analyses of selected blood lipids using post-HDP values among the 3 HDPs; Figure S8. Pairwise correlation analyses of selected blood lipids using pre-HDP values among the 3 HDPs; Figure S9. Correlation analyses of selected bacterial taxa and blood lipids between pre-1 and post-1. Table S1. Nutrients of study diets consumed during each HDP and habitual intakes at baseline and washouts; Table S2. Daily food group servings from baseline, each HDP (averages of 4-day menus), and washouts, presented in cup or ounce-equivalents; Table S3. Alpha diversity assessments; Table S4. Beta diversity assessments; Table S5. Taxa that significantly differed between groups based on LEfSe analyses; Table S6. OTUs that significantly differed between groups based on LEfSe analyses; Table S7. Comparison of post/pre fold changes of selected taxa with at least 0.1% of relative abundance among HDPs 1-3 and between washouts 1-2; Table S8. Comparison of post/pre fold changes of selected OTUs with at least 0.1% of relative abundance among HDPs 1-3 and between washouts 1-2; Table S9. Participant responses in cardiovascular risk factors from consuming study diets for 3 times (HDPs 1-3). Results are presented as LS means ± SEM; Table S10. Raw (unadjusted) means and SD of participant responses in cardiovascular risk factors from consuming study diets for 3 times (HDPs 1-3); Table S11. Rho values of significant correlations between changes in blood lipids and bacterial taxa.
