# Supplementary material for: Healthy Dietary Pattern Cycling Affects Gut Microbiota and Cardiovascular Disease Risk Factors: Results from a Randomized Controlled Feeding Trial with Young, Healthy Adults
Source: Nutrients. 2024 Oct 25;16(21):3619. doi: 10.3390/nu16213619 (PMC11547453; doi:10.3390/nu16213619)
Supplement: Supplementary file 1 [file nutrients-16-03619-s001.zip › Supplemental Figures.pdf]

Healthy Dietary Pattern Cycling Affects Gut Microbiota and Cardiovascular Disease Risk Factors: Results from a Randomized Controlled Feeding Trial with Young, Healthy Adults

List of Supplemental Figures

Supplemental Figure S1. Taxa that were differentially represented based on linear discriminant analyses between post versus pre samples[1]. 2
Supplemental Figure S2. Heatmaps of log2-delta values (post-/pre-fold changes) of selected genera with at least 0.1% of relative abundance with three HDPs combined (A), within HDP 1 (B), HDP 2 (C), and HDP 3 (D)[1]. ... 5
Supplemental Figure S3. Heatmaps of log2-delta values (post-pre fold changes) of selected OTUs with at least 0.1% of relative abundance with three HDPs combined (A), within HDP 1 (B), HDP 2 (C), and HDP 3 (D)[1]. ... 6
Supplemental Figure S4. Individual responses of serum total cholesterol (A), LDL-C (B), HDL-C (C), Triglycerides (D), TC/HDLC ratio (E), glucose (F), and systolic blood pressure (G) and diastolic blood pressure (H)[1]........ 7
Supplemental Figure S5. Pairwise correlation analyses of selected bacterial genera using post-HDP values among the 3 HDPs[1]. 8
Supplemental Figure S6. Pairwise correlation analyses of selected bacterial genera using pre-HDP values among the 3 HDPs[1]. 9
Supplemental Figure S7. Pairwise correlation analyses of selected blood lipids using post-HDP values among the 3 HDPs[1]........ 10
Supplemental Figure S8. Pairwise correlation analyses of selected blood lipids using pre-HDP values among the 3 HDPs[1]........ 11
Supplemental Figure S9. Correlation analyses of selected bacterial taxa and blood lipids between pre-1 and post-1[1]. 12

Supplemental Figure S1. Taxa that were differentially represented based on linear discriminant analyses between post versus pre samples[1].

[1](A) when adopting the healthy dietary pattern (HDP), (B) when abandoning the HDP, (C) when adopting the HDP for the first time (Trial 1), (D) when abandoning the HDP for the first time (post Trial 1 vs pre Trial 2), (E) when adopting the HDP for the second time (Trial 2), (F) when abandoning the HDP for the second time (post Trial 2 vs pre Trial 3), and (G) when adopting the HDP for the third time (Trial 3). Bacterial taxa were represented with abbreviated taxonomic level followed by taxonomic name: p, phylum; c, class; o, order; f, family; g, genus.

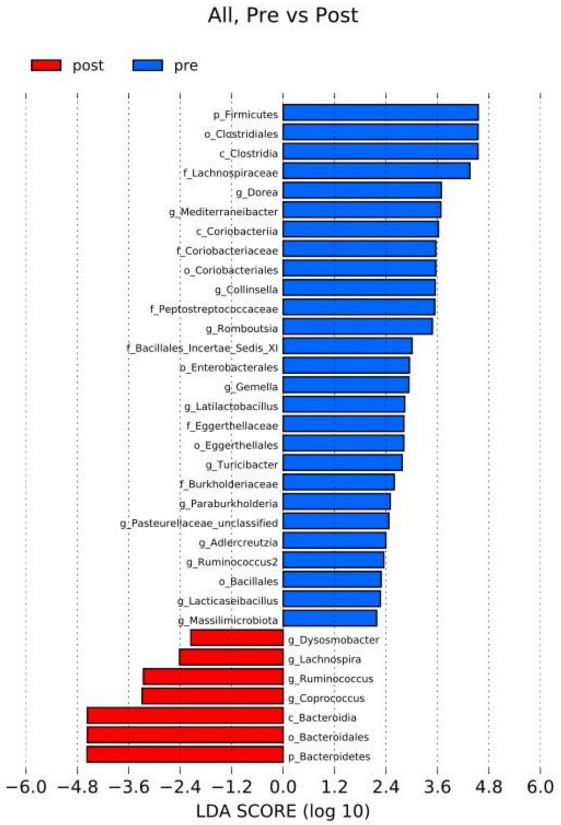

A

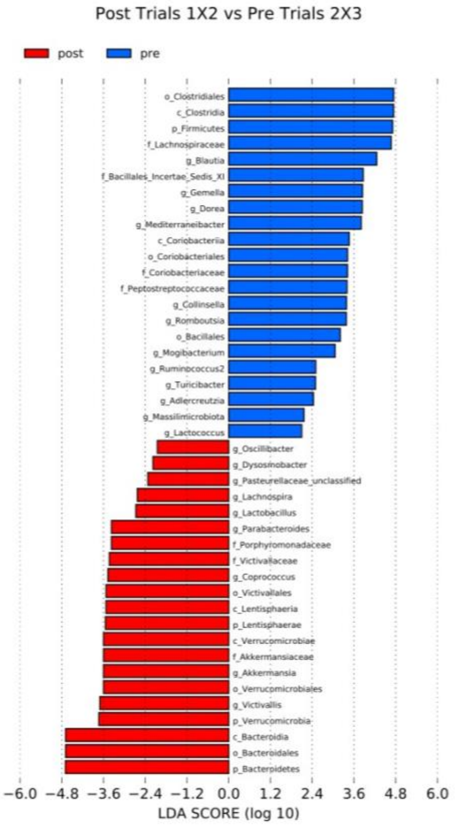

B

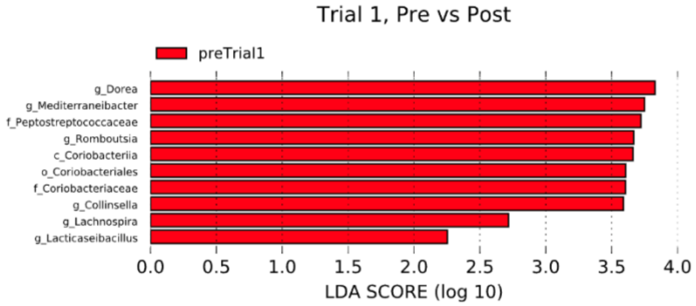
Trial 1, Pre vs Post
preTrial1
g_Dorea
g_Mediterraneibacter
f_Peptostreptococcaceae
g_Romboutsia
c_Coriobacteriia
o_Coriobacteriales
f_Coriobacteriaceae
g_Collinsella
g_Lachnospira
g_Lacticaseibacillus
0.0 0.5 1.0 1.5 2.0 2.5 3.0 3.5 4.0
LDA SCORE (log 10)

C

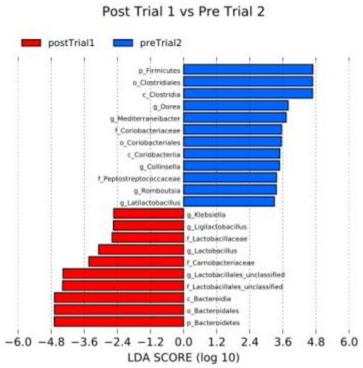
Post Trial 1 vs Pre Trial 2
postTrial1
preTrial2
p_Firmicutes
o_Clostridiales
c_Clostridia
g_Dorea
g_Mediterraneibacter
f_Coriobacteriaceae
o_Coriobacteriales
c_Coriobacteriia
g_Collinsella
f_Peptostreptococcaceae
g_Romboutsia
g_Latilactobacillus
g_Klebsiella
g_Ligilactobacillus
f_Lactobacillaceae
g_Lactobacillus
f_Carnobacteriaceae
g_Lactobacillales_unclassified
f_Lactobacillales_unclassified
c_Bacteroidia
o_Bacteroidales
p_Bacteroidetes
−6.0 −4.8 −3.6 −2.4 −1.2 0.0 1.2 2.4 3.6 4.8 6.0
LDA SCORE (log 10)

D

E

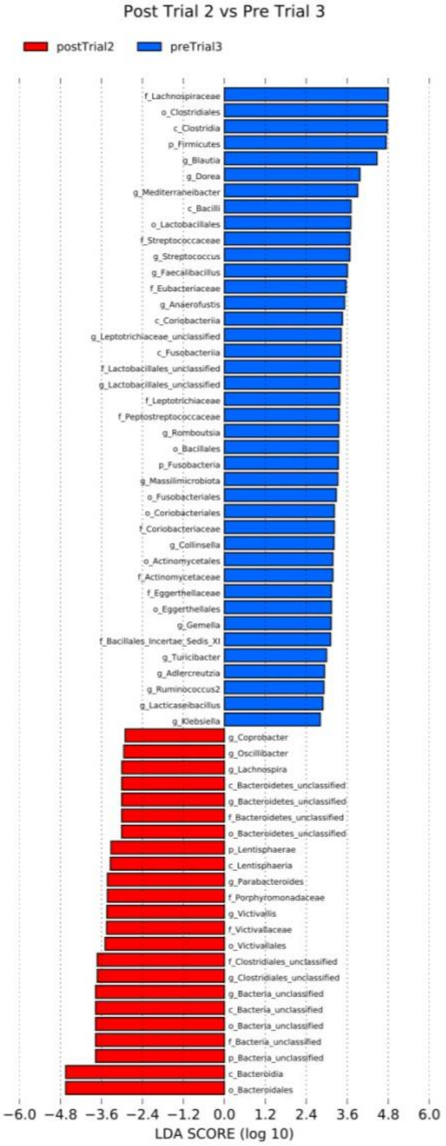
Post Trial 2 vs Pre Trial 3
postTrial2
preTrial3
f_Lachnospiraceae
o_Clostridiales
c_Clostridia
p_Firmicutes
g_Blautia
g_Dorea
g_Mediterraneibacter
c_Bacilli
o_Lactobacillales
f_Streptococcaceae
g_Streptococcus
g_Faecalibacillus
f_Eubacteriaceae
g_Anaerofustis
c_Coriobacteriia
g_Leptotrichiaceae_unclassified
c_Fusobacteriia
f_Lactobacillales_unclassified
g_Lactobacillales_unclassified
f_Leptotrichiaceae
f_Peptostreptococcaceae
g_Romboutsia
o_Bacillales
p_Fusobacteria
g_Massilimicrobiota
o_Fusobacteriales
o_Coriobacteriales
f_Coriobacteriaceae
g_Collinsella
o_Actinomycetales
f_Actinomycetaceae
f_Eggerthellaceae
o_Eggerthellales
g_Gemella
f_Bacillales_Incertae_Sedis_XI
g_Turicibacter
g_Adlercreutzia
g_Ruminococcus2
g_Lacticaseibacillus
g_Klebsiella
g_Coprobacter
g_Oscillibacter
g_Lachnospira
c_Bacteroidetes_unclassified
g_Bacteroidetes_unclassified
f_Bacteroidetes_unclassified
o_Bacteroidetes_unclassified
p_Lentisphaerae
c_Lentisphaeria
g_Parabacteroides
f_Porphyromonadaceae
g_Victivallis
f_Victivallaceae
o_Victivallales
f_Clostridiales_unclassified
g_Clostridiales_unclassified
g_Bacteria_unclassified
c_Bacteria_unclassified
o_Bacteria_unclassified
f_Bacteria_unclassified
p_Bacteria_unclassified
c_Bacteroidia
o_Bacteroidales
−6.0 −4.8 −3.6 −2.4 −1.2 0.0 1.2 2.4 3.6 4.8 6.0
LDA SCORE (log 10)

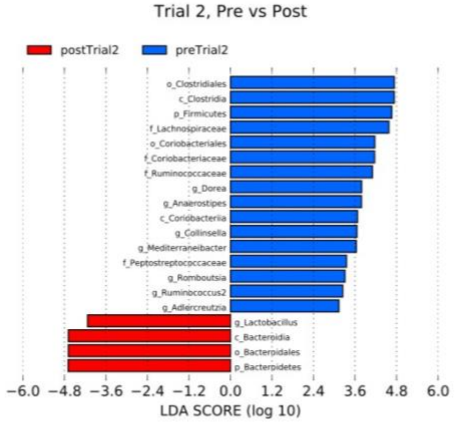
Trial 2, Pre vs Post
postTrial2
preTrial2
o_Clostridiales
c_Clostridia
p_Firmicutes
f_Lachnospiraceae
o_Coriobacteriales
f_Coriobacteriaceae
f_Ruminococcaceae
g_Dorea
g_Anaerostipes
c_Coriobacteriia
g_Collinsella
g_Mediterraneibacter
f_Peptostreptococcaceae
g_Romboutsia
g_Ruminococcus2
g_Adlercreutzia
g_Lactobacillus
c_Bacteroidia
o_Bacteroidales
p_Bacteroidetes
−6.0 −4.8 −3.6 −2.4 −1.2 0.0 1.2 2.4 3.6 4.8 6.0
LDA SCORE (log 10)

F

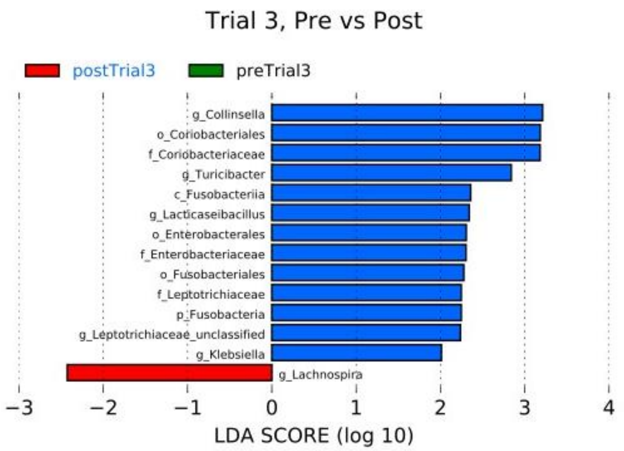
Trial 3, Pre vs Post
postTrial3
preTrial3
g_Collinsella
o_Coriobacteriales
f_Coriobacteriaceae
g_Turicibacter
c_Fusobacteriia
g_Lacticaseibacillus
o_Enterobacterales
f_Enterobacteriaceae
o_Fusobacteriales
f_Leptotrichiaceae
p_Fusobacteria
g_Leptotrichiaceae_unclassified
g_Klebsiella
g_Lachnospira
−3
−2
−1
0
1
2
3
4
LDA SCORE (log 10)

G

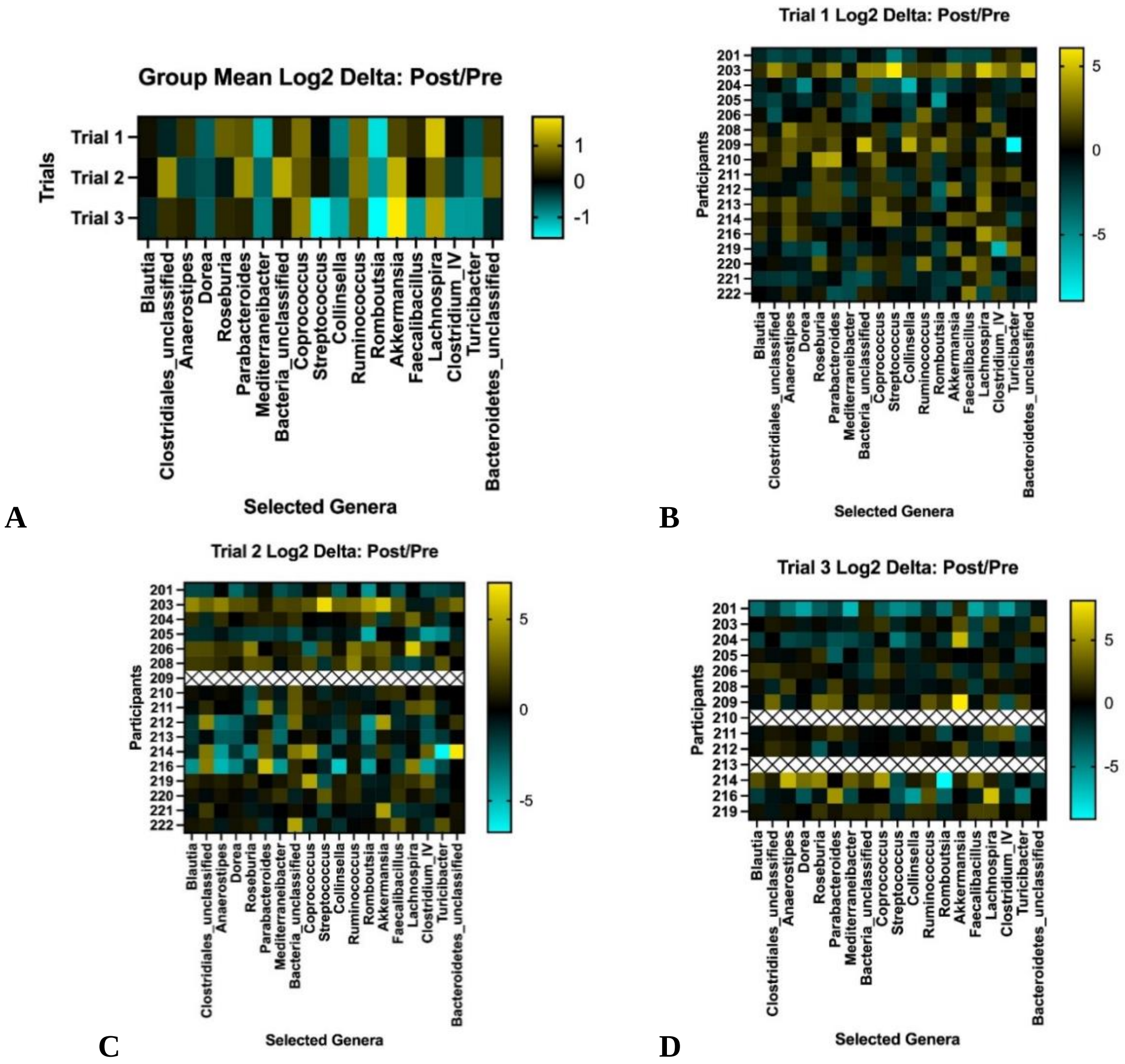

Supplemental Figure S2. Heatmaps of log2-delta values (post-/pre-fold changes) of selected genera with at least 0.1% of relative abundance with three HDPs combined (A), within HDP 1 (B), HDP 2 (C), and HDP 3 (D)[1].

[1]HDP, healthy dietary pattern, or trial.

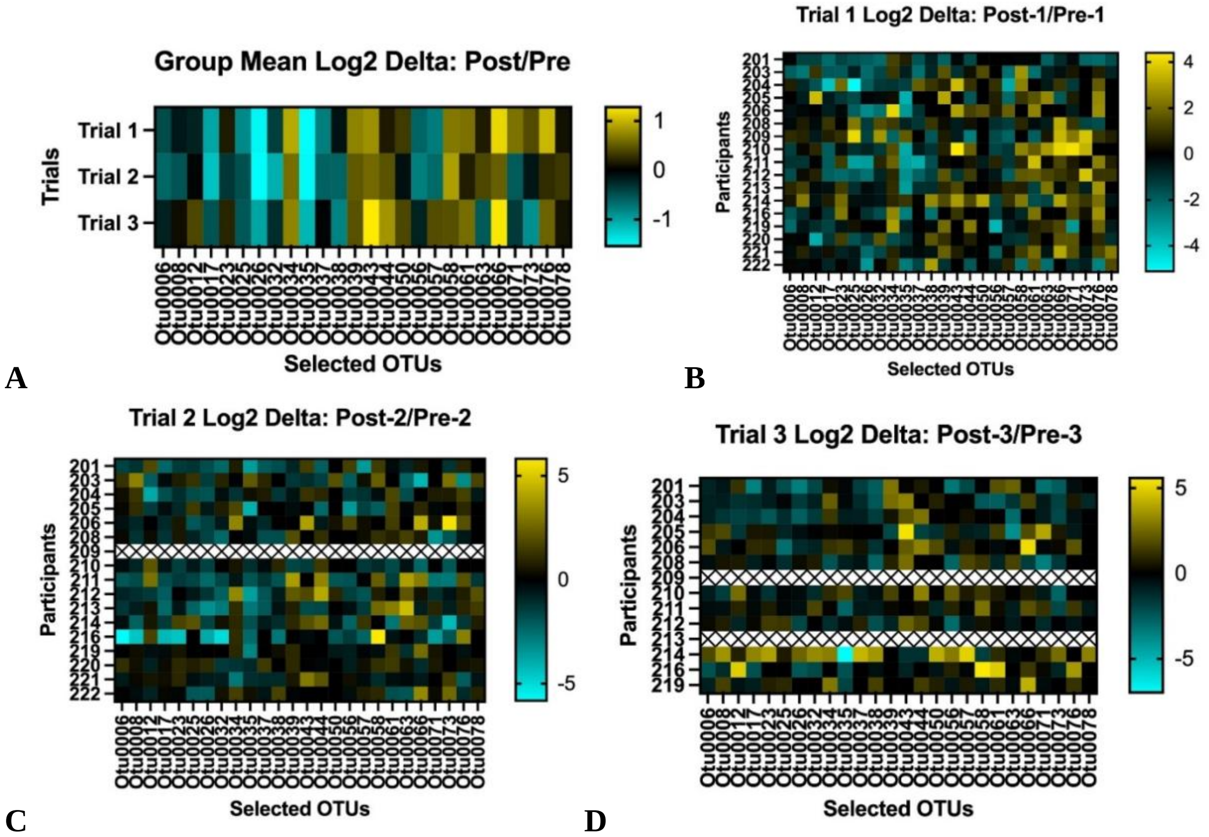

Supplemental Figure S3. Heatmaps of log2-delta values (post-pre fold changes) of selected OTUs with at least 0.1% of relative abundance with three HDPs combined (A), within HDP 1 (B), HDP 2 (C), and HDP 3 (D)[1].

[1]HDP, healthy dietary pattern, or trial.

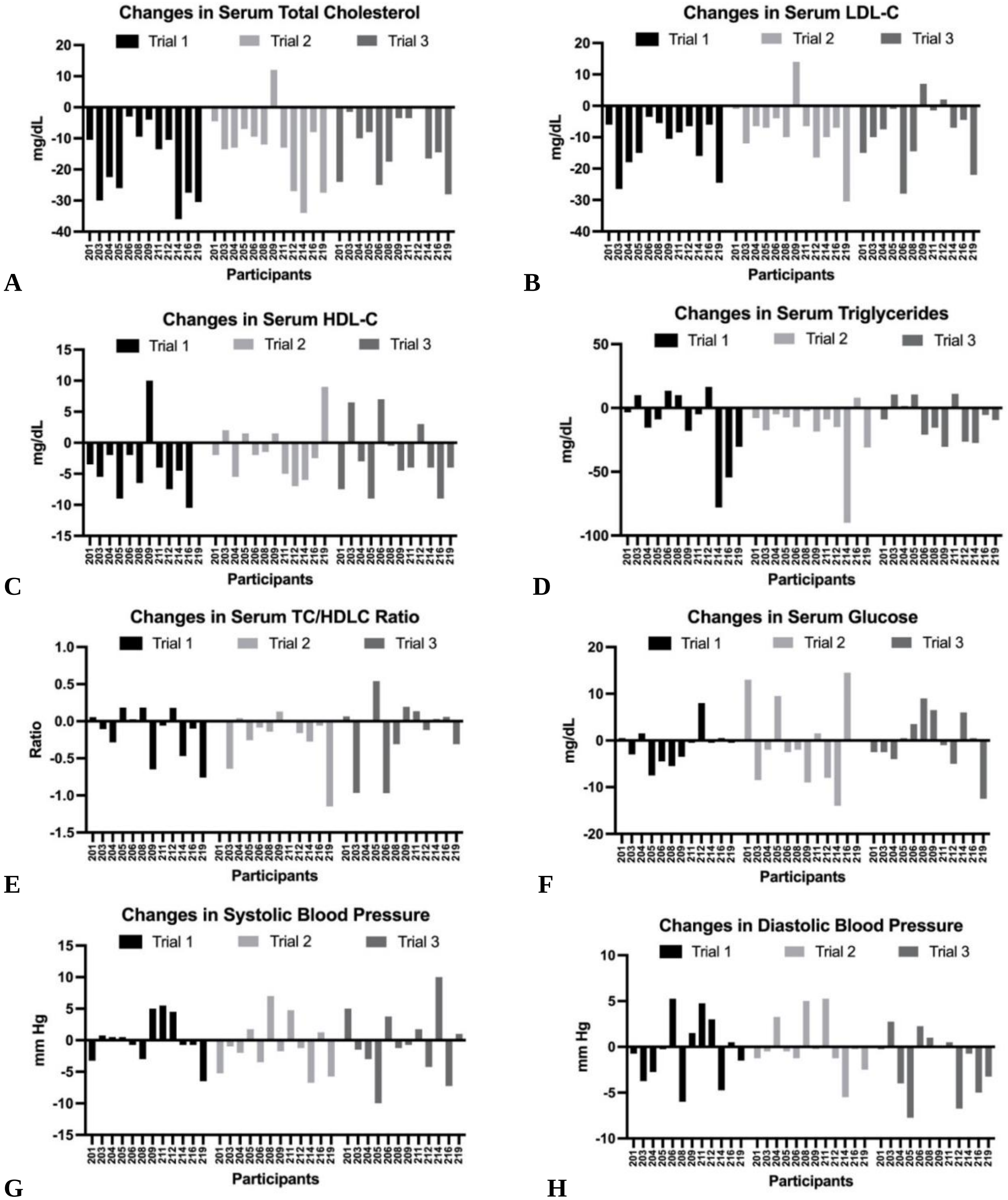

Supplemental Figure S4. Individual responses of serum total cholesterol (A), LDL-C (B), HDL-C (C), Triglycerides (D), TC/HDLC ratio (E), glucose (F), and systolic blood pressure (G) and diastolic blood pressure (H)[1].

[1]Using unadjusted post-pre change values (N=12). LDL-C, low-density lipoprotein cholesterol; TC, total cholesterol; HDL-C, high-density lipoprotein cholesterol. Trial refers to healthy dietary pattern (HDP).

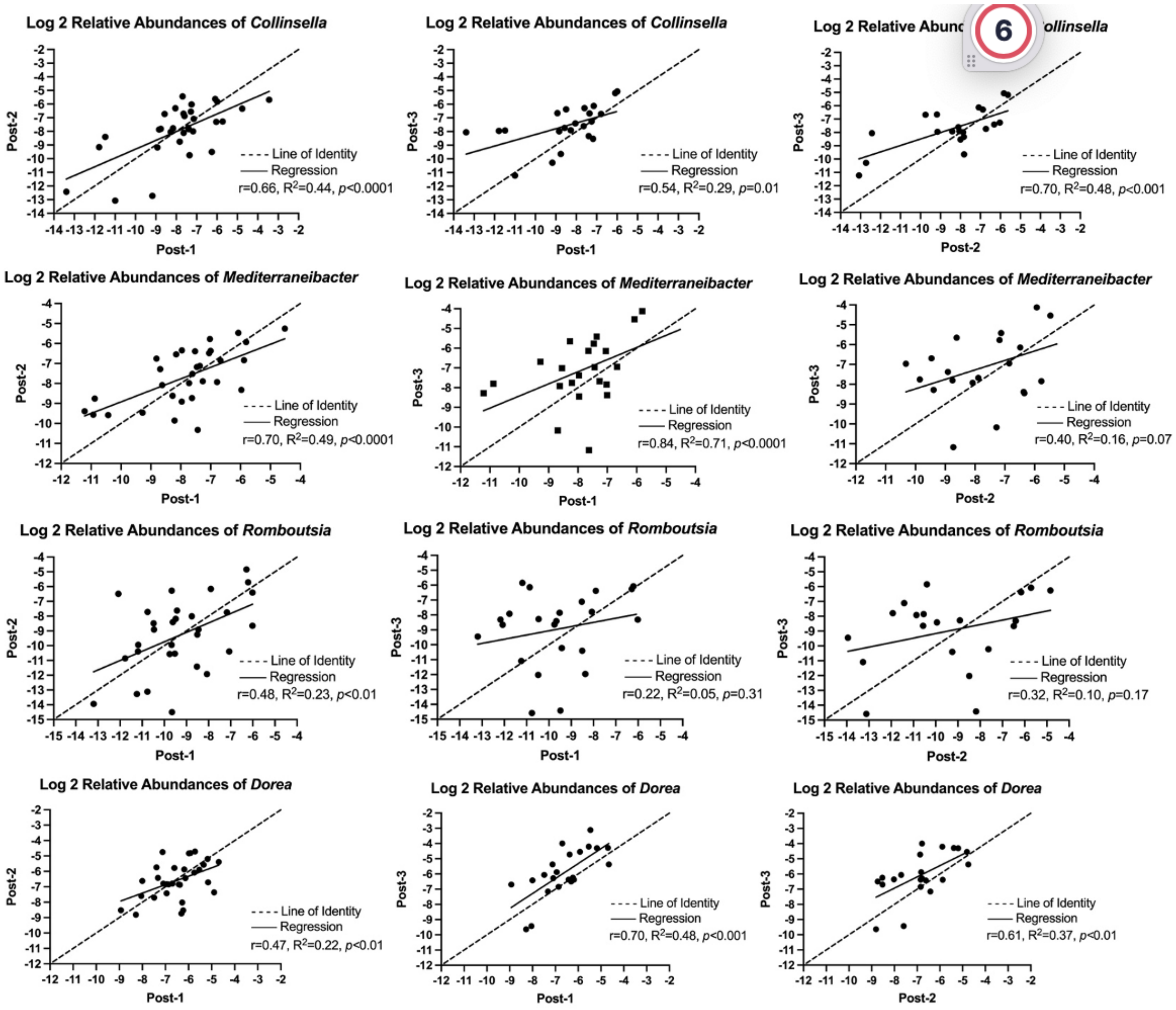

Supplemental Figure S5. Pairwise correlation analyses of selected bacterial genera using post-HDP values among the 3 HDPs[1].

[1]Pearson's r and R^2 with significance measurement (p<0.05) are included in each plot. HDP, healthy dietary pattern.

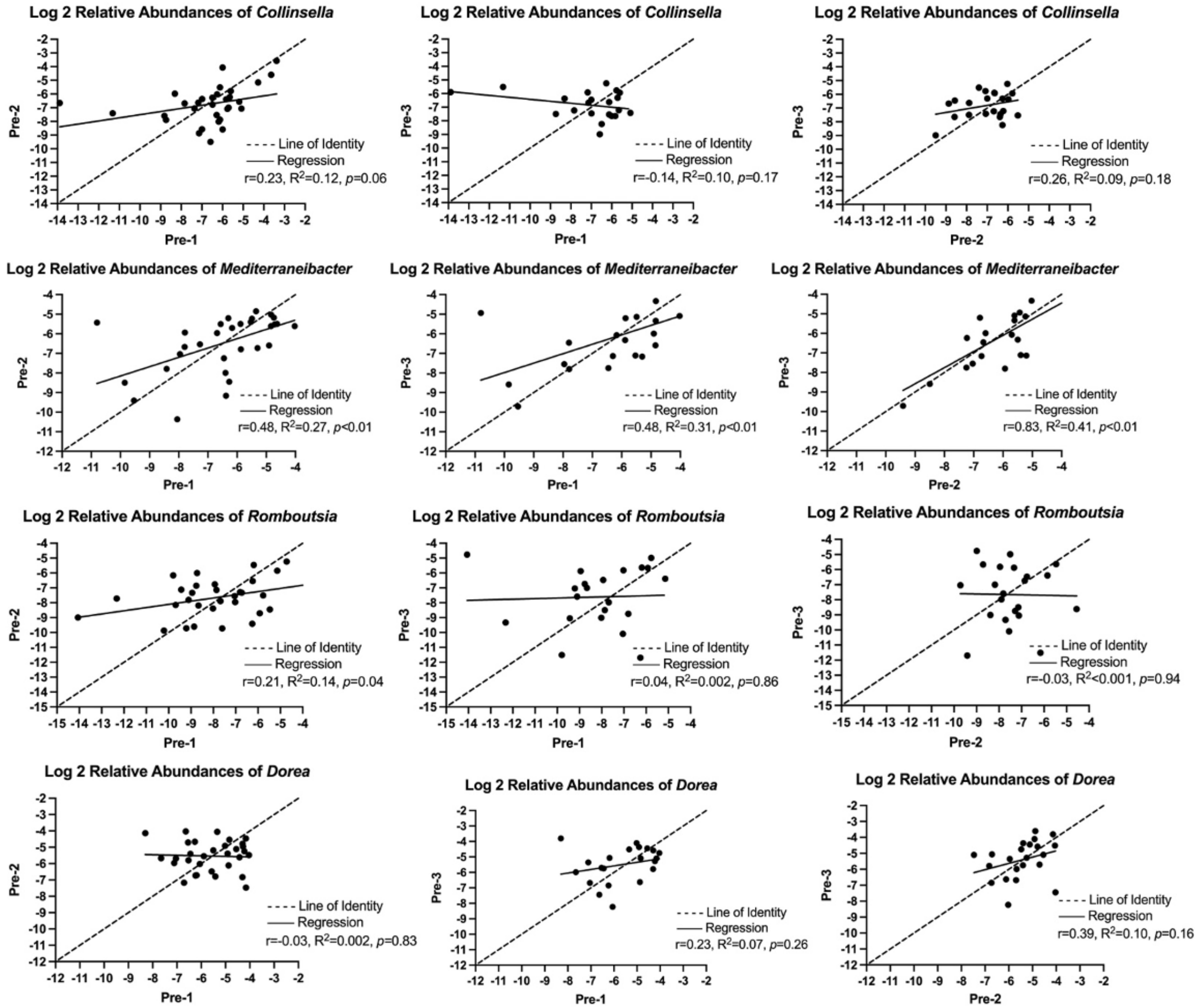

Supplemental Figure S6. Pairwise correlation analyses of selected bacterial genera using pre-HDP values among the 3 HDPs[1].

[1]Pearson's r and R^2 with significance measurement (p<0.05) are included in each plot. HDP, healthy dietary pattern.

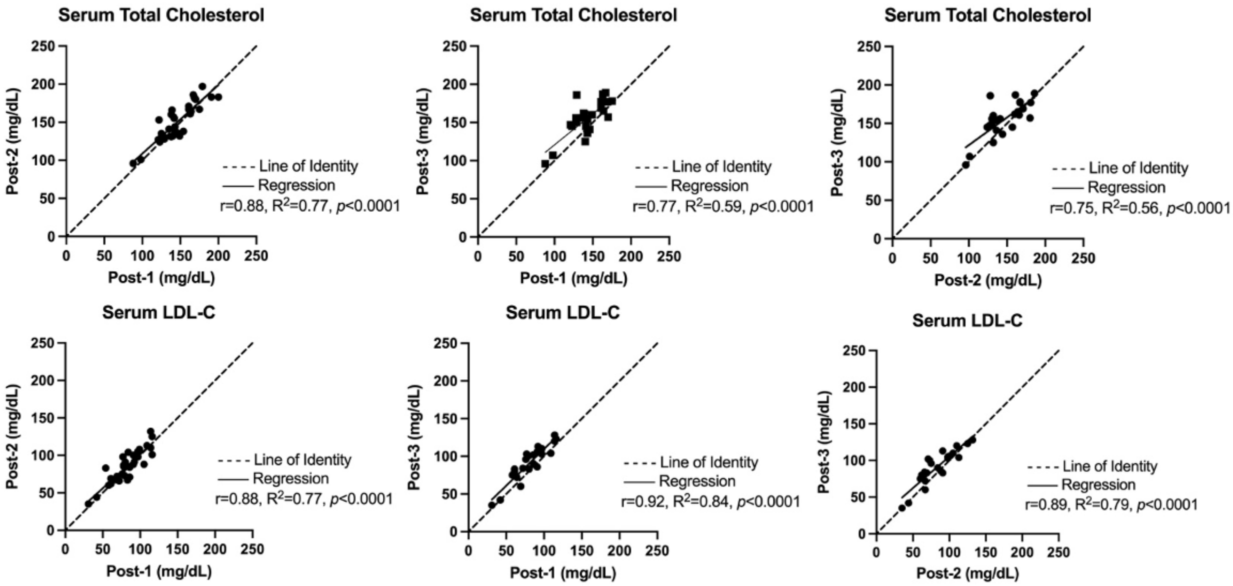

Supplemental Figure S7. Pairwise correlation analyses of selected blood lipids using post-HDP values among the 3 HDPs[1].

[1]Pearson's r and R^2 with significance measurement ($p<0.05$) are included in each plot. Results of correlation analyses using full completers who finished and provided blood lipid data for 3 HDPs (N=12) showed same statistical significance. HDP, healthy dietary pattern.

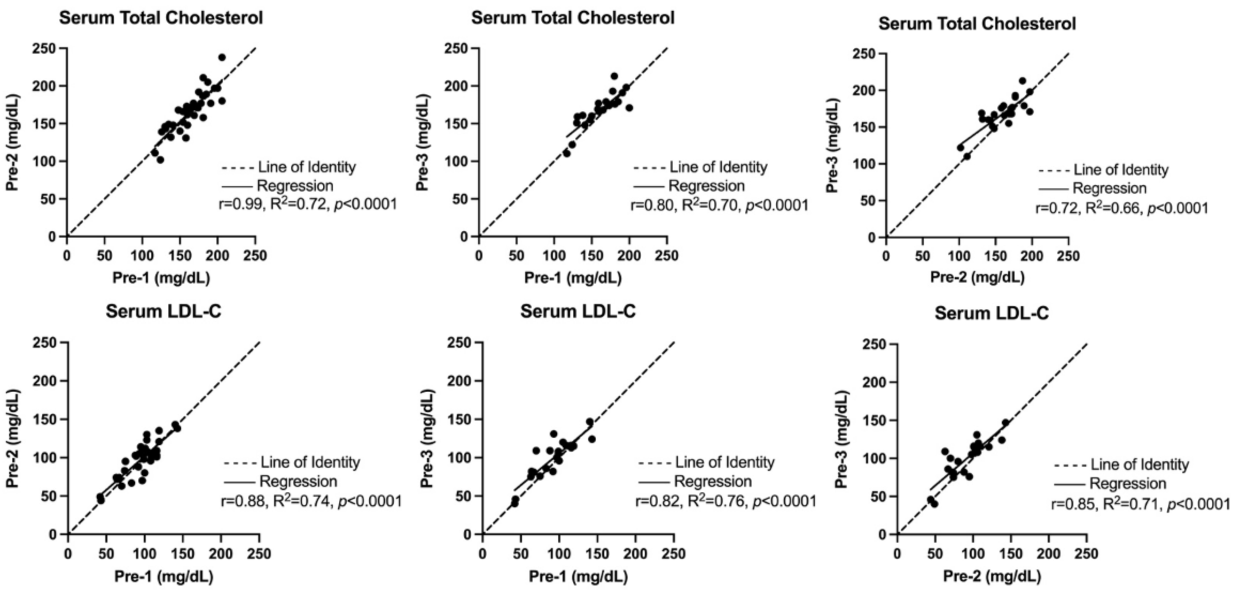

Supplemental Figure S8. Pairwise correlation analyses of selected blood lipids using pre-HDP values among the 3 HDPs[1].

[1]Pearson's r and R^2 with significance measurement ($p<0.05$) are included in each plot. Results of correlation analyses using full completers who finished and provided blood lipid data for 3 HDPs (N=12) showed same statistical significance. HDP, healthy dietary pattern.

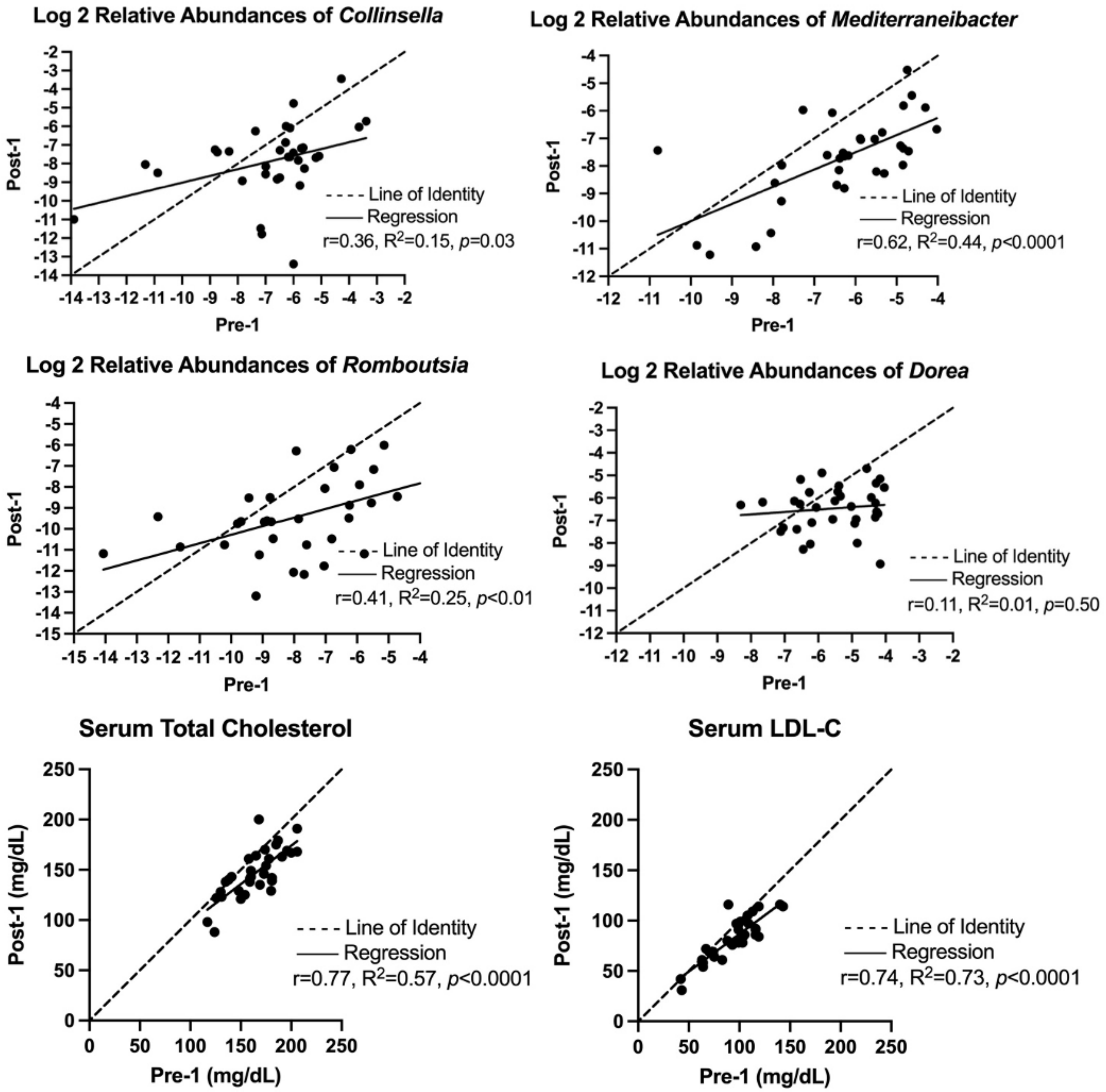

Supplemental Figure S9. Correlation analyses of selected bacterial taxa and blood lipids between pre-1 and post-1[1].

[1]Pearson's r and R^2 with significance measurement (p<0.05) are included in each plot.
