# Supplementary material for: Healthy Dietary Pattern Cycling Affects Gut Microbiota and Cardiovascular Disease Risk Factors: Results from a Randomized Controlled Feeding Trial with Young, Healthy Adults
Source: Nutrients. 2024 Oct 25;16(21):3619. doi: 10.3390/nu16213619 (PMC11547453; doi:10.3390/nu16213619)
Supplement: Supplementary file 1 [file nutrients-16-03619-s001.zip › Supplemental Tables.pdf]

**Healthy Dietary Pattern Cycling Affects Gut Microbiota and Cardiovascular Disease Risk Factors: Results from a Randomized Controlled Feeding Trial with Young, Healthy Adults**

**List of supplemental tables**

Supplemental Table S1. Nutrients of study diets consumed during each HDP and habitual intakes at baseline and washouts. ........ 2

Supplemental Table S2. Daily food group servings from baseline, each HDP (averages of 4-day menus), and washouts, presented in cup or ounce-equivalents[1]. ........ 3

Supplemental Table S3. Alpha diversity assessments. ........ 4

Supplemental Table S4. Beta diversity assessments ........ 7

Supplemental Table S5. Taxa that significantly differed between groups based on LEfSe analyses. ........ 15

Supplemental Table S6. OTUs that significantly differed between groups based on LEfSe analyses. ........ 27

Supplemental Table S7. Comparison of post/pre fold changes of selected taxa with at least 0.1% of relative abundance among HDPs 1-3 and between washouts 1-2. ........ 32

Supplemental Table S8. Comparison of post/pre fold changes of selected OTUs with at least 0.1% of relative abundance among HDPs 1-3 and between washouts 1-2. ........ 33

Supplemental Table S9. Participant responses in cardiovascular risk factors from consuming study diets for 3 times (HDPs 1-3). Results are presented as LS means ± SEM ........ 34

Supplemental Table S10. Raw (unadjusted) means and SD of participant responses in cardiovascular risk factors from consuming study diets for 3 times (HDPs 1-3). ........ 34

Supplemental Table S11. Rho values of significant correlations between changes in blood lipids and bacterial taxa. ........ 35

Supplemental Table S1. Nutrients of study diets consumed during each HDP and habitual intakes at baseline and washouts.

| | Pre-1 (N=12) | SD | Post-1 (N=17) | SD | Pre-2 (N=11) | SD | Post-2 (N=17) | SD | Pre-3 (N=7) | SD | Post-3 (N=12) | SD |
|---|---|---|---|---|---|---|---|---|---|---|---|---|
| **Energy (kcal)** | 2216 | 388 | 2366 | 299 | 2306 | 521 | 2431 | 226 | 2252 | 383 | 2393 | 240 |
| **Total fat (g)** | 84.2 | 18.7 | 68.9 | 9.6 | 107.3 | 31.1 | 72.6 | 6.3 | 82.3 | 21.8 | 70.8 | 6.3 |
| **% Energy from Fat** | 33.5 | 5.3 | 25.4 | 2.0 | 40.7 | 6.6 | 26.2 | 2.4 | 32.3 | 7.2 | 26.0 | 2.6 |
| **Total carbohydrate (g)** | 275.0 | 54.0 | 356.1 | 42.9 | 240.2 | 61.3 | 356.9 | 41.5 | 288.9 | 56.7 | 354.2 | 45.3 |
| **% Energy from Carbohydrate** | 48.7 | 5.4 | 58.3 | 2.7 | 41.7 | 8.2 | 56.7 | 2.8 | 50.8 | 8.3 | 57.1 | 3.1 |
| **Total Protein (g)** | 96.4 | 26.6 | 103.1 | 18.4 | 93.0 | 38.5 | 109.6 | 12.7 | 93.9 | 30.6 | 107.1 | 13.4 |
| **% Energy from Protein** | 17.5 | 3.9 | 16.3 | 1.2 | 15.7 | 3.8 | 17.0 | 0.8 | 16.3 | 3.6 | 16.9 | 0.9 |
| **Cholesterol (mg)** | 375 | 135 | 216 | 47 | 375 | 219 | 239 | 31 | 280 | 196 | 231 | 33 |
| **Saturated Fatty Acids (g)** | 29.9 | 8.5 | 18.6 | 3.5 | 33.7 | 15.1 | 20.1 | 2.3 | 27.7 | 8.3 | 19.4 | 2.4 |
| **% Energy from SFA** | 11.8 | 2.4 | 6.9 | 0.8 | 12.7 | 4.3 | 7.3 | 0.9 | 10.8 | 2.5 | 7.2 | 1.0 |
| **Monounsaturated fatty acids (g)** | 30.2 | 7.2 | 26.0 | 4.0 | 40.5 | 15.2 | 27.7 | 2.8 | 28.0 | 9.1 | 26.9 | 2.8 |
| **% Energy from MUFA** | 12.1 | 2.5 | 9.6 | 1.0 | 15.1 | 3.7 | 10.0 | 1.2 | 11.0 | 3.2 | 9.9 | 1.2 |
| **Polyunsaturated fatty acids (g)** | 16.4 | 7.6 | 16.0 | 1.0 | 23.6 | 12.4 | 16.4 | 0.8 | 19.5 | 8.8 | 16.3 | 0.9 |
| **% Energy from PUFA** | 6.5 | 3.0 | 5.9 | 0.4 | 9.2 | 4.8 | 5.9 | 0.3 | 7.8 | 3.3 | 5.9 | 0.3 |
| **Total Dietary Fiber (g)** | 26.1 | 7.5 | 55.6 | 7.7 | 23.7 | 8.3 | 55.7 | 7.6 | 24.7 | 7.8 | 55.3 | 8.0 |
| **Iron (mg)** | 16.3 | 5.0 | 33.9 | 4.9 | 15.6 | 7.0 | 34.5 | 4.4 | 21.9 | 9.2 | 34.1 | 4.7 |
| **Sodium (mg)** | 3357 | 999 | 2826 | 448 | 3342 | 895 | 3250 | 641 | 3628 | 576 | 3276 | 667 |

**Notes**: HDP, healthy dietary pattern. Total nutrient contents are based on average consumption by participants and presented as means ± standard deviations.

Supplemental Table S2. Daily food group servings from baseline, each HDP (averages of 4-day menus), and washouts, presented in cup or ounce-equivalents[1].

| | Pre-1 | Post-1 | Pre-2 | Post-2 | Pre-3 | Post-3 |
|---|---|---|---|---|---|---|
| **Food group or Subgroup** | N=12 | N=17 | N=11 | N=17 | N=7 | N=12 |
| Vegetables (serving/d) [2] | 2.0 (1.1) | 5.2 (0.7) | 2.2 (1.2) | 5.2 (0.7) | 1.7 (0.9) | 5.1 (0.8) |
| [Dark-Green Vegetables] | 0.4 (0.5) | 1.0 (0.2) | 0.5 (0.7) | 1.0 (0.2) | 0.2 (0.3) | 1.0 (0.2) |
| [Red and Orange Vegetables] | 0.5 (0.4) | 0.7 (0.1) | 0.5 (0.4) | 0.7 (0.1) | 0.4 (0.2) | 0.7 (0.1) |
| [Beans, Peas, Lentils (Legumes)] | 0.2 (0.4) | 1.5 (0.4) | 0.1 (0.2) | 1.5 (0.4) | 0.2 (0.4) | 1.5 (0.4) |
| [Starchy Vegetables] | 0.3 (0.4) | 0.7 (0.1) | 0.4 (0.4) | 0.7 (0.1) | 0.4 (0.5) | 0.7 (0.1) |
| [Other Vegetables] | 0.7 (0.9) | 1.3 (0.1) | 0.8 (0.6) | 1.3 (0.1) | 0.5 (0.6) | 1.2 (0.1) |
| Fruits (serving/d) [2] | 1.7 (1.6) | 2.1 (0.2) | 0.6 (0.8) | 2.1 (0.2) | 0.8 (1.1) | 2.1 (0.2) |
| Grains (serving/d) [3] | 7.5 (3.0) | 7.4 (0.7) | 6.7 (2.5) | 7.4 (0.6) | 9.2 (3.5) | 7.4 (0.7) |
| [Whole Grains] | 2.4 (2.7) | 3.5 (0.3) | 1.8 (1.6) | 3.5 (0.3) | 1.8 (1.5) | 3.5 (0.3) |
| [Refined Grains] | 5.1 (4.0) | 3.9 (0.4) | 4.9 (2.5) | 4.0 (0.4) | 7.5 (4.0) | 3.9 (0.5) |
| Dairy (serving/d) [4] | 2.9 (1.4) | 2.5 (0.2) | 2.0 (1.7) | 2.5 (0.2) | 2.8 (0.9) | 2.4 (0.3) |
| Protein Foods(serving/d) [5] | 6.5 (2.8) | 5.9 (1.8) | 8.3 (4.9) | 7.0 (1.2) | 5.4 (2.0) | 6.7 (1.4) |
| [Meats, Poultry, Eggs] | 4.7 (2.3) | 2.1 (1.6) | 5.4 (4.6) | 3.2 (1.2) | 4.4 (2.1) | 3.0 (1.3) |
| -Red meat | 2.1 (1.8) | 1.2 (1.4) | 3.2 (3.9) | 1.5 (1.2) | 0.8 (0.6) | 1.2 (1.1) |
| -Poultry | 1.7 (2.4) | 0 | 1.0 (1.0) | 0 | 2.5 (1.4) | 0 |
| -Eggs | 0.8 (0.8) | 0.7 (0.1) | 0.8 (0.9) | 0.7 (0.1) | 0.5 (0.8) | 0.7 (0.1) |
| -Other [6] | 0.2 (0.4) | 0.2 (0.7) | 0.4 (0.7) | 1.0 (1.1) | 0.6 (0.8) | 1.0 (1.1) |
| [Seafood] | 1.0 (1.4) | 0 | 0.6 (1.6) | 0 | 0.2 (0.4) | 0 |
| [Nuts, Seed, Soy Products] | 0.8 (1.5) | 3.8 (0.4) | 2.3 (3.2) | 3.8 (0.4) | 0.9 (1.3) | 3.7 (0.5) |
| Oils (serving/day) [7] | 2.0 (0.9) | 2.5 (0.3) | 3.8 (2.2) | 2.5 (0.3) | 3.1 (2.8) | 2.4 (0.3) |
| Calories for Other Uses | 7.4 (3.3) | 0.9 (0.1) | 8.1 (3.7) | 0.9 (0.1) | 6.0 (2.7) | 0.9 (0.1) |
| 2015-HEI Score [8] | 55.5 (13.1) | 80.9 (2.1) | 50.5 (9.2) | 78.6 (3.0) | 44.0 (15.6) | 78.4 (3.1) |

Notes:

HDP, healthy dietary pattern.

[1] Food group servings based on the 2015-2020 Dietary Guidelines for Americans presented for three study diets with averaged consumed amount across a 4-d menu cycle.

[2] 1 cup equivalent: 1 cup raw or cooked vegetable or fruit, 1 cup vegetable or fruit juice, 2 cups leafy salad greens, ½ cup dried fruit or vegetable.

[3] 1 ounce-equivalent: ½ cup cooked rice, pasta, or cereal; 1 ounce dry pasta or rice; 1 medium (1 ounce) slice bread; 1 ounce of ready-to-eat cereal (about 1 cup of flaked cereal).

[4] 1 cup-equivalent: 1 cup milk, yogurt, or fortified soymilk; 1½ ounces natural cheese such as cheddar cheese or 2 ounces of processed cheese.

[5] 1 ounce-equivalent is: 1 ounce lean meat, poultry, or seafood; 1 egg; ¼ cup cooked beans or tofu; 1 Tbsp peanut butter; ½ ounce nuts or seeds.

[6] "Other Meat" includes processed meat products such as cold cuts and sausages.

[7] 1 serving: 1 teaspoon for margarine, oil, shortening, butter, and other animal fats; 30 grams for poured salad dressing; and 15 grams for mayonnaise or mayonnaise type dressing.

[8] The 2015-HEI score for participants habitual diets was calculated for the first baseline (N=33) and presented as mean (standard deviation).

Supplemental Table S3. Alpha diversity assessments.

| Diet | Time | N participants | N samples | Chao (m) | Chao (SD) | ACE (m) | ACE (SD) | Invsimpson (m) | Invsimpson (SD) | Shannon (m) | Shannon (SD) | p-values (fdr adjusted for wilcoxon test) | Chao | ACE | Invsimpson | Shannon |
|---|---|---|---|---|---|---|---|---|---|---|---|---|---|---|---|---|
| **Adoption of HEP: pre versus post with three HDPs combined** | | | | | | | | | | | | | | | | |
| All | pre | 17 | 87 | 207.97 | 49.53 | 225.49 | 55.18 | 20.78 | 8.07 | 3.60 | 0.36 | **post-pre pair-wise wilcoxon test** | 0.34 | 0.19 | 0.18 | 0.40 |
| All | post | 17 | 88 | 219.33 | 57.41 | 240.76 | 70.60 | 18.96 | 7.88 | 3.54 | 0.40 | | | | | |
| **Abandonment of HEP: post-previous-HDP versus pre-next-HDP with two washouts combined** | | | | | | | | | | | | | | | | |
| All | post-previous-HDP | | 65 | 228.62 | 58.61 | 250.53 | 72.12 | 19.33 | 7.87 | 3.59 | 0.35 | **post-pre pair-wise wilcoxon test** | 0.001 | 0.0004 | 0.61 | 0.05 |
| All | pre-next-HDP | | 54 | 202.90 | 50.13 | 217.76 | 52.62 | 19.81 | 7.39 | 3.57 | 0.35 | | | | | |
| **1st Adoption of HEP** | | | | | | | | | | | | | | | | |
| HDP 1 | pre-1 | | 33 | 214.86 | 47.83 | 236.42 | 55.98 | 22.39 | 8.98 | 3.64 | 0.38 | **post-pre pair-wise wilcoxon test** | 0.55 | 0.50 | 0.12 | 0.31 |
| HDP 1 | post-1 | | 34 | 224.48 | 44.18 | 245.98 | 53.33 | 19.55 | 8.23 | 3.59 | 0.33 | | | | | |
| **1st Abandonment of HEP** | | | | | | | | | | | | | | | | |
| Washout 1 | pre-2 | | 34 | 224.48 | 44.18 | 245.98 | 53.33 | 19.55 | 8.23 | 3.59 | 0.33 | **post-pre pair-wise wilcoxon test** | 0.52 | 0.46 | 0.49 | 0.44 |
| Washout 1 | post-1 | | 32 | 214.67 | 51.99 | 232.76 | 55.11 | 20.81 | 7.46 | 3.65 | 0.33 | | | | | |
| **2nd Adoption of HEP** | | | | | | | | | | | | | | | | |
| HDP 2 | pre-2 | | 32 | 214.67 | 51.99 | 232.76 | 55.11 | 20.81 | 7.46 | 3.65 | 0.33 | **post-pre pair-wise wilcoxon test** | 0.42 | 0.52 | 0.35 | 0.64 |
| HDP 2 | post-2 | | 31 | 233.16 | 71.70 | 255.51 | 88.99 | 19.09 | 7.58 | 3.59 | 0.37 | | | | | |
| **2nd Abandonment of HEP** | | | | | | | | | | | | | | | | |
| Washout 2 | pre-3 | | 31 | 233.16 | 71.70 | 255.51 | 88.99 | 19.09 | 7.58 | 3.59 | 0.37 | **post-pre pair-wise wilcoxon test** | 0.007 | 0.001 | 0.74 | 0.19 |
| Washout 2 | post-2 | | 22 | 185.78 | 42.85 | 195.95 | 40.80 | 18.35 | 7.19 | 3.47 | 0.35 | | | | | |
| **3rd Adoption of HEP** | | | | | | | | | | | | | | | | |
| HDP 3 | pre-3 | | 22 | 185.78 | 42.85 | 195.95 | 40.80 | 18.35 | 7.19 | 3.47 | 0.35 | **post-pre pair-wise wilcoxon test** | 0.73 | 0.32 | 0.96 | 0.99 |
| HDP 3 | post-3 | | 23 | 192.45 | 45.46 | 211.43 | 55.82 | 17.96 | 8.01 | 3.40 | 0.49 | | | | | |
| **among three HDPs at pre** | | | | | | | | | | | | **Kruskall-wallis test among 3 diets-pre** | 0.07 | 0.01 | 0.14 | 0.12 |
| **Pair-wise comparisons at pre for ACE** | | | | | | | | | | | | | | | | |
| **HDP 1 vs HDP 2** | 0.881 | **HDP 1 vs HDP 3** | 0.014 | **HDP 2 vs HDP 3** | 0.023 | | | | | | | | | | | |

Supplemental Table S3. continued

| Diet | Time | N participants | N samples | Chao (m) | Chao (SD) | ACE (m) | ACE (SD) | Invsimpson (m) | Invsimpson (SD) | Shannon (m) | Shannon (SD) | p-values (fdr adjusted for wilcoxon test) | Chao | ACE | Invsimpson | Shannon |
|---|---|---|---|---|---|---|---|---|---|---|---|---|---|---|---|---|
| **among three HDPs at post** | | | | | | | | | | | | **Kruskall-wallis test among 3 diets-post** | 0.01 | 0.03 | 0.93 | 0.45 |
| **Pair-wise comparisons at post for Chao** | | | | | | | | | | | | | | | | |
| **HDP 1 vs HDP 2** | 0.943 | **HDP 1 vs HDP 3** | 0.032 | **HDP 2 vs HDP 3** | 0.032 | | | | | | | | | | | |
| **Pair-wise comparisons at post for ACE** | | | | | | | | | | | | | | | | |
| **HDP 1 vs HDP 2** | **0.984** | **HDP 1 vs HDP 3** | 0.014 | **HDP 2 vs HDP 3** | 0.014 | | | | | | | | | | | |
| **post-pre changes among three HDPs** | | | | | | | | | | | | | | | | |
| HDP 1 | post-pre change | 17 | | 11.53 | 28.06 | 12.17 | 45.52 | -0.05 | 0.28 | -2.92 | 6.69 | | | | | |
| HDP 2 | post-pre change | 16 | | 15.49 | 44.43 | 18.79 | 59.38 | -0.06 | 0.27 | -1.72 | 6.50 | | | | | |
| HDP 3 | post-pre change | 12 | | 11.37 | 26.15 | 18.93 | 31.53 | -0.02 | 0.39 | 0.55 | 7.15 | **Kruskall-wallis test among 3 diets** | 0.94 | 0.73 | 0.71 | 0.43 |
| **post-pre changes between two washouts** | | | | | | | | | | | | | | | | |
| Abandonment 1 | post-pre change | 17 | | -11.77 | 26.39 | -15.11 | 36.75 | 0.05 | 0.26 | 1.30 | 6.92 | | | | | |
| Abandonment 2 | post-pre change | 11 | | -29.12 | 54.33 | -42.65 | 71.49 | -0.01 | 0.18 | 1.01 | 3.35 | **pair-wise wilcoxon test** | 0.58 | 0.38 | 0.78 | 0.58 |
| **Other comparisons** | | | | | | | | | | | | | | | | |
| pre1 versus pre2 versus post1 versus post2 with three HDPs combined | | | | | | | | | | | | | | | | |
| All | pre1 | 17 | 43 | 206.37 | 44.14 | 222.04 | 46.01 | 19.99 | 8.58 | 3.58 | 0.35 | | | | | |
| All | pre2 | 17 | 44 | 209.54 | 54.75 | 228.87 | 63.24 | 21.56 | 7.56 | 3.62 | 0.37 | **pre1-pre2 pair-wise wilcoxon test** | 0.98 | 0.92 | 0.17 | 0.44 |
| All | post1 | 17 | 44 | 220.88 | 60.69 | 245.39 | 76.36 | 19.33 | 8.85 | 3.55 | 0.41 | | | | | |
| All | post2 | 17 | 44 | 217.79 | 54.60 | 236.13 | 64.88 | 18.60 | 6.85 | 3.53 | 0.39 | **post1-post2 pair-wise wilcoxon test** | 0.81 | 0.72 | 0.91 | 1.00 |

Supplemental Table S3. continued

| Diet | Time | N participants | N samples | Chao (m) | Chao (SD) | ACE (m) | ACE (SD) | Invsimpson (m) | Invsimpson (SD) | Shannon (m) | Shannon (SD) | p-values (fdr adjusted for wilcoxon test) | Chao | ACE | Invsimpson | Shannon |
|---|---|---|---|---|---|---|---|---|---|---|---|---|---|---|---|---|
| pre1 versus pre2 versus post1 versus post2 within each HDP | | | | | | | | | | | | | | | | |
| HDP 1 | pre1 | | 17 | 209.84 | 40.85 | 230.24 | 45.05 | 22.74 | 11.13 | 3.64 | 0.41 | | | | | |
| HDP 1 | pre2 | | 16 | 220.19 | 55.15 | 242.98 | 66.57 | 22.02 | 6.32 | 3.63 | 0.37 | | | | | |
| HDP 1 | post1 | | 17 | 222.90 | 44.41 | 245.75 | 61.44 | 18.56 | 10.37 | 3.52 | 0.38 | | | | | |
| HDP 1 | post2 | | 17 | 226.07 | 45.26 | 246.21 | 45.72 | 20.53 | 5.49 | 3.66 | 0.26 | **Kruskall-wallis test among 4 timepoints** | 0.93 | 0.88 | 0.23 | 0.54 |
| HDP 2 | pre1 | | 15 | 213.69 | 52.39 | 228.92 | 52.02 | 18.61 | 4.33 | 3.59 | 0.31 | | | | | |
| HDP 2 | pre2 | | 17 | 215.53 | 53.22 | 236.14 | 59.08 | 22.75 | 9.11 | 3.70 | 0.35 | | | | | |
| HDP 2 | post1 | | 16 | 242.84 | 78.08 | 269.83 | 95.38 | 21.21 | 8.04 | 3.66 | 0.40 | | | | | |
| HDP 2 | post2 | | 15 | 222.84 | 65.28 | 240.24 | 82.09 | 16.82 | 6.58 | 3.51 | 0.34 | **Kruskall-wallis test among 4 timepoints** | 0.78 | 0.69 | 0.16 | 0.36 |
| HDP 3 | pre1 | | 11 | 189.70 | 35.27 | 198.50 | 32.75 | 17.63 | 7.97 | 3.47 | 0.33 | | | | | |
| HDP 3 | pre2 | | 11 | 181.87 | 50.77 | 193.40 | 49.07 | 19.07 | 6.63 | 3.47 | 0.39 | | | | | |
| HDP 3 | post1 | | 11 | 184.62 | 38.13 | 204.37 | 44.68 | 17.81 | 7.67 | 3.41 | 0.44 | | | | | |
| HDP 3 | post2 | | 12 | 199.63 | 51.90 | 217.90 | 65.74 | 18.11 | 8.65 | 3.39 | 0.56 | **Kruskall-wallis test among 4 timepoints** | 0.77 | 0.64 | 0.93 | 1.00 |

**Note:** HDP, healthy dietary pattern. Chao, Chao1 richness index; ACE, abundance-based coverage estimator richness index; Shannon, Shannon diversity index; Invsimpson, Inverse Simpson diversity index; m, mean; SD, standard deviation.

Supplemental Table S4. Beta diversity assessments

| | | | | | | | | | |
|---|---|---|---|---|---|---|---|---|---|
| | **pre vs post three HDPs combined** | | | **pre vs post two washouts combined** | | | | | |
| **pcoa axes** | **thetayc** | **jclass** | **braycurtis** | **thetayc** | **jclass** | **braycurtis** | | | |
| rsq 1 axis | 0.23 | 0.49 | 0.24 | 0.19 | 0.43 | 0.24 | | | |
| rsq 2 axis | 0.40 | 0.60 | 0.42 | 0.39 | 0.53 | 0.45 | | | |
| rsq 3 axis | 0.43 | 0.63 | 0.63 | 0.50 | 0.57 | 0.63 | | | |
| | **pre all samples** | | | **post all samples** | | | | | |
| **pcoa axes** | **thetayc** | **jclass** | **braycurtis** | **thetayc** | **jclass** | **braycurtis** | | | |
| rsq 1 axis | 0.23 | 0.49 | 0.32 | 0.26 | 0.47 | 0.17 | | | |
| rsq 2 axis | 0.40 | 0.57 | 0.49 | 0.44 | 0.59 | 0.39 | | | |
| rsq 3 axis | 0.45 | 0.58 | 0.55 | 0.56 | 0.63 | 0.63 | | | |
| | **pre vs post HDP 1** | | | **pre vs post HDP 2** | | | **pre vs post HDP 3** | | |
| **pcoa axes** | **thetayc** | **jclass** | **braycurtis** | **thetayc** | **jclass** | **braycurtis** | **thetayc** | **jclass** | **braycurtis** |
| rsq 1 axis | 0.29 | 0.50 | 0.24 | 0.19 | 0.52 | 0.20 | 0.24 | 0.48 | 0.41 |
| rsq 2 axis | 0.43 | 0.64 | 0.45 | 0.35 | 0.62 | 0.36 | 0.52 | 0.58 | 0.60 |
| rsq 3 axis | 0.49 | 0.64 | 0.62 | 0.40 | 0.66 | 0.64 | 0.61 | 0.68 | 0.70 |
| | **pre vs post Washout 1** | | | **pre vs post Washout 2** | | | | | |
| **pcoa axes** | **thetayc** | **jclass** | **braycurtis** | **thetayc** | **jclass** | **braycurtis** | | | |
| rsq 1 axis | 0.23 | 0.52 | 0.32 | 0.18 | 0.46 | 0.24 | | | |
| rsq 2 axis | 0.38 | 0.62 | 0.49 | 0.39 | 0.55 | 0.45 | | | |
| rsq 3 axis | 0.45 | 0.66 | 0.56 | 0.48 | 0.58 | 0.64 | | | |

Supplemental Table S4. continued

| | | | | | | | | |
|---|---|---|---|---|---|---|---|---|
| | **pre vs post three HDPs combined** | | | | **pre vs post two washouts combined** | | | |
| | post-pre | Among | Within | Total | post-pre | Among | Within | Total |
| | SS | 0.55 | 53.48 | 54.03 | SS | 0.84 | 32.98 | 33.82 |
| | df | 1 | 173 | 174 | df | 1 | 104 | 105 |
| | MS | 0.55 | 0.31 | | MS | 0.84 | 0.32 | |
| | Fs: | 1.78 | | | Fs: | 2.65 | | |
| **amova-thetayc** | p-value: | 0.043* | | | p-value: | 0.004* | | |
| | post-pre | Among | Within | Total | post-pre | Among | Within | Total |
| | SS | 0.27 | 44.59 | 44.85 | SS | 0.62 | 26.44 | 27.06 |
| | df | 1 | 173 | 174 | df | 1 | 104 | 105 |
| | MS | 0.27 | 0.26 | | MS | 0.62 | 0.25 | |
| | Fs: | 1.04 | | | Fs: | 2.43 | | |
| **amova-jclass** | p-value: | 0.37 | | | p-value: | <0.001* | | |
| | post-pre | Among | Within | Total | post-pre | Among | Within | Total |
| | SS | 0.43 | 37.97 | 38.39 | SS | 0.74 | 23.41 | 24.15 |
| | df | 1 | 173 | 174 | df | 1 | 104 | 105 |
| | MS | 0.43 | 0.22 | | MS | 0.74 | 0.23 | |
| | Fs: | 1.95 | | | Fs: | 3.29 | | |
| **amova-braycurtis** | p-value: | 0.011* | | | p-value: | <0.001* | | |
| | **pre among HDP 1 vs HDP 2 vs HDP 3** | | | | **post among HDP 1 vs HDP 2 vs HDP 3** | | | |
| | preHDP1-preHDP2-preHDP3 | Among | Within | Total | postHDP1-postHDP2-postHDP3 | Among | Within | Total |
| | SS | 0.28 | 25.41 | 25.69 | SS | 0.40 | 27.40 | 27.79 |
| | df | 2.00 | 84.00 | 86.00 | df | 2.00 | 85.00 | 87.00 |
| | MS | 0.14 | 0.30 | | MS | 0.20 | 0.32 | |
| | Fs: | 0.46 | | | Fs: | 0.62 | | |
| **amova-thetayc** | p-value: | 1.00 | | | p-value: | 0.94 | | |

Supplemental Table S. 4.4 continued

| | | | | | | | | | | | | |
|---|---|---|---|---|---|---|---|---|---|---|---|---|
| | preHDP1-preHDP2-preHDP3 | Among | Within | Total | postHDP1-postHDP2-postHDP3 | Among | Within | Total | | | | |
| | SS | 0.41 | 21.48 | 21.89 | SS | 0.41 | 22.09 | 22.50 | | | | |
| | df | 2 | 84 | 86 | df | 2 | 85 | 87 | | | | |
| | MS | 0.20 | 0.26 | | MS | 0.20 | 0.26 | | | | | |
| | Fs: | 0.80 | | | Fs: | 0.79 | | | | | | |
| **amova-jclass** | p-value: | 0.95 | | | p-value: | 0.95 | | | | | | |
| | post-pre | Among | Within | Total | postHDP1-postHDP2-postHDP3 | Among | Within | Total | | | | |
| | SS | 0.24 | 17.92 | 18.16 | SS | 0.32 | 19.49 | 19.81 | | | | |
| | df | 2.00 | 84.00 | 86.00 | df | 2.00 | 85.00 | 87.00 | | | | |
| | MS | 0.12 | 0.21 | | MS | 0.16 | 0.23 | | | | | |
| | Fs: | 0.56 | | | Fs: | 0.69 | | | | | | |
| **amova-braycurtis** | p-value: | 0.99 | | | p-value: | 0.91 | | | | | | |
| | **pre1-pre2-post1-post2 HDP 1** | | | | **pre1-pre2-post1-post2 HDP 2** | | | | **pre1-pre2-post1-post2 HDP 3** | | | |
| | post1-post2-pre1-pre2 | Among | Within | Total | post1-post2-pre1-pre2 | Among | Within | Total | post1-post2-pre1-pre2 | Among | Within | Total |
| | SS | 0.42 | 19.82 | 20.24 | SS | 0.63 | 19.18 | 19.80 | SS | 0.43 | 13.17 | 13.60 |
| | df | 3.00 | 63.00 | 66.00 | df | 3.00 | 59.00 | 62.00 | df | 3.00 | 41.00 | 44.00 |
| | MS | 0.14 | 0.31 | | MS | 0.21 | 0.33 | | MS | 0.14 | 0.32 | |
| | Fs: | 0.45 | | | Fs: | 0.64 | | | Fs: | 0.45 | | |
| **amova-thetayc** | p-value: | 1.00 | | | p-value: | 0.98 | | | p-value: | 1.00 | | |

Supplemental Table S4. continued

| | | | | | | | | | | | | |
|---|---|---|---|---|---|---|---|---|---|---|---|---|
| | post1-post2-pre1-pre2 | Among | Within | Total | post1-post2-pre1-pre2 | Among | Within | Total | post1-post2-pre1-pre2 | Among | Within | Total |
| | SS | 0.44 | 16.61 | 17.05 | SS | 0.46 | 15.63 | 16.09 | SS | 0.43 | 10.53 | 10.95 |
| | df | 3 | 63 | 66 | df | 3 | 59 | 62 | df | 3 | 41 | 44 |
| | MS | 0.15 | 0.26 | | MS | 0.15 | 0.26 | | MS | 0.14 | 0.26 | |
| | Fs: | 0.55 | | | Fs: | 0.58 | | | Fs: | 0.56 | | |
| **amova-jclass** | p-value: 1 | | | | p-value: 1 | | | | p-value: 1 | | | |
| | post1-post2-pre1-pre2 | Among | Within | Total | post1-post2-pre1-pre2 | Among | Within | Total | post1-post2-pre1-pre2 | Among | Within | Total |
| | SS | 0.36 | 14.14 | 14.50 | SS | 0.44 | 13.58 | 14.02 | SS | 0.33 | 9.18 | 9.51 |
| | df | 3 | 63 | 66 | df | 3 | 59 | 62 | df | 3 | 41 | 44 |
| | MS | 0.12 | 0.22 | | MS | 0.15 | 0.23 | | MS | 0.11 | 0.22 | |
| | Fs: | 0.53 | | | Fs: | 0.64 | | | Fs: | 0.49 | | |
| **amova-braycurtis** | p-value: | 1 | | | p-value: | 0.99 | | | p-value: | 1 | | |
| | **pre1-pre2-post1-post2 Washout 1** | | | | **pre1-pre2-post1-post2 Washout 2** | | | | | | | |
| | post1-post2-pre1-pre2 | Among | Within | Total | post1-post2-pre1-pre2 | Among | Within | Total | | | | |
| | SS | 0.44 | 19.40 | 19.84 | SS | 0.73 | 16.18 | 16.91 | | | | |
| | df | 3 | 62 | 65 | df | 3 | 49 | 52 | | | | |
| | MS | 0.15 | 0.31 | | MS | 0.24 | 0.33 | | | | | |
| | Fs: | 0.47 | | | Fs: | 0.74 | | | | | | |
| **amova-thetayc** | p-value: | 1.00 | | | p-value: | 0.92 | | | | | | |
| | post1-post2-pre1-pre2 | Among | Within | Total | post1-post2-pre1-pre2 | Among | Within | Total | | | | |
| | SS | 0.46 | 16.17 | 16.63 | SS | 0.60 | 12.90 | 13.50 | | | | |
| | df | 3 | 62 | 65 | df | 3 | 49 | 52 | | | | |
| | MS | 0.15 | 0.26 | | MS | 0.20 | 0.26 | | | | | |
| | Fs: | 0.59 | | | Fs: | 0.76 | | | | | | |
| **amova-jclass** | p-value: 1 | | | | p-value: | 1.00 | | | | | | |

Supplemental Table S4. continued

| | | | | | | | | | |
|---|---|---|---|---|---|---|---|---|---|
| | post1-post2-pre1-pre2 | Among | Within | Total | post1-post2-pre1-pre2 | Among | Within | Total | |
| | SS | 0.38 | 13.43 | 13.81 | SS | 0.58 | 11.49 | 12.08 | |
| | df | 3 | 62 | 65 | df | 3 | 49 | 52 | |
| | MS | 0.13 | 0.22 | | MS | 0.19 | 0.23 | | |
| | Fs: | 0.58 | | | Fs: | 0.83 | | | |
| **amova-braycurtis** | p-value: | 1.00 | | | p-value: | 0.83 | | | |
| **pre vs post among HDP 1 vs HDP 2 vs HDP 3** | | | | | | | | | |
| **homova-thetayc** | | | | | | | | | |
| HOMOVA | BValue | P-value | SSwithin/(Ni-1)_values | | | | | | |
| | | | postHDP1 | postHDP 2 | postHDP3 | preHDP1 | preHDP 2 | preHDP 3 | |
| | 0.13 | 0.71 | 0.31 | 0.34 | 0.32 | 0.30 | 0.30 | 0.31 | |
| **homova-jclass** | | | | | | | | | |
| HOMOVA | BValue | P-value | SSwithin/(Ni-1)_values | | | | | | |
| | | | postHDP1 | postHDP 2 | postHDP3 | preHDP1 | preHDP 2 | preHDP 3 | |
| | 0.04 | 0.67 | 0.26 | 0.27 | 0.25 | 0.26 | 0.26 | 0.25 | |
| **homova-braycurtis** | | | | | | | | | |
| HOMOVA | BValue | P-value | SSwithin/(Ni-1)_values | | | | | | |
| | | | postHDP1 | postHDP 2 | postHDP3 | preHDP1 | preHDP 2 | preHDP 3 | |
| | 0.25 | 0.40 | 0.22 | 0.24 | 0.23 | 0.22 | 0.21 | 0.21 | |
| **pre vs post three HDPs combined** | | | | | **pre vs post two washouts combined** | | | | |
| **homova-thetayc** | | | | | **homova-thetayc** | | | | |
| HOMOVA | BValue | P-value | SSwithin/(Ni-1)_values | | HOMOVA | BValue | P-value | SSwithin/(Ni-1)_values | |
| | | | post | pre | | | | post | pre |
| | 0.09 | 0.16 | 0.32 | 0.30 | | 0.12 | 0.07 | 0.33 | 0.30 |

Supplemental Table S4. continued

| | | | | | | | | | |
|---|---|---|---|---|---|---|---|---|---|
| **homova-jclass** | | | | | **homova-jclass** | | | | |
| HOMOVA | BValue | P-value | SSwithin/(Ni-1)_values | | HOMOVA | BValue | P-value | SSwithin/(Ni-1)_values | |
| | | | post | pre | | | | post | pre |
| | 0.01 | 0.53 | 0.26 | 0.26 | | 0.07 | 0.015* | 0.26 | 0.24 |
| **homova-braycurtis** | | | | | **homova-braycurtis** | | | | |
| HOMOVA | BValue | P-value | SSwithin/(Ni-1)_values | | HOMOVA | BValue | P-value | SSwithin/(Ni-1)_values | |
| | | | post | pre | | | | post | pre |
| | 0.12 | 0.14 | 0.23 | 0.21 | | 0.32 | 0.01* | 0.24 | 0.20 |
| **pre1-pre2-post1-post2 HDP 1** | | | | | | | | | |
| **homova-thetayc** | | | | | | | | | |
| HOMOVA | BValue | P-value | SSwithin/(Ni-1)_values | | | | | | |
| | | | post1 | post2 | pre1 | pre2 | | | |
| | 0.02 | 0.93 | 0.33 | 0.31 | 0.30 | 0.32 | | | |
| **homova-jclass** | | | | | | | | | |
| HOMOVA | BValue | P-value | SSwithin/(Ni-1)_values | | | | | | |
| | | | post1 | post2 | pre1 | pre2 | | | |
| post1-post2-pre1-pre2 | 0.01 | 0.80 | 0.26 | 0.26 | 0.26 | 0.27 | | | |
| **homova-braycurtis** | | | | | | | | | |
| HOMOVA | BValue | P-value | SSwithin/(Ni-1)_values | | | | | | |
| | | | post1 | post2 | pre1 | pre2 | | | |
| | 0.07 | 0.73 | 0.23 | 0.22 | 0.21 | 0.24 | | | |
| **pre1-pre2-post1-post2 Washout 1** | | | | | | | | | |
| **homova-thetayc** | | | | | | | | | |
| HOMOVA | BValue | P-value | SSwithin/(Ni-1)_values | | | | | | |
| | | | post1 | post2 | pre1 | pre2 | | | |
| post1-post2-pre1-pre2 | 0.04 | 0.88 | 0.33 | 0.31 | 0.29 | 0.32 | | | |

Supplemental Table S4. continued

| | | | | | | |
|---|---|---|---|---|---|---|
| **homova-jclass** | | | | | | |
| HOMOVA | BValue | P-value | SSwithin/(Ni-1)_values | | | |
| | | | post1 | post2 | pre1 | pre2 |
| post1-post2-pre1-pre2 | 0.003 | 0.96 | 0.26 | 0.26 | 0.26 | 0.26 |
| **homova-braycurtis** | | | | | | |
| HOMOVA | BValue | P-value | SSwithin/(Ni-1)_values | | | |
| | | | post1 | post2 | pre1 | pre2 |
| post1-post2-pre1-pre2 | 0.03 | 0.88 | 0.23 | 0.22 | 0.21 | 0.21 |
| **pre1-pre2-post1-post2 HDP 2** | | | | | | |
| **homova-thetayc** | | | | | | |
| HOMOVA | BValue | P-value | SSwithin/(Ni-1)_values | | | |
| | | | post1 | post2 | pre1 | pre2 |
| post1-post2-pre1-pre2 | 0.13 | 0.40 | 0.33 | 0.36 | 0.30 | 0.32 |
| **homova-jclass** | | | | | | |
| HOMOVA | BValue | P-value | SSwithin/(Ni-1)_values | | | |
| | | | post1 | post2 | pre1 | pre2 |
| post1-post2-pre1-pre2 | 0.02 | 0.62 | 0.27 | 0.27 | 0.26 | 0.26 |
| **homova-braycurtis** | | | | | | |
| HOMOVA | BValue | P-value | SSwithin/(Ni-1)_values | | | |
| | | | post1 | post2 | pre1 | pre2 |
| post1-post2-pre1-pre2 | 0.21 | 0.24 | 0.24 | 0.26 | 0.21 | 0.21 |
| **pre1-pre2-post1-post2 Washout 2** | | | | | | |
| **homova-thetayc** | | | | | | |
| HOMOVA | BValue | P-value | SSwithin/(Ni-1)_values | | | |
| | | | post1 | post2 | pre1 | pre2 |
| | 0.11 | 0.45 | 0.33 | 0.36 | 0.33 | 0.29 |

Supplemental Table S4. continued

| | | | | | | |
|---|---|---|---|---|---|---|
| **homova-jclass** | | | | | | |
| HOMOVA | BValue | P-value | SSwithin/(Ni-1)_values | | | |
| | | | post1 | post2 | pre1 | pre2 |
| | 0.03 | 0.47 | 0.27 | 0.27 | 0.25 | 0.25 |
| **homova-braycurtis** | | | | | | |
| HOMOVA | BValue | P-value | SSwithin/(Ni-1)_values | | | |
| | | | post1 | post2 | pre1 | pre2 |
| | 0.17 | 0.37 | 0.24 | 0.26 | 0.22 | 0.20 |
| **pre1-pre2-post1-post2 HDP 3** | | | | | | |
| **homova-thetayc** | | | | | | |
| HOMOVA | BValue | P-value | SSwithin/(Ni-1)_values | | | |
| | | | post1 | post2 | pre1 | pre2 |
| | 0.051 | 0.77 | 0.33 | 0.33 | 0.33 | 0.29 |
| **homova-jclass** | | | | | | |
| HOMOVA | BValue | P-value | SSwithin/(Ni-1)_values | | | |
| | | | post1 | post2 | pre1 | pre2 |
| | 0.01 | 0.91 | 0.27 | 0.26 | 0.25 | 0.25 |
| **homova-braycurtis** | | | | | | |
| HOMOVA | BValue | P-value | SSwithin/(Ni-1)_values | | | |
| | | | post1 | post2 | pre1 | pre2 |
| | 0.06 | 0.77 | 0.24 | 0.23 | 0.22 | 0.20 |

Notes: HDP, healthy dietary pattern.

Supplemental Table S5. Taxa that significantly differed between groups based on LEfSe analyses.

| Group 1 vs Group 2 | Domain | Phylum | Class | Order | Family | Genus | Level | Higher | LDA | Phylotype-OTU | Mean 1 | Var 1 | SD 1 | Mean 2 | Var 2 | SD 2 | Relative abundance (RA, %) | >=0.1% RA |
|---|---|---|---|---|---|---|---|---|---|---|---|---|---|---|---|---|---|---|
| All HDPs, Pre vs Post | Bacteria | Firmicutes | Clostridia | Clostridiales | Lachnospiraceae | Dorea | genus | pre | 3.7 | OTU013 | 0.027945 | 0.000323 | 0.001926 | 0.01757 | 0.000286 | 0.001802 | 2.27278173 | Yes |
| | Bacteria | Firmicutes | Clostridia | Clostridiales | Lachnospiraceae | Mediterraneibacter | genus | pre | 3.7 | OTU018 | 0.019352 | 0.000374 | 0.002073 | 0.009246 | 0.000129 | 0.001212 | 1.42702255 | Yes |
| | Bacteria | Firmicutes | Clostridia | Clostridiales | Lachnospiraceae | Coprococcus | genus | post | 3.3 | OTU021 | 0.010025 | 0.000112 | 0.001132 | 0.014385 | 0.000182 | 0.00144 | 1.22177549 | Yes |
| | Bacteria | Actinobacteria | Coriobacteriia | Coriobacteriales | Coriobacteriaceae | Collinsella | genus | pre | 3.5 | OTU024 | 0.014693 | 0.000288 | 0.00182 | 0.00818 | 0.00013 | 0.001216 | 1.14177111 | Yes |
| | Bacteria | Firmicutes | Clostridia | Clostridiales | Ruminococcaceae | Ruminococcus | genus | post | 3.3 | OTU026 | 0.008302 | 0.000053 | 0.000778 | 0.012093 | 0.000106 | 0.001096 | 1.02085338 | Yes |
| | Bacteria | Firmicutes | Clostridia | Clostridiales | Peptostreptococcaceae | Romboutsia | genus | pre | 3.5 | OTU030 | 0.009972 | 0.000248 | 0.001687 | 0.004204 | 0.000035 | 0.000627 | 0.70716327 | Yes |
| | Bacteria | Firmicutes | Clostridia | Clostridiales | Lachnospiraceae | Lachnospira | genus | post | 2.4 | OTU038 | 0.00184 | 0.000031 | 0.000594 | 0.002025 | 0.000011 | 0.000356 | 0.1933062 | Yes |
| | Bacteria | Firmicutes | Erysipelotrichia | Erysipelotrichales | Erysipelotrichaceae | Turicibacter | genus | pre | 2.8 | OTU054 | 0.002229 | 0.000072 | 0.000908 | 0.001012 | 0.00001 | 0.000336 | 0.16173215 | Yes |
| Post, HDP 2 vs HDP 1 | Bacteria | Firmicutes | Clostridia | Clostridiales | Lachnospiraceae | Anaerostipes | genus | Post HDP1 | 3.8 | OTU011 | 0.01785 | 0.000221 | 0.002668 | 0.030643 | 0.000743 | 0.004675 | 2.94548793 | Yes |
| Post, HDP 3 vs HDP 1 | Bacteria | Bacteroidetes | Bacteroidia | Bacteroidales | Porphyromonadaceae | Parabacteroides | genus | Post HDP1 | 3.2 | OTU016 | 0.003975 | 0.00003 | 0.001136 | 0.006409 | 0.000025 | 0.000862 | 0.59857727 | Yes |
| | Bacteria | Firmicutes | Clostridia | Clostridiales | Ruminococcaceae | Clostridium_IV | genus | Post HDP1 | 2.8 | OTU049 | 0.0004 | 0 | 0.000107 | 0.001548 | 0.000014 | 0.000637 | 0.17888799 | Yes |

Supplemental Table S5. continued

| Group 1 vs Group 2 | Domain | Phylum | Class | Order | Family | Genus | Level | Higher | LDA | Phylotype-OTU | Mean 1 | Var 1 | SD 1 | Mean 2 | Var 2 | SD 2 | Relative abundance (RA, %) | >=0.1% RA |
|---|---|---|---|---|---|---|---|---|---|---|---|---|---|---|---|---|---|---|
| Post, HDP 3 vs HDP 2 | Bacteria | Firmicutes | Clostridia | Clostridiales | Lachnospiraceae | Dorea | genus | postHDP3 | 3.8 | OTU013 | 0.026484 | 0.000709 | 0.005552 | 0.014053 | 0.000118 | 0.001955 | 2.27278173 | Yes |
| | Bacteria | Bacteroidetes | Bacteroidia | Bacteroidales | Porphyromonadaceae | Parabacteroides | genus | postHDP2 | 3.3 | OTU016 | 0.003975 | 0.00003 | 0.001136 | 0.009112 | 0.000266 | 0.002929 | 0.59857727 | Yes |
| | Bacteria | Bacteria _unclassified | Bacteria _unclassified | Bacteria _unclassified | Bacteria_unclassified | Bacteria _unclassified | genus | postHDP2 | 3.7 | OTU020 | 0.003422 | 0.000036 | 0.001254 | 0.012616 | 0.000307 | 0.003149 | 0.68580683 | Yes |
| Pre, HDP 2 vs HDP 1 | Bacteria | Firmicutes | Clostridia | Clostridiales | Lachnospiraceae | Roseburia | genus | preHDP2 | 3.6 | OTU014 | 0.017758 | 0.000331 | 0.003214 | 0.010443 | 0.000103 | 0.001767 | 1.50285885 | Yes |
| Pre, HDP 3 vs HDP 1 | Bacteria | Firmicutes | Clostridia | Clostridiales | Clostridiales_unclassified | Clostridiales _unclassified | genus | preHDP1 | 3.7 | OTU009 | 0.00935 | 0.000171 | 0.002791 | 0.018953 | 0.000518 | 0.00396 | 1.52627814 | Yes |
| | Bacteria | Bacteria _unclassified | Bacteria _unclassified | Bacteria _unclassified | Bacteria_unclassified | Bacteria _unclassified | genus | preHDP1 | 3.3 | OTU020 | 0.002228 | 0.00002 | 0.000944 | 0.006265 | 0.000108 | 0.001809 | 0.68580683 | Yes |
| Pre, HDP 3 vs HDP 2 | Bacteria | Firmicutes | Bacilli | Lactobacillales | Streptococcaceae | Streptococcus | genus | preHDP3 | 3.7 | OTU023 | 0.017785 | 0.00147 | 0.008173 | 0.005924 | 0.00008 | 0.001585 | 0.84383763 | Yes |
| HDP1, Pre vs Post | Bacteria | Firmicutes | Clostridia | Clostridiales | Lachnospiraceae | Dorea | genus | preHDP1 | 3.8 | OTU013 | 0.027756 | 0.000336 | 0.003189 | 0.014746 | 0.000098 | 0.001695 | 2.27278173 | Yes |
| | Bacteria | Firmicutes | Clostridia | Clostridiales | Lachnospiraceae | Mediterraneibacter | genus | preHDP1 | 3.8 | OTU018 | 0.019151 | 0.000243 | 0.002713 | 0.00751 | 0.00007 | 0.001438 | 1.42702255 | Yes |
| | Bacteria | Actinobacteria | Coriobacteriia | Coriobacteriales | Coriobacteriaceae | Collinsella | genus | preHDP1 | 3.6 | OTU024 | 0.017478 | 0.000436 | 0.003633 | 0.009381 | 0.000263 | 0.002783 | 1.14177111 | Yes |
| | Bacteria | Firmicutes | Clostridia | Clostridiales | Peptostreptococcaceae | Romboutsia | genus | preHDP1 | 3.7 | OTU030 | 0.011777 | 0.000514 | 0.003948 | 0.003264 | 0.00002 | 0.000766 | 0.70716327 | Yes |
| | Bacteria | Firmicutes | Clostridia | Clostridiales | Lachnospiraceae | Lachnospira | genus | preHDP1 | 2.7 | OTU038 | 0.002554 | 0.00007 | 0.001452 | 0.002037 | 0.000009 | 0.000517 | 0.1933062 | Yes |
| HDP2, Pre vs Post | Bacteria | Firmicutes | Clostridia | Clostridiales | Lachnospiraceae | Anaerostipes | genus | preHDP2 | 3.8 | OTU011 | 0.030977 | 0.000919 | 0.005358 | 0.01785 | 0.000221 | 0.002668 | 2.94548793 | Yes |
| | Bacteria | Firmicutes | Clostridia | Clostridiales | Lachnospiraceae | Dorea | genus | preHDP2 | 3.8 | OTU013 | 0.026183 | 0.000236 | 0.002716 | 0.014053 | 0.000118 | 0.001955 | 2.27278173 | Yes |
| | Bacteria | Firmicutes | Clostridia | Clostridiales | Lachnospiraceae | Mediterraneibacter | genus | preHDP2 | 3.6 | OTU018 | 0.016617 | 0.000185 | 0.002406 | 0.007421 | 0.000043 | 0.001174 | 1.42702255 | Yes |
| | Bacteria | Actinobacteriac | Coriobacteriia | Coriobacteriales | Coriobacteriaceae | Collinsella | genus | preHDP2 | 3.7 | OTU024 | 0.014546 | 0.000302 | 0.003074 | 0.007244 | 0.000041 | 0.001147 | 1.14177111 | Yes |
| | Bacteria | Firmicutes | Clostridia | Clostridiales | Peptostreptococcaceae | Romboutsia | genus | preHDP2 | 3.3 | OTU030 | 0.008262 | 0.000078 | 0.001565 | 0.004709 | 0.000055 | 0.001335 | 0.70716327 | Yes |
| HDP3, Pre vs Post | Bacteria | Actinobacteria | Coriobacteriia | Coriobacteriales | Coriobacteriaceae | Collinsella | genus | preHDP3 | 3.2 | OTU024 | 0.010729 | 0.000042 | 0.001374 | 0.007665 | 0.00006 | 0.001616 | 1.14177111 | Yes |
| | Bacteria | Firmicutes | Clostridia | Clostridiales | Lachnospiraceae | Lachnospira | genus | postHDP3 | 2.4 | OTU038 | 0.000907 | 0.000004 | 0.000427 | 0.00128 | 0.000003 | 0.000354 | 0.1933062 | Yes |
| | Bacteria | Firmicutes | Erysipelotrichia | Erysipelotrichales | Erysipelotrichaceae | Turicibacter | genus | preHDP3 | 2.8 | OTU054 | 0.002219 | 0.000007 | 0.000557 | 0.000745 | 0.000002 | 0.000313 | 0.16173215 | Yes |

Supplemental Table S5. continued

| Group 1 vs Group 2 | Domain | Phylum | Class | Order | Family | Genus | Level | Higher | LDA | Phylotype-OTU | Mean 1 | Var 1 | SD 1 | Mean 2 | Var 2 | SD 2 | Relative abundance (RA, %) | >= 0.1 % RA |
|---|---|---|---|---|---|---|---|---|---|---|---|---|---|---|---|---|---|---|
| Washout 1, pre-HDP 2 vs post-HDP 1 | Bacteria | Firmicutes | Clostridia | Clostridiales | Lachnospiraceae | Dorea | genus | preHDP2 | 3.8 | OTU013 | 0.026183 | 0.000236 | 0.002716 | 0.014746 | 0.000098 | 0.001695 | 2.27278173 | Yes |
| | Bacteria | Firmicutes | Clostridia | Clostridiales | Lachnospiraceae | Mediterraneibacter | genus | preHDP2 | 3.7 | OTU018 | 0.016617 | 0.000185 | 0.002406 | 0.00751 | 0.00007 | 0.001438 | 1.42702255 | Yes |
| | Bacteria | Actinobacteria | Coriobacteriia | Coriobacteriales | Coriobacteriaceae | Collinsella | genus | preHDP2 | 3.5 | OTU024 | 0.014546 | 0.000302 | 0.003074 | 0.009381 | 0.000263 | 0.002783 | 1.14177111 | Yes |
| | Bacteria | Firmicutes | Clostridia | Clostridiales | Peptostreptococcaceae | Romboutsia | genus | preHDP2 | 3.4 | OTU030 | 0.008262 | 0.000078 | 0.001565 | 0.003264 | 0.00002 | 0.000766 | 0.70716327 | Yes |
| Washout 2, pre-HDP 3 vs post-HDP 2 | Bacteria | Firmicutes | Clostridia | Clostridiales | Lachnospiraceae | Blautia | genus | preHDP3 | 4.5 | OTU003 | 0.134056 | 0.00862 | 0.019794 | 0.082726 | 0.003272 | 0.010274 | 0.70716327 | Yes |
| | Bacteria | Firmicutes | Clostridia | Clostridiales | Clostridiales _unclassified | Clostridiales _unclassified | genus | postHDP2 | 3.7 | OTU009 | 0.00935 | 0.000171 | 0.002791 | 0.018604 | 0.000405 | 0.003615 | 1.52627814 | Yes |
| | Bacteria | Firmicutes | Clostridia | Clostridiales | Lachnospiraceae | Dorea | genus | preHDP3 | 4.0 | OTU013 | 0.030791 | 0.000449 | 0.004517 | 0.014053 | 0.000118 | 0.001955 | 2.27278173 | Yes |
| | Bacteria | Bacteroidetes | Bacteroidia | Bacteroidales | Porphyromonadaceae | Parabacteroides | genus | postHDP2 | 3.4 | OTU016 | 0.003577 | 0.000047 | 0.001463 | 0.009112 | 0.000266 | 0.002929 | 0.59857727 | Yes |
| | Bacteria | Firmicutes | Clostridia | Clostridiales | Lachnospiraceae | Mediterraneibacter | genus | preHDP3 | 3.9 | OTU018 | 0.023632 | 0.000857 | 0.006243 | 0.007421 | 0.000043 | 0.001174 | 1.42702255 | Yes |
| | Bacteria | Bacteria _unclassified | Bacteria _unclassified | Bacteria _unclassified | Bacteria _unclassified | Bacteria _unclassified | genus | postHDP2 | 3.8 | OTU020 | 0.002228 | 0.00002 | 0.000944 | 0.012616 | 0.000307 | 0.003149 | 0.68580683 | Yes |
| | Bacteria | Firmicutes | Bacilli | Lactobacillales | Streptococcaceae | Streptococcus | genus | preHDP3 | 3.7 | OTU023 | 0.017785 | 0.00147 | 0.008173 | 0.009235 | 0.000424 | 0.003697 | 0.84383763 | Yes |
| | Bacteria | Actinobacteria | Coriobacteriia | Coriobacteriales | Coriobacteriaceae | Collinsella | genus | preHDP3 | 3.2 | OTU024 | 0.010729 | 0.000042 | 0.001374 | 0.007244 | 0.000041 | 0.001147 | 1.14177111 | Yes |
| | Bacteria | Firmicutes | Clostridia | Clostridiales | Peptostreptococcaceae | Romboutsia | genus | preHDP3 | 3.3 | OTU030 | 0.009751 | 0.000105 | 0.00218 | 0.004709 | 0.000055 | 0.001335 | 0.70716327 | Yes |
| | Bacteria | Firmicutes | Erysipelotrichia | Erysipelotrichales | Erysipelotrichaceae | Faecalibacillus | genus | preHDP3 | 3.6 | OTU035 | 0.011849 | 0.000642 | 0.0054 | 0.003052 | 0.000018 | 0.000763 | 0.63864068 | Yes |
| | Bacteria | Firmicutes | Clostridia | Clostridiales | Lachnospiraceae | Lachnospira | genus | postHDP2 | 3.0 | OTU038 | 0.000907 | 0.000004 | 0.000427 | 0.002564 | 0.00002 | 0.000794 | 0.1933062 | Yes |
| | Bacteria | Firmicutes | Erysipelotrichia | Erysipelotrichales | Erysipelotrichaceae | Turicibacter | genus | preHDP3 | 3.0 | OTU054 | 0.002219 | 0.000007 | 0.000557 | 0.000436 | 0.000001 | 0.000142 | 0.16173215 | Yes |
| | Bacteria | Bacteroidetes | Bacteroidetes _unclassified | Bacteroidetes _unclassified | Bacteroidetes _unclassified | Bacteroidetes _unclassified | genus | postHDP2 | 3.0 | OTU065 | 0.00184 | 0.000065 | 0.001719 | 0.003136 | 0.000094 | 0.00174 | 0.19673812 | Yes |
| Washout, pre-HDPs 2&3 vs post-HDPs 1&2 | Bacteria | Firmicutes | Clostridia | Clostridiales | Lachnospiraceae | Blautia | genus | pre | 4.3 | OTU003 | 0.119567 | 0.006124 | 0.010649 | 0.08421 | 0.002528 | 0.006236 | 0.70716327 | Yes |
| | Bacteria | Firmicutes | Clostridia | Clostridiales | Lachnospiraceae | Dorea | genus | pre | 3.8 | OTU013 | 0.028061 | 0.000321 | 0.002438 | 0.014415 | 0.000106 | 0.001277 | 2.27278173 | Yes |
| | Bacteria | Bacteroidetes | Bacteroidia | Bacteroidales | Porphyromonadaceae | Parabacteroides | genus | post | 3.4 | OTU016 | 0.004097 | 0.000032 | 0.000775 | 0.007698 | 0.00014 | 0.001465 | 0.59857727 | Yes |
| | Bacteria | Firmicutes | Clostridia | Clostridiales | Lachnospiraceae | Mediterraneibacter | genus | pre | 3.8 | OTU018 | 0.019475 | 0.00046 | 0.002919 | 0.007468 | 0.000056 | 0.000931 | 1.42702255 | Yes |
| | Bacteria | Firmicutes | Clostridia | Clostridiales | Lachnospiraceae | Coprococcus | genus | post | 3.5 | OTU021 | 0.009395 | 0.00012 | 0.001489 | 0.014872 | 0.000201 | 0.001758 | 1.22177549 | Yes |

## Supplemental Table S5. continued

| Group 1 vs Group 2 | Domain | Phylum | Class | Order | Family | Genus | Level | Higher | LDA | Phylotype-OTU | Mean 1 | Var 1 | SD 1 | Mean 2 | Var 2 | SD 2 | Relative abundance (RA, %) | >=0.1% RA |
|---|---|---|---|---|---|---|---|---|---|---|---|---|---|---|---|---|---|---|
| Washout, pre-HDPs 2&3 vs post-HDPs 1&2 (cont) | Bacteria | Actinobacteria | Coriobacteriia | Coriobacteriales | Coriobacteriaceae | Collinsella | genus | pre | 3.4 | OTU024 | 0.012991 | 0.000197 | 0.00191 | 0.008362 | 0.000156 | 0.001549 | 1.14177111 | Yes |
| | Bacteria | Firmicutes | Clostridia | Clostridiales | Peptostreptococcaceae | Romboutsia | genus | pre | 3.4 | OTU030 | 0.008869 | 0.000088 | 0.001275 | 0.003953 | 0.000037 | 0.000752 | 0.70716327 | Yes |
| | Bacteria | Verrucomicrobia | Verrucomicrobiae | Verrucomicrobiales | Akkermansiaceae | Akkermansia | genus | post | 3.6 | OTU032 | 0.013403 | 0.000983 | 0.004267 | 0.020282 | 0.001038 | 0.003996 | 1.70822572 | Yes |
| | Bacteria | Firmicutes | Clostridia | Clostridiales | Lachnospiraceae | Lachnospira | genus | post | 2.6 | OTU038 | 0.001404 | 0.000007 | 0.000366 | 0.002288 | 0.000014 | 0.000463 | 0.1933062 | Yes |
| | Bacteria | Firmicutes | Erysipelotrichia | Erysipelotrichales | Erysipelotrichaceae | Turicibacter | genus | pre | 2.5 | OTU054 | 0.001664 | 0.000006 | 0.000323 | 0.001107 | 0.000013 | 0.000443 | 0.16173215 | Yes |
| All HDPs, Pre vs Post | Bacteria | Actinobacteria | Coriobacteriia | Eggerthellales | Eggerthellaceae | Adlercreutzia | genus | pre | 2.4 | OTU033 | 0.00093 | 0.000001 | 0.000129 | 0.000478 | 0 | 0.000075 | 0.07028832 | No |
| | Bacteria | Firmicutes | Clostridia | Clostridiales | Lachnospiraceae | Ruminococcus2 | genus | pre | 2.3 | OTU061 | 0.001052 | 0.000002 | 0.000152 | 0.000722 | 0.000003 | 0.000169 | 0.08860291 | No |
| | Bacteria | Firmicutes | Clostridia | Clostridiales | Ruminococcaceae | Dysosmobacter | genus | post | 2.2 | OTU069 | 0.000315 | 0 | 0.000058 | 0.000468 | 0 | 0.000072 | 0.03918102 | No |
| | Bacteria | Proteobacteria | Gammaproteobacteria | Pasteurellales | Pasteurellaceae | Pasteurellaceae_unclassified | genus | pre | 2.5 | OTU097 | 0.000308 | 0.000008 | 0.0003 | 0.00003 | 0 | 0.000012 | 0.01682465 | No |
| | Bacteria | Firmicutes | Erysipelotrichia | Erysipelotrichales | Erysipelotrichaceae | Massilimicrobiota | genus | pre | 2.2 | OTU125 | 0.000105 | 0 | 0.000029 | 0.00002 | 0 | 0.000006 | 0.0062296 | No |
| | Bacteria | Proteobacteria | Betaproteobacteria | Burkholderiales | Burkholderiaceae | Paraburkholderia | genus | pre | 2.5 | OTU131 | 0.000059 | 0 | 0.000017 | 0.000008 | 0 | 0.000003 | 0.00334624 | No |
| | Bacteria | Firmicutes | Bacilli | Lactobacillales | Lactobacillaceae | Lacticaseibacillus | genus | pre | 2.3 | OTU138 | 0.000343 | 0.000003 | 0.000177 | 0.000033 | 0 | 0.000029 | 0.01870313 | No |
| | Bacteria | Firmicutes | Bacilli | Bacillales | Bacillales_Incertae_Sedis_XI | Gemella | genus | pre | 2.9 | OTU139 | 0.000035 | 0 | 0.000011 | 0.000006 | 0 | 0.000002 | 0.00201855 | No |
| | Bacteria | Firmicutes | Bacilli | Lactobacillales | Lactobacillaceae | Latilactobacillus | genus | pre | 2.8 | OTU171 | 0.000018 | 0 | 0.000007 | 0.000002 | 0 | 0.000001 | 0.00098026 | No |
| Post, HDP 2 vs HDP 1 | Bacteria | Firmicutes | Bacilli | Lactobacillales | Lactobacillales_unclassified | Lactobacillales_unclassified | genus | Post HDP1 | 3.5 | OTU153 | 0.000005 | 0 | 0.000003 | 0.000023 | 0 | 0.000007 | 0.00197116 | No |
| Post, HDP 3 vs HDP 1 | Bacteria | Bacteroidetes | Bacteroidia | Bacteroidales | Odoribacteraceae | Butyricimonas | genus | Post HDP1 | 2.5 | OTU042 | 0.000278 | 0 | 0.000128 | 0.000943 | 0.000003 | 0.000287 | 0.07089194 | No |
| | Bacteria | Firmicutes | Clostridia | Clostridia_unclassified | Clostridia_unclassified | Clostridia_unclassified | genus | Post HDP1 | 2.3 | OTU050 | 0.000224 | 0 | 0.000068 | 0.000818 | 0.000003 | 0.000276 | 0.06304304 | No |
| Post, HDP 3 vs HDP 2 | Bacteria | Bacteroidetes | Bacteroidia | Bacteroidales | Odoribacteraceae | Butyricimonas | genus | Post HDP2 | 2.2 | OTU042 | 0.000278 | 0 | 0.000128 | 0.000548 | 0.000001 | 0.000135 | 0.07089194 | No |
| | Bacteria | Firmicutes | Clostridia | Clostridia ,_unclassified | Clostridia_unclassified | Clostridia_unclassified | genus | Post HDP2 | 2.6 | OTU050 | 0.000224 | 0 | 0.000068 | 0.000958 | 0.000004 | 0.000361 | 0.06304304 | No |
| Pre, HDP 2 vs HDP 1 | Bacteria | Actinobacteria | Actinobacteria | Micrococcales | Micrococcaceae | Rothia | genus | Pre HDP2 | 3.2 | OTU124 | 0.000016 | 0 | 0.000006 | 0.000001 | 0 | 0.000001 | 0.00093097 | No |
| | Bacteria | Firmicutes | Bacilli | Lactobacillales | Lactobacillaceae | Lacticaseibacillus | genus | Pre HDP1 | 2.3 | OTU138 | 0.000066 | 0 | 0.000059 | 0.000585 | 0.000006 | 0.000422 | 0.01870313 | No |
| Pre, HDP 3 vs HDP 1 | Bacteria | Proteobacteria | Gammaproteobacteria | Enterobacterales | Enterobacteriaceae | Klebsiella | genus | Pre HDP1 | 2.8 | OTU208 | 0.000207 | 0 | 0.000125 | 0.001562 | 0.000079 | 0.001546 | 0.03708964 | No |
| Pre, HDP 3 vs HDP 2 | Bacteria | Bacteroidetes | Bacteroidia | Bacteroidales | Odoribacteraceae | Butyricimonas | genus | Pre HDP2 | 2.4 | OTU042 | 0.000301 | 0 | 0.000123 | 0.000794 | 0.000001 | 0.000166 | 0.07089194 | No |
| | Bacteria | Firmicutes | Bacilli | Lactobacillales | Lactobacillaceae | Lacticaseibacillus | genus | Pre HDP3 | 2.1 | OTU138 | 0.000382 | 0.000002 | 0.000288 | 0.000066 | 0 | 0.000059 | 0.01870313 | No |

# Supplemental Table S5. continued

| Group 1 vs Group 2 | Domain | Phylum | Class | Order | Family | Genus | Level | Higher | LDA | Phylotype-OTU | Mean 1 | Var 1 | SD 1 | Mean 2 | Var 2 | SD 2 | Relative abundance (RA, %) | >=0.1% RA |
|---|---|---|---|---|---|---|---|---|---|---|---|---|---|---|---|---|---|---|
| HDP1, Pre vs Post | Bacteria | Firmicutes | Bacilli | Lactobacillales | Lactobacillaceae | Lacticaseibacillus | genus | preHDP1 | 2.3 | OTU138 | 0.000585 | 0.000006 | 0.000422 | 0.000083 | 0 | 0.000076 | 0.01870313 | No |
| | Bacteria | Actinobacteria | Coriobacteriia | Eggerthellales | Eggerthellaceae | Adlercreutzia | genus | preHDP2 | 3.1 | OTU033 | 0.000836 | 0.000001 | 0.000213 | 0.000373 | 0.000001 | 0.000131 | 0.07028832 | No |
| | Bacteria | Firmicutes | Clostridia | Clostridiales | Lachnospiraceae | Ruminococcus2 | genus | preHDP2 | 3.2 | OTU061 | 0.001153 | 0.000003 | 0.000292 | 0.000615 | 0.000004 | 0.000362 | 0.08860291 | No |
| HDP2, Pre vs Post | Bacteria | Firmicutes | Bacilli | Lactobacillales | Lactobacillaceae | Lactobacillus | genus | postHDP2 | 4.1 | OTU089 | 0.000028 | 0 | 0.000008 | 0.000194 | 0 | 0.00007 | 0.021098 | No |
| | Bacteria | Firmicutes | Bacilli | Lactobacillales | Lactobacillaceae | Lacticaseibacillus | genus | preHDP3 | 2.3 | OTU138 | 0.000382 | 0.000002 | 0.000288 | 0.000002 | 0 | 0.000001 | 0.01870313 | No |
| | Bacteria | Fusobacteria | Fusobacteriia | Fusobacteriales | Leptotrichiaceae | Leptotrichiaceae_unclassified | genus | preHDP3 | 2.2 | OTU203 | 0.000045 | 0 | 0.00003 | 0 | 0 | 0 | 0.0005791 | No |
| HDP3, Pre vs Post | Bacteria | Proteobacteria | Gammaproteobacteria | Enterobacterales | Enterobacteriaceae | Klebsiella | genus | preHDP3 | 2.0 | OTU208 | 0.000207 | 0 | 0.000125 | 0.000005 | 0 | 0.000005 | 0.03708964 | No |
| | Bacteria | Firmicutes | Bacilli | Lactobacillales | Lactobacillaceae | Lactobacillus | genus | postHDP1 | 3.1 | OTU089 | 0.000028 | 0 | 0.000008 | 0.000143 | 0 | 0.000032 | 0.021098 | No |
| | Bacteria | Firmicutes | Bacilli | Lactobacillales | Lactobacillaceae | Ligilactobacillus | genus | postHDP1 | 2.5 | OTU110 | 0.000003 | 0 | 0.000002 | 0.000604 | 0.000005 | 0.000383 | 0.05015192 | No |
| | Bacteria | Firmicutes | Bacilli | Lactobacillales | Lactobacillales_unclassified | Lactobacillales_unclassified | genus | postHDP1 | 4.4 | OTU153 | 0.000009 | 0 | 0.000005 | 0.000023 | 0 | 0.000007 | 0.00197116 | No |
| | Bacteria | Firmicutes | Bacilli | Lactobacillales | Lactobacillaceae | Latilactobacillus | genus | preHDP2 | 3.3 | OTU171 | 0.000023 | 0 | 0.000017 | 0 | 0 | 0 | 0.00098026 | No |
| Washout 1, pre-HDP 2 vs post-HDP 1 | Bacteria | Proteobacteria | Gammaproteobacteria | Enterobacterales | Enterobacteriaceae | Klebsiella | genus | postHDP1 | 2.5 | OTU208 | 0.000041 | 0 | 0.000024 | 0.000166 | 0 | 0.000071 | 0.03708964 | No |
| | Bacteria | Actinobacteria | Coriobacteriia | Eggerthellales | Eggerthellaceae | Adlercreutzia | genus | preHDP3 | 2.9 | OTU033 | 0.001132 | 0.000002 | 0.000278 | 0.000373 | 0.000001 | 0.000131 | 0.07028832 | No |
| | Bacteria | Firmicutes | Clostridia | Clostridiales | Ruminococcaceae | Oscillibacter | genus | postHDP2 | 2.9 | OTU056 | 0.000083 | 0 | 0.000034 | 0.000254 | 0 | 0.000066 | 0.01553742 | No |
| | Bacteria | Firmicutes | Clostridia | Clostridiales | Lachnospiraceae | Ruminococcus2 | genus | preHDP3 | 2.9 | OTU061 | 0.001273 | 0.000003 | 0.000359 | 0.000615 | 0.000004 | 0.000362 | 0.08860291 | No |
| | Bacteria | Bacteroidetes | Bacteroidia | Bacteroidales | Barnesiellaceae | Coprobacter | genus | postHDP2 | 2.9 | OTU092 | 0.000051 | 0 | 0.000031 | 0.000134 | 0 | 0.000049 | 0.01411601 | No |
| | Bacteria | Lentisphaerae | Lentisphaeria | Victivallales | Victivallaceae | Victivallis | genus | postHDP2 | 3.4 | OTU100 | 0.000013 | 0 | 0.000011 | 0.000089 | 0 | 0.000031 | 0.00617798 | No |
| | Bacteria | Firmicutes | Clostridia | Clostridiales | Eubacteriaceae | Anaerofustis | genus | preHDP3 | 3.5 | OTU115 | 0.000067 | 0 | 0.000032 | 0.000009 | 0 | 0.000004 | 0.00329076 | No |
| | Bacteria | Firmicutes | Erysipelotrichia | Erysipelotrichales | Erysipelotrichaceae | Massilimicrobiota | genus | preHDP3 | 3.3 | OTU125 | 0.000127 | 0 | 0.000073 | 0.000022 | 0 | 0.000012 | 0.0062296 | No |
| | Bacteria | Firmicutes | Bacilli | Lactobacillales | Lactobacillaceae | Lacticaseibacillus | genus | preHDP3 | 2.9 | OTU138 | 0.000382 | 0.000002 | 0.000288 | 0.000001 | 0 | 0.000001 | 0.01870313 | No |
| | Bacteria | Firmicutes | Bacilli | Bacillales | Bacillales_Incertae_Sedis_XI | Gemella | genus | preHDP3 | 3.1 | OTU139 | 0.000081 | 0 | 0.00004 | 0.000002 | 0 | 0.000002 | 0.00201855 | No |
| | Bacteria | Firmicutes | Bacilli | Lactobacillales | Lactobacillales_unclassified | Lactobacillales_unclassified | genus | preHDP3 | 3.4 | OTU153 | 0.00006 | 0 | 0.000025 | 0.000005 | 0 | 0.000003 | 0.00197116 | No |
| | Bacteria | Fusobacteria | Fusobacteriia | Fusobacteriales | Leptotrichiaceae | Leptotrichiaceae_unclassified | genus | preHDP3 | 3.4 | OTU203 | 0.000045 | 0 | 0.00003 | 0 | 0 | 0 | 0.0005791 | No |
| Washout 2, pre-HDP 3 vs post-HDP 2 | Bacteria | Proteobacteria | Gammaproteobacteria | Enterobacterales | Enterobacteriaceae | Klebsiella | genus | preHDP3 | 2.8 | OTU208 | 0.000207 | 0 | 0.000125 | 0.000056 | 0 | 0.000048 | 0.03708964 | No |

Supplemental Table S5. continued

| Group 1 vs Group 2 | Domain | Phylum | Class | Order | Family | Genus | Level | Higher | LDA | Phylotype-OTU | Mean 1 | Var 1 | SD 1 | Mean 2 | Var 2 | SD 2 | Relative abundance (RA, %) | >=0.1 % RA |
|---|---|---|---|---|---|---|---|---|---|---|---|---|---|---|---|---|---|---|
| Washout, pre-HDPs 2&3 vs post-HDPs 1&2 | Bacteria | Actinobacteria | Coriobacteriia | Eggerthellales | Eggerthellaceae | Adlercreutzia | genus | pre | 2.4 | OTU033 | 0.000957 | 0.000002 | 0.000169 | 0.000452 | 0.000001 | 0.000092 | 0.07028832 | No |
| | Bacteria | Firmicutes | Clostridia | Clostridiales | Ruminococcaceae | Oscillibacter | genus | post | 2.1 | OTU056 | 0.000135 | 0 | 0.000025 | 0.000238 | 0 | 0.000039 | 0.01553742 | No |
| | Bacteria | Firmicutes | Clostridia | Clostridiales | Lachnospiraceae | Ruminococcus2 | genus | pre | 2.5 | OTU061 | 0.001202 | 0.000003 | 0.000225 | 0.000643 | 0.000003 | 0.000208 | 0.08860291 | No |
| | Bacteria | Firmicutes | Clostridia | Clostridiales | Ruminococcaceae | Dysosmobacter | genus | post | 2.2 | OTU069 | 0.000322 | 0 | 0.000081 | 0.000527 | 0.000001 | 0.000093 | 0.03918102 | No |
| | Bacteria | Firmicutes | Bacilli | Lactobacillales | Lactobacillaceae | Lactobacillus | genus | post | 2.7 | OTU089 | 0.000038 | 0 | 0.000012 | 0.000167 | 0 | 0.000037 | 0.021098 | No |
| | Bacteria | Firmicutes | Bacilli | Lactobacillales | Streptococcaceae | Lactococcus | genus | pre | 2.1 | OTU090 | 0.000174 | 0 | 0.00005 | 0.000077 | 0 | 0.000035 | 0.01486223 | No |
| | Bacteria | Proteobacteria | Gammaproteobacteria | Pasteurellales | Pasteurellaceae | Pasteurellaceae_unclassified | genus | post | 2.3 | OTU097 | 0.000009 | 0 | 0.000005 | 0.000036 | 0 | 0.000017 | 0.01682465 | No |
| | Bacteria | Lentisphaerae | Lentisphaeria | Victivallales | Victivallaceae | Victivallis | genus | post | 3.7 | OTU100 | 0.00003 | 0 | 0.000011 | 0.00009 | 0 | 0.000026 | 0.00617798 | No |
| | Bacteria | Firmicutes | Erysipelotrichia | Erysipelotrichales | Erysipelotrichaceae | Massilimicrobiota | genus | pre | 2.2 | OTU125 | 0.000109 | 0 | 0.00004 | 0.000017 | 0 | 0.000006 | 0.0062296 | No |
| | Bacteria | Firmicutes | Bacilli | Bacillales | Bacillales_Incertae_Sedis_XI | Gemella | genus | pre | 3.8 | OTU139 | 0.000039 | 0 | 0.000017 | 0.000003 | 0 | 0.000001 | 0.00201855 | No |
| | Bacteria | Firmicutes | Clostridia | Clostridiales | Clostridiales_Incertae_Sedis_XIII | Mogibacterium | genus | pre | 3.1 | OTU146 | 0.000011 | 0 | 0.000004 | 0.000008 | 0 | 0.000005 | 0.00075656 | No |
| All HDPs, Pre vs Post | Bacteria | Firmicutes | Bacilli | Bacillales | | | | pre | 2.3 | | | | | | | | | |
| | Bacteria | Proteobacteria | Betaproteobacteria | Burkholderiales | Burkholderiaceae | | | pre | 2.6 | | | | | | | | | |
| | Bacteria | Actinobacteria | Coriobacteriia | Eggerthellales | | | | pre | 2.8 | | | | | | | | | |
| | Bacteria | Actinobacteria | Coriobacteriia | Eggerthellales | Eggerthellaceae | | | pre | 2.8 | | | | | | | | | |
| | Bacteria | Proteobacteria | Gammaproteobacteria | Enterobacterales | | | | pre | 2.9 | | | | | | | | | |
| | Bacteria | Firmicutes | Bacilli | Bacillales | Bacillales_Incertae_Sedis_XI | | | pre | 3.0 | | | | | | | | | |
| | Bacteria | Firmicutes | Clostridia | Clostridiales | Peptostreptococcaceae | | | pre | 3.5 | | | | | | | | | |
| | Bacteria | Actinobacteria | Coriobacteriia | Coriobacteriales | | | | pre | 3.6 | | | | | | | | | |
| | Bacteria | Actinobacteria | Coriobacteriia | Coriobacteriales | Coriobacteriaceae | | | pre | 3.6 | | | | | | | | | |
| | Bacteria | Actinobacteria | Coriobacteriia | | | | | pre | 3.6 | | | | | | | | | |
| | Bacteria | Firmicutes | Clostridia | Clostridiales | Lachnospiraceae | | | pre | 4.4 | | | | | | | | | |
| | Bacteria | Firmicutes | Clostridia | | | | | pre | 4.5 | | | | | | | | | |
| | Bacteria | Firmicutes | Clostridia | Clostridiales | | | | pre | 4.5 | | | | | | | | | |
| | Bacteria | Firmicutes | | | | | | pre | 4.5 | | | | | | | | | |
| | Bacteria | Bacteroidetes | Bacteroidia | Bacteroidales | | | | post | 4.6 | | | | | | | | | |
| | Bacteria | Bacteroidetes | Bacteroidia | | | | | post | 4.6 | | | | | | | | | |
| | Bacteria | Bacteroidetes | | | | | | post | 4.6 | | | | | | | | | |

Supplemental Table S5. continued

| Group 1 vs Group 2 | Domain | Phylum | Class | Order | Family | Genus | Level | Higher | LDA | Phylotype-OTU | Mean 1 | Var 1 | SD 1 | Mean 2 | Var 2 | SD 2 | Relative abundance (RA, %) | >=0.1% RA |
|---|---|---|---|---|---|---|---|---|---|---|---|---|---|---|---|---|---|---|
| post | NONE | | | | | | | | | | | | | | | | | |
| Post, HDP 2 vs HDP 1 | Bacteria | Firmicutes | Bacilli | Lactobacillales | Lactobacillales _unclassified | | | postHDP 1 | 3.5 | | | | | | | | | |
| Post, HDP 3 vs HDP 1 | Bacteria | Firmicutes | Clostridia | Clostridia_unclassified | Clostridia_unclassified | | | postHDP 1 | 2.3 | | | | | | | | | |
| | Bacteria | Firmicutes | Clostridia | Clostridia_unclassified | | | | postHDP 1 | 2.3 | | | | | | | | | |
| | Bacteria | Bacteroidetes | Bacteroidia | Bacteroidales | Odoribacteraceae | | | postHDP 1 | 2.8 | | | | | | | | | |
| | Bacteria | Bacteroidetes | Bacteroidia | Bacteroidales | Porphyromonadaceae | | | postHDP 1 | 3.2 | | | | | | | | | |
| Post, HDP 3 vs HDP 2 | Bacteria | Bacteria_unclassified | | | | | | postHDP 2 | 3.7 | | | | | | | | | |
| | Bacteria | Bacteria_unclassified | Bacteria_unclassified | Bacteria_unclassified | Bacteria_unclassified | | | postHDP 2 | 3.7 | | | | | | | | | |
| | Bacteria | Bacteria_unclassified | Bacteria_unclassified | Bacteria_unclassified | | | | postHDP 2 | 3.7 | | | | | | | | | |
| | Bacteria | Bacteria_unclassified | Bacteria_unclassified | | | | | postHDP 2 | 3.7 | | | | | | | | | |
| | Bacteria | Bacteroidetes | Bacteroidia | Bacteroidales | Porphyromonadaceae | | | postHDP 2 | 3.3 | | | | | | | | | |
| | Bacteria | Firmicutes | Clostridia | Clostridia_unclassified | Clostridia_unclassified | | | postHDP 2 | 2.6 | | | | | | | | | |
| | Bacteria | Firmicutes | Clostridia | Clostridia_unclassified | | | | postHDP 2 | 2.6 | | | | | | | | | |
| pre | NONE | | | | | | | | | | | | | | | | | |
| Pre, HDP 2 vs HDP 1 | Bacteria | Firmicutes | Bacilli | Lactobacillales | Lactobacillaceae | | | preHDP1 | 2.9 | | | | | | | | | |
| | Bacteria | Actinobacteria | Actinobacteria | Micrococcales | | | | preHDP2 | 2.9 | | | | | | | | | |
| | Bacteria | Actinobacteria | Actinobacteria | Micrococcales | Micrococcaceae | | | preHDP2 | 3.2 | | | | | | | | | |
| Pre, HDP 3 vs HDP 1 | Bacteria | Firmicutes | Clostridia | Clostridiales | Clostridiales_unclassified | | | preHDP1 | 3.7 | | | | | | | | | |
| | Bacteria | Bacteria_unclassified | | | | | | preHDP1 | 3.3 | | | | | | | | | |
| | Bacteria | Bacteria_unclassified | Bacteria_unclassified | Bacteria _unclassified | Bacteria_unclassified | | | preHDP1 | 3.3 | | | | | | | | | |
| | Bacteria | Bacteria_unclassified | Bacteria_unclassified | Bacteria _unclassified | | | | preHDP1 | 3.3 | | | | | | | | | |
| | Bacteria | Bacteria_unclassified | Bacteria_unclassified | | | | | preHDP1 | 3.3 | | | | | | | | | |
| Pre, HDP 3 vs HDP 2 | Bacteria | Firmicutes | Bacilli | Lactobacillales | | | | preHDP3 | 3.8 | | | | | | | | | |
| | Bacteria | Firmicutes | Bacilli | | | | | preHDP3 | 3.8 | | | | | | | | | |
| | Bacteria | Firmicutes | Bacilli | Lactobacillales | Streptococcaceae | | | preHDP3 | 3.7 | | | | | | | | | |

| Group 1 vs Group 2 | Domain | Phylum | Class | Order | Family | Genus | Level | Higher | LDA | Phylotype-OTU | Mean 1 | Var 1 | SD 1 | Mean 2 | Var 2 | SD 2 | Relative abundance (RA, %) | >=0.1% RA |
|---|---|---|---|---|---|---|---|---|---|---|---|---|---|---|---|---|---|---|
| | Bacteria | Firmicutes | | | | | | preHDP2 | 2.7 | | | | | | | | | |

Supplemental Table S5. continued

| Group 1 vs Group 2 | Domain | Phylum | Class | Order | Family | Genus | Level | Higher | LDA | Phylotype-OTU | Mean 1 | Var 1 | SD 1 | Mean 2 | Var 2 | SD 2 | Relative abundance (RA, %) | >=0.1% RA |
|---|---|---|---|---|---|---|---|---|---|---|---|---|---|---|---|---|---|---|
| | Bacteria | Actinobacteria | Coriobacteriia | Coriobacteriales | Coriobacteriaceae | | | preHDP1 | 3.6 | | | | | | | | | |
| | Bacteria | Actinobacteria | Coriobacteriia | Coriobacteriales | | | | preHDP1 | 3.6 | | | | | | | | | |
| | Bacteria | Actinobacteria | Coriobacteriia | | | | | preHDP1 | 3.7 | | | | | | | | | |
| HDP1, Pre vs Post | Bacteria | Firmicutes | Clostridia | Clostridiales | Peptostreptococcaceae | | | preHDP1 | 3.7 | | | | | | | | | |
| | Bacteria | Firmicutes | Clostridia | | | | | preHDP2 | 4.7 | | | | | | | | | |
| | Bacteria | Firmicutes | Clostridia | Clostridiales | | | | preHDP2 | 4.7 | | | | | | | | | |
| | Bacteria | Bacteroidetes | | | | | | postHDP2 | 4.7 | | | | | | | | | |
| | Bacteria | Bacteroidetes | Bacteroidia | Bacteroidales | | | | postHDP2 | 4.7 | | | | | | | | | |
| | Bacteria | Bacteroidetes | Bacteroidia | | | | | postHDP2 | 4.7 | | | | | | | | | |
| | Bacteria | Firmicutes | | | | | | preHDP2 | 4.7 | | | | | | | | | |
| | Bacteria | Actinobacteria | Coriobacteriia | | | | | preHDP2 | 3.7 | | | | | | | | | |
| | Bacteria | Firmicutes | Clostridia | Clostridiales | Peptostreptococcaceae | | | preHDP2 | 3.4 | | | | | | | | | |
| | Bacteria | Actinobacteria | Coriobacteriia | Coriobacteriales | Coriobacteriaceae | | | preHDP2 | 4.2 | | | | | | | | | |
| | Bacteria | Actinobacteria | Coriobacteriia | Coriobacteriales | | | | preHDP2 | 4.2 | | | | | | | | | |
| | Bacteria | Firmicutes | Clostridia | Clostridiales | Lachnospiraceae | | | preHDP2 | 4.6 | | | | | | | | | |
| HDP2, Pre vs Post | Bacteria | Firmicutes | Clostridia | Clostridiales | Ruminococcaceae | | | preHDP2 | 4.1 | | | | | | | | | |
| | Bacteria | Fusobacteria | Fusobacteriia | | | | | preHDP3 | 2.4 | | | | | | | | | |
| | Bacteria | Fusobacteria | | | | | | preHDP3 | 2.2 | | | | | | | | | |
| | Bacteria | Fusobacteria | Fusobacteriia | Fusobacteriales | | | | preHDP3 | 2.3 | | | | | | | | | |
| | Bacteria | Proteobacteria | Gammaproteobacteria | Enterobacterales | Enterobacteriaceae | | | preHDP3 | 2.3 | | | | | | | | | |
| | Bacteria | Proteobacteria | Gammaproteobacteria | Enterobacterales | | | | preHDP3 | 2.3 | | | | | | | | | |
| | Bacteria | Fusobacteria | Fusobacteriia | Fusobacteriales | Leptotrichiaceae | | | preHDP3 | 2.2 | | | | | | | | | |
| | Bacteria | Actinobacteria | Coriobacteriia | Coriobacteriales | Coriobacteriaceae | | | preHDP3 | 3.2 | | | | | | | | | |
| HDP3, Pre vs Post | Bacteria | Actinobacteria | Coriobacteriia | Coriobacteriales | | | | preHDP3 | 3.2 | | | | | | | | | |

Supplemental Table S5. continued

| Group 1 vs Group 2 | Domain | Phylum | Class | Order | Family | Genus | Level | Higher | LDA | Phylotype-OTU | Mean 1 | Var 1 | SD 1 | Mean 2 | Var 2 | SD 2 | Relative abundance (RA, %) | >=0.1 % RA |
|---|---|---|---|---|---|---|---|---|---|---|---|---|---|---|---|---|---|---|
| | Bacteria | Firmicutes | Clostridia | Clostridiales | Peptostreptococcaceae | | | preHDP2 | 3.4 | | | | | | | | | |
| | Bacteria | Bacteroidetes | | | | | | postHDP1 | 4.7 | | | | | | | | | |
| | Bacteria | Bacteroidetes | Bacteroidia | Bacteroidales | | | | postHDP1 | 4.7 | | | | | | | | | |
| | Bacteria | Bacteroidetes | Bacteroidia | | | | | postHDP1 | 4.7 | | | | | | | | | |
| | Bacteria | Firmicutes | Clostridia | | | | | preHDP2 | 4.7 | | | | | | | | | |
| | Bacteria | Firmicutes | Clostridia | Clostridiales | | | | preHDP2 | 4.7 | | | | | | | | | |
| | Bacteria | Firmicutes | | | | | | preHDP2 | 4.7 | | | | | | | | | |
| | Bacteria | Firmicutes | Bacilli | Lactobacillales | Lactobacillaceae | | | postHDP1 | 2.6 | | | | | | | | | |
| | Bacteria | Actinobacteria | Coriobacteriia | Coriobacteriales | Coriobacteriaceae | | | preHDP2 | 3.5 | | | | | | | | | |
| | Bacteria | Actinobacteria | Coriobacteriia | Coriobacteriales | | | | preHDP2 | 3.5 | | | | | | | | | |
| | Bacteria | Firmicutes | Bacilli | Lactobacillales | Lactobacillales_unclassified | | | postHDP1 | 4.4 | | | | | | | | | |
| | Bacteria | Firmicutes | Bacilli | Lactobacillales | Carnobacteriaceae | | | postHDP1 | 3.4 | | | | | | | | | |
| Washout 1, pre-HDP 2 vs post-HDP 1 | Bacteria | Actinobacteria | Coriobacteriia | | | | | preHDP2 | 3.5 | | | | | | | | | |
| | Bacteria | Firmicutes | Bacilli | Bacillales | | | | preHDP3 | 3.3 | | | | | | | | | |
| | Bacteria | Firmicutes | Bacilli | Lactobacillales | Lactobacillales_unclassified | | | preHDP3 | 3.4 | | | | | | | | | |
| | Bacteria | Firmicutes | Bacilli | Bacillales | Bacillales_Incertae_Sedis_XI | | | preHDP3 | 3.1 | | | | | | | | | |
| | Bacteria | Firmicutes | Bacilli | Lactobacillales | | | | preHDP3 | 3.7 | | | | | | | | | |
| | Bacteria | Firmicutes | Bacilli | | | | | preHDP3 | 3.7 | | | | | | | | | |
| | Bacteria | Firmicutes | Clostridia | | | | | preHDP3 | 4.8 | | | | | | | | | |
| | Bacteria | Firmicutes | Clostridia | Clostridiales | | | | preHDP3 | 4.8 | | | | | | | | | |
| | Bacteria | Bacteroidetes | Bacteroidia | Bacteroidales | Porphyromonadaceae | | | postHDP2 | 3.4 | | | | | | | | | |
| | Bacteria | Firmicutes | Clostridia | Clostridiales | Lachnospiraceae | | | preHDP3 | 4.8 | | | | | | | | | |
| | Bacteria | Bacteria_unclassified | | | | | | postHDP2 | 3.8 | | | | | | | | | |
| | Bacteria | Bacteria_unclassified | Bacteria_unclassified | Bacteria_unclassified | Bacteria_unclassified | | | postHDP2 | 3.8 | | | | | | | | | |
| | Bacteria | Bacteria_unclassified | Bacteria_unclassified | Bacteria_unclassified | | | | postHDP2 | 3.8 | | | | | | | | | |
| | Bacteria | Bacteria_unclassified | Bacteria_unclassified | | | | | postHDP2 | 3.8 | | | | | | | | | |
| | Bacteria | Firmicutes | Clostridia | Clostridiales | Clostridiales_unclassified | | | postHDP2 | 3.7 | | | | | | | | | |
| Washout 2, pre-HDP 3 vs post-HDP 2 | Bacteria | Lentisphaerae | Lentisphaeria | Victivallales | | | | postHDP2 | 3.5 | | | | | | | | | |

Supplemental Table S5. continued

| Group 1 vs Group 2 | Domain | Phylum | Class | Order | Family | Genus | Level | Higher | LDA | Phylotype-OTU | Mean 1 | Var 1 | SD 1 | Mean 2 | Var 2 | SD 2 | Relative (RA, %) | abundance | >=0.1% RA |
|---|---|---|---|---|---|---|---|---|---|---|---|---|---|---|---|---|---|---|---|
| Washout 2, pre-HDP 3 vs post-HDP 2 (cont) | Bacteria | Lentisphaerae | Lentisphaeria | | | | | postHDP2 | 3.3 | | | | | | | | | | |
| | Bacteria | Lentisphaerae | Lentisphaeria | Victivallales | Victivallaceae | | | postHDP2 | 3.4 | | | | | | | | | | |
| | Bacteria | Lentisphaerae | | | | | | postHDP2 | 3.3 | | | | | | | | | | |
| | Bacteria | Firmicutes | Bacilli | Lactobacillales | Streptococcaceae | | | preHDP3 | 3.7 | | | | | | | | | | |
| | Bacteria | Fusobacteria | Fusobacteriia | Fusobacteriales | Leptotrichiaceae | | | preHDP3 | 3.4 | | | | | | | | | | |
| | Bacteria | Firmicutes | | | | | | preHDP3 | 4.7 | | | | | | | | | | |
| | Bacteria | Bacteroidetes | Bacteroidetes_unclassified | | | | | postHDP2 | 3.0 | | | | | | | | | | |
| | Bacteria | Bacteroidetes | Bacteroidetes_unclassified | Bacteroidetes_unclassified | | | | postHDP2 | 3.0 | | | | | | | | | | |
| | Bacteria | Bacteroidetes | Bacteroidetes_unclassified | Bacteroidetes_unclassified | Bacteroidetes_unclassified | | | postHDP2 | 3.0 | | | | | | | | | | |
| | Bacteria | Actinobacteria | Coriobacteriia | Coriobacteriales | Coriobacteriaceae | | | preHDP3 | 3.2 | | | | | | | | | | |
| | Bacteria | Actinobacteria | Coriobacteriia | Coriobacteriales | | | | preHDP3 | 3.2 | | | | | | | | | | |
| | Bacteria | Actinobacteria | Coriobacteriia | | | | | preHDP3 | 3.5 | | | | | | | | | | |
| | Bacteria | Fusobacteria | Fusobacteriia | | | | | preHDP3 | 3.4 | | | | | | | | | | |
| | Bacteria | Fusobacteria | | | | | | preHDP3 | 3.3 | | | | | | | | | | |
| | Bacteria | Fusobacteria | Fusobacteriia | Fusobacteriales | | | | preHDP3 | 3.3 | | | | | | | | | | |
| | Bacteria | Actinobacteria | Actinobacteria | Actinomycetales | Actinomycetaceae | | | preHDP3 | 3.2 | | | | | | | | | | |
| | Bacteria | Actinobacteria | Actinobacteria | Actinomycetales | | | | preHDP3 | 3.2 | | | | | | | | | | |
| | Bacteria | Firmicutes | Clostridia | Clostridiales | Eubacteriaceae | | | preHDP3 | 3.6 | | | | | | | | | | |
| | Bacteria | Firmicutes | Clostridia | Clostridiales | Peptostreptococcaceae | | | preHDP3 | 3.4 | | | | | | | | | | |
| | Bacteria | Actinobacteria | Coriobacteriia | Eggerthellales | | | | preHDP3 | 3.1 | | | | | | | | | | |
| | Bacteria | Actinobacteria | Coriobacteriia | Eggerthellales | Eggerthellaceae | | | preHDP3 | 3.1 | | | | | | | | | | |
| | Bacteria | Bacteroidetes | Bacteroidia | Bacteroidales | | | | postHDP2 | 4.6 | | | | | | | | | | |
| | Bacteria | Bacteroidetes | Bacteroidia | | | | | postHDP2 | 4.6 | | | | | | | | | | |

Supplemental Table S5. continued

| Group 1 vs Group 2 | Domain | Phylum | Class | Order | Family | Genus | Level | Higher | LDA | Phylotype-OTU | Mean 1 | Var 1 | SD 1 | Mean 2 | Var 2 | SD 2 | Relative abundance (RA, %) | >=0.1% RA |
|---|---|---|---|---|---|---|---|---|---|---|---|---|---|---|---|---|---|---|
| | Bacteria | Firmicutes | Bacilli | Bacillales | Bacillales_Incertae_Sedis_XI | | | pre | 3.9 | | | | | | | | | |
| | Bacteria | Firmicutes | Clostridia | | | | | pre | 4.7 | | | | | | | | | |
| | Bacteria | Firmicutes | Clostridia | Clostridiales | | | | pre | 4.7 | | | | | | | | | |
| | Bacteria | Firmicutes | Clostridia | Clostridiales | Peptostreptococcaceae | | | pre | 3.4 | | | | | | | | | |
| | Bacteria | Bacteroidetes | | | | | | post | 4.7 | | | | | | | | | |
| | Bacteria | Firmicutes | | | | | | pre | 4.7 | | | | | | | | | |
| | Bacteria | Bacteroidetes | Bacteroidia | Bacteroidales | | | | post | 4.7 | | | | | | | | | |
| | Bacteria | Bacteroidetes | Bacteroidia | | | | | post | 4.7 | | | | | | | | | |
| | Bacteria | Actinobacteria | Coriobacteriia | Coriobacteriales | Coriobacteriaceae | | | pre | 3.4 | | | | | | | | | |
| | Bacteria | Actinobacteria | Coriobacteriia | Coriobacteriales | | | | pre | 3.4 | | | | | | | | | |
| | Bacteria | Firmicutes | Clostridia | Clostridiales | Lachnospiraceae | | | pre | 4.7 | | | | | | | | | |
| | Bacteria | Bacteroidetes | Bacteroidia | Bacteroidales | Porphyromonadaceae | | | post | 3.4 | | | | | | | | | |
| | Bacteria | Actinobacteria | Coriobacteriia | | | | | pre | 3.5 | | | | | | | | | |
| | Bacteria | Firmicutes | Bacilli | Bacillales | | | | pre | 3.2 | | | | | | | | | |
| | Bacteria | Lentisphaerae | Lentisphaeria | Victivallales | | | | post | 3.5 | | | | | | | | | |
| | Bacteria | Lentisphaerae | Lentisphaeria | | | | | post | 3.5 | | | | | | | | | |
| | Bacteria | Lentisphaerae | Lentisphaeria | Victivallales | Victivallaceae | | | post | 3.4 | | | | | | | | | |
| | Bacteria | Lentisphaerae | | | | | | post | 3.5 | | | | | | | | | |
| Washout, pre-HDPs 2&3 vs post-HDPs 1&2 | Bacteria | Verrucomicrobia | Verrucomicrobiae | Verrucomicrobiales | | | | post | 3.6 | | | | | | | | | |
| | Bacteria | Verrucomicrobia | Verrucomicrobiae | Verrucomicrobiales | Akkermansiaceae | | | post | 3.6 | | | | | | | | | |
| | Bacteria | Verrucomicrobia | Verrucomicrobiae | | | | | post | 3.6 | | | | | | | | | |
| | Bacteria | Verrucomicrobia | | | | | | post | 3.7 | | | | | | | | | |

Notes: HDP, healthy dietary pattern.

Supplemental Table S6. OTUs that significantly differed between groups based on LEfSe analyses.

| Group 1 vs Group 2 | OTU | Domain | Phylum | Class | Order | Family | Genus | Abundant in | LDA | Mean 1 | Var 1 | SD 1 | Mean 2 | Var 2 | SD 2 | RA, % | >=0.1 % RA |
|---|---|---|---|---|---|---|---|---|---|---|---|---|---|---|---|---|---|
| All_pre vs post | OTU0006 | Bacteria | Firmicutes | Clostridia | Clostridiales | Lachnospiraceae | Blautia | pre | 3.67363 | 0.039833 | 0.001071 | 0.003509 | 0.030773 | 0.000722 | 0.002864 | 2.13473132 | Yes |
| All_pre vs post | OTU0017 | Bacteria | Firmicutes | Clostridia | Clostridiales | Lachnospiraceae | Dorea | pre | 3.85471 | 0.024086 | 0.000272 | 0.00177 | 0.014685 | 0.00024 | 0.001652 | 1.171481 | Yes |
| All_pre vs post | OTU0025 | Bacteria | Actinobacteria | Coriobacteriia | Coriobacteriales | Coriobacteriaceae | Collinsella | pre | 3.45094 | 0.013265 | 0.000302 | 0.001862 | 0.008018 | 0.000129 | 0.001211 | 0.64304063 | Yes |
| All_pre vs post | OTU0026 | Bacteria | Firmicutes | Clostridia | Clostridiales | Lachnospiraceae | Mediterraneibacter | pre | 3.61994 | 0.013798 | 0.000247 | 0.001685 | 0.005585 | 0.000044 | 0.000709 | 0.58505898 | Yes |
| All_pre vs post | OTU0032 | Bacteria | Firmicutes | Clostridia | Clostridiales | Lachnospiraceae | Mediterraneibacter | pre | 3.37702 | 0.007796 | 0.000093 | 0.001036 | 0.005237 | 0.000061 | 0.000836 | 0.39391874 | Yes |
| All_pre vs post | OTU0034 | Bacteria | Firmicutes | Clostridia | Clostridiales | Lachnospiraceae | Lachnospiraceae_unclassified | post | 3.24333 | 0.005057 | 0.000058 | 0.000818 | 0.008583 | 0.00014 | 0.001263 | 0.41331586 | Yes |
| All_pre vs post | OTU0035 | Bacteria | Firmicutes | Clostridia | Clostridiales | Peptostreptococcaceae | Romboutsia | pre | 3.56989 | 0.010118 | 0.000249 | 0.001693 | 0.004421 | 0.000041 | 0.000686 | 0.43889908 | Yes |
| All_pre vs post | OTU0037 | Bacteria | Firmicutes | Clostridia | Clostridiales | Lachnospiraceae | Lachnospiraceae_unclassified | pre | 3.41508 | 0.008117 | 0.000108 | 0.001115 | 0.004896 | 0.000034 | 0.000619 | 0.3931848 | Yes |
| All_pre vs post | OTU0043 | Bacteria | Firmicutes | Clostridia | Clostridiales | Ruminococcaceae | Ruminococcaceae_unclassified | post | 2.69433 | 0.003597 | 0.000047 | 0.000736 | 0.006505 | 0.000091 | 0.001019 | 0.3061599 | Yes |
| All_pre vs post | OTU0058 | Bacteria | Bacteroidetes | Bacteroidia | Bacteroidales | Porphyromonadaceae | Parabacteroides | post | 2.90775 | 0.00184 | 0.000024 | 0.000522 | 0.003611 | 0.000089 | 0.001005 | 0.16524246 | Yes |
| All_pre vs post | OTU0061 | Bacteria | Firmicutes | Clostridia | Clostridiales | Lachnospiraceae | Lachnospiraceae_unclassified | post | 2.64727 | 0.002691 | 0.000016 | 0.000426 | 0.003111 | 0.000016 | 0.000423 | 0.17562254 | Yes |
| All_pre vs post | OTU0066 | Bacteria | Firmicutes | Clostridia | Clostridiales | Lachnospiraceae | Roseburia | post | 2.71227 | 0.001363 | 0.000006 | 0.000252 | 0.003291 | 0.000035 | 0.000631 | 0.14112713 | Yes |
| All_pre vs post | OTU0076 | Bacteria | Firmicutes | Clostridia | Clostridiales | Lachnospiraceae | Lachnospira | post | 2.77281 | 0.00192 | 0.000033 | 0.000616 | 0.002126 | 0.000013 | 0.000386 | 0.12246396 | Yes |
| All_pre vs post | OTU0088 | Bacteria | Firmicutes | Clostridia | Clostridiales | Peptostreptococcaceae | Peptostreptococcaceae_unclassified | pre | 2.27594 | 0.002217 | 0.000016 | 0.000431 | 0.001595 | 0.000013 | 0.000381 | 0.11522936 | Yes |
| post13, HDP 3 vs HDP 1 | OTU0050 | Bacteria | Firmicutes | Clostridia | Clostridiales | Lachnospiraceae | Blautia | Post HDP3 | 3.21715 | 0.007132 | 0.00027 | 0.003426 | 0.00264 | 0.000051 | 0.001229 | 0.19334207 | Yes |
| post13, HDP 3 vs HDP 1 | OTU0057 | Bacteria | Firmicutes | Clostridia | Clostridiales | Lachnospiraceae | Dorea | Post HDP3 | 3.32568 | 0.004377 | 0.000013 | 0.000739 | 0.002176 | 0.000003 | 0.000284 | 0.20529489 | Yes |
| post13, HDP 3 vs HDP 1 | OTU0063 | Bacteria | Bacteroidetes | Bacteroidia | Bacteroidales | Porphyromonadaceae | Parabacteroides | Post HDP1 | 2.98586 | 0.001477 | 0.000015 | 0.000797 | 0.003451 | 0.000015 | 0.000672 | 0.18516383 | Yes |
| post23, HDP 3 vs 2 | OTU0017 | Bacteria | Firmicutes | Clostridia | Clostridiales | Lachnospiraceae | Dorea | Post HDP3 | 3.75487 | 0.022609 | 0.000613 | 0.005163 | 0.010925 | 0.000077 | 0.001579 | 1.171481 | Yes |
| post23, HDP 3 vs 2 | OTU0034 | Bacteria | Firmicutes | Clostridia | Clostridiales | Lachnospiraceae | Lachnospiraceae_unclassified | Post HDP2 | 3.68898 | 0.004298 | 0.000023 | 0.000997 | 0.012011 | 0.000266 | 0.00293 | 0.41331586 | Yes |
| post23, HDP 3 vs 2 | OTU0057 | Bacteria | Firmicutes | Clostridia | Clostridiales | Lachnospiraceae | Dorea | Post HDP3 | 3.18505 | 0.004377 | 0.000013 | 0.000739 | 0.002778 | 0.000012 | 0.00063 | 0.20529489 | Yes |
| post23, HDP 3 vs 2 | OTU0063 | Bacteria | Bacteroidetes | Bacteroidia | Bacteroidales | Porphyromonadaceae | Parabacteroides | Post HDP2 | 3.18813 | 0.001477 | 0.000015 | 0.000797 | 0.002983 | 0.000013 | 0.000642 | 0.18516383 | Yes |

Supplemental Table S6. continued

| Group 1 vs Group 2 | OTU | Domain | Phylum | Class | Order | Family | Genus | Abundant in | LDA | Mean 1 | Var 1 | SD 1 | Mean 2 | Var 2 | SD 2 | RA, % | >=0.1% RA |
|---|---|---|---|---|---|---|---|---|---|---|---|---|---|---|---|---|---|
| pre13, HDP 3 vs 1 | OTU0023 | Bacteria | Firmicutes | Clostridia | Clostridiales | Lachnospiraceae | Blautia | preHDP3 | 2.0276 | 0.011178 | 0.000056 | 0.0016 | 0.009978 | 0.000185 | 0.00237 | 0.62899083 | Yes |
| pre13, HDP 3 vs 1 | OTU0050 | Bacteria | Firmicutes | Clostridia | Clostridiales | Lachnospiraceae | Blautia | preHDP3 | 3.2392 | 0.003707 | 0.000043 | 0.001391 | 0.001571 | 0.000023 | 0.000841 | 0.19334207 | Yes |
| pre13, HDP 3 vs 1 | OTU0073 | Bacteria | Firmicutes | Clostridia | Clostridiales | Lachnospiraceae | Lachnospiraceae _unclassified | preHDP3 | 2.86676 | 0.002784 | 0.000009 | 0.00065 | 0.001534 | 0.000006 | 0.000417 | 0.11984273 | Yes |
| pre23, HDP 3 vs 2 | OTU0012 | Bacteria | Bacteroidetes | Bacteroidia | Bacteroidales | Bacteroidaceae | Bacteroides | preHDP2 | 3.60879 | 0.009937 | 0.000151 | 0.002616 | 0.016506 | 0.000207 | 0.002542 | 1.32969856 | Yes |
| pre23, HDP 3 vs 2 | OTU0078 | Bacteria | Firmicutes | Clostridia | Clostridiales | Ruminococcaceae | Faecalibacterium | preHDP2 | 3.18894 | 0.001034 | 0.000009 | 0.000642 | 0.001601 | 0.00001 | 0.000552 | 0.13242464 | Yes |
| HDP1, pre vs post | OTU0017 | Bacteria | Firmicutes | Clostridia | Clostridiales | Lachnospiraceae | Dorea | preHDP1 | 3.96612 | 0.023596 | 0.000265 | 0.002832 | 0.012753 | 0.000093 | 0.001658 | 1.171481 | Yes |
| HDP1, pre vs post | OTU0025 | Bacteria | Actinobacteria | Coriobacteriia | Coriobacteriales | Coriobacteriaceae | Collinsella | preHDP1 | 3.44922 | 0.0163 | 0.000477 | 0.003801 | 0.008972 | 0.000253 | 0.002729 | 0.64304063 | Yes |
| HDP1, pre vs post | OTU0026 | Bacteria | Firmicutes | Clostridia | Clostridiales | Lachnospiraceae | Mediterraneibacter | preHDP1 | 3.8019 | 0.012753 | 0.000131 | 0.001991 | 0.004789 | 0.00003 | 0.000947 | 0.58505898 | Yes |
| HDP1, pre vs post | OTU0035 | Bacteria | Firmicutes | Clostridia | Clostridiales | Peptostreptococcaceae | Romboutsia | preHDP1 | 3.33086 | 0.012064 | 0.000515 | 0.00395 | 0.003326 | 0.00002 | 0.000765 | 0.43889908 | Yes |
| HDP1, pre vs post | OTU0057 | Bacteria | Firmicutes | Clostridia | Clostridiales | Lachnospiraceae | Dorea | preHDP1 | 3.1361 | 0.003905 | 0.000008 | 0.000493 | 0.002176 | 0.000003 | 0.000284 | 0.20529489 | Yes |
| HDP1, pre vs post | OTU0066 | Bacteria | Firmicutes | Clostridia | Clostridiales | Lachnospiraceae | Roseburia | postHDP1 | 3.20611 | 0.00124 | 0.000005 | 0.00037 | 0.004673 | 0.000064 | 0.001374 | 0.14112713 | Yes |
| HDP1, pre vs post | OTU0071 | Bacteria | Firmicutes | Clostridia | Clostridiales | Ruminococcaceae | Ruminococcaceae _unclassified | postHDP1 | 2.67687 | 0.001498 | 0.000003 | 0.000316 | 0.002854 | 0.000014 | 0.000634 | 0.13567497 | Yes |
| HDP1, pre vs post | OTU0076 | Bacteria | Firmicutes | Clostridia | Clostridiales | Lachnospiraceae | Lachnospira | preHDP1 | 3.14531 | 0.002609 | 0.000073 | 0.001491 | 0.00198 | 0.000007 | 0.000468 | 0.12246396 | Yes |
| HDP2, pre vs post | OTU0017 | Bacteria | Firmicutes | Clostridia | Clostridiales | Lachnospiraceae | Dorea | preHDP2 | 3.8101 | 0.022485 | 0.000201 | 0.002503 | 0.010925 | 0.000077 | 0.001579 | 1.171481 | Yes |
| HDP2, pre vs post | OTU0026 | Bacteria | Firmicutes | Clostridia | Clostridiales | Lachnospiraceae | Mediterraneibacter | preHDP2 | 3.63697 | 0.012583 | 0.000161 | 0.002245 | 0.004519 | 0.000021 | 0.000818 | 0.58505898 | Yes |
| HDP2, pre vs post | OTU0032 | Bacteria | Firmicutes | Clostridia | Clostridiales | Lachnospiraceae | Mediterraneibacter | preHDP2 | 3.09912 | 0.00669 | 0.000025 | 0.000886 | 0.00403 | 0.000025 | 0.000901 | 0.39391874 | Yes |
| HDP2, pre vs post | OTU0035 | Bacteria | Firmicutes | Clostridia | Clostridiales | Peptostreptococcaceae | Romboutsia | preHDP2 | 2.99785 | 0.008319 | 0.000077 | 0.001549 | 0.005047 | 0.000073 | 0.001533 | 0.43889908 | Yes |
| HDP3, pre vs post | OTU0026 | Bacteria | Firmicutes | Clostridia | Clostridiales | Lachnospiraceae | Mediterraneibacter | preHDP3 | 4.21471 | 0.017132 | 0.000558 | 0.005038 | 0.0082 | 0.000091 | 0.00199 | 0.58505898 | Yes |
| W, pre vs post | OTU0006 | Bacteria | Firmicutes | Clostridia | Clostridiales | Lachnospiraceae | Blautia | pre | 3.60741 | 0.041338 | 0.001173 | 0.00466 | 0.027271 | 0.000589 | 0.00301 | 2.13473132 | Yes |
| W, pre vs post | OTU0008 | Bacteria | Firmicutes | Clostridia | Clostridiales | Lachnospiraceae | Fusicatenibacter | pre | 3.59161 | 0.034381 | 0.000737 | 0.003693 | 0.023875 | 0.000292 | 0.00212 | 1.80068152 | Yes |
| W, pre vs post | OTU0017 | Bacteria | Firmicutes | Clostridia | Clostridiales | Lachnospiraceae | Dorea | pre | 3.55308 | 0.024386 | 0.000282 | 0.002285 | 0.011881 | 0.000085 | 0.001145 | 1.171481 | Yes |
| W, pre vs post | OTU0026 | Bacteria | Firmicutes | Clostridia | Clostridiales | Lachnospiraceae | Mediterraneibacter | pre | 3.67415 | 0.014436 | 0.000321 | 0.002437 | 0.00466 | 0.000025 | 0.000626 | 0.58505898 | Yes |
| W, pre vs post | OTU0032 | Bacteria | Firmicutes | Clostridia | Clostridiales | Lachnospiraceae | Mediterraneibacter | pre | 2.76317 | 0.008147 | 0.000112 | 0.001439 | 0.00417 | 0.000022 | 0.000576 | 0.39391874 | Yes |

Supplemental Table S6. continued

| Group 1 vs Group 2 | OTU | Domain | Phylum | Class | Order | Family | Genus | Abundant in | LDA | Mean 1 | Var 1 | SD 1 | Mean 2 | Var 2 | SD 2 | RA, % | >=0.1% RA |
|---|---|---|---|---|---|---|---|---|---|---|---|---|---|---|---|---|---|
| W, pre vs post | OTU0034 | Bacteria | Firmicutes | Clostridia | Clostridiales | Lachnospiraceae | Lachnospiraceae _unclassified | post | 2.71581 | 0.005857 | 0.000083 | 0.001241 | 0.010099 | 0.000174 | 0.001636 | 0.41331586 | Yes |
| W, pre vs post | OTU0035 | Bacteria | Firmicutes | Clostridia | Clostridiales | Peptostreptococcaceae | Romboutsia | pre | 3.44685 | 0.008928 | 0.00009 | 0.001291 | 0.004147 | 0.000045 | 0.000833 | 0.43889908 | Yes |
| W, pre vs post | OTU0039 | Bacteria | Bacteroidetes | Bacteroidia | Bacteroidales | Rikenellaceae | Alistipes | post | 3.24651 | 0.00356 | 0.00003 | 0.000741 | 0.00682 | 0.000093 | 0.001194 | 0.33709043 | Yes |
| W, pre vs post | OTU0043 | Bacteria | Firmicutes | Clostridia | Clostridiales | Ruminococcaceae | Ruminococcaceae _unclassified | post | 2.95713 | 0.00356 | 0.000057 | 0.001029 | 0.006293 | 0.00008 | 0.001109 | 0.3061599 | Yes |
| W, pre vs post | OTU0056 | Bacteria | Firmicutes | Clostridia | Clostridiales | Lachnospiraceae | Roseburia | pre | 3.23707 | 0.004486 | 0.000053 | 0.000994 | 0.001997 | 0.000007 | 0.000334 | 0.19501966 | Yes |
| W, pre vs post | OTU0057 | Bacteria | Firmicutes | Clostridia | Clostridiales | Lachnospiraceae | Dorea | pre | 2.46311 | 0.003779 | 0.000011 | 0.000443 | 0.002463 | 0.000007 | 0.000335 | 0.20529489 | Yes |
| W, pre vs post | OTU0058 | Bacteria | Bacteroidetes | Bacteroidia | Bacteroidales | Porphyromonadaceae | Parabacteroides | post | 3.01754 | 0.001325 | 0.000003 | 0.000239 | 0.004016 | 0.000113 | 0.001319 | 0.16524246 | Yes |
| W, pre vs post | OTU0066 | Bacteria | Firmicutes | Clostridia | Clostridiales | Lachnospiraceae | Roseburia | post | 3.27179 | 0.001437 | 0.000006 | 0.000339 | 0.003508 | 0.000041 | 0.000798 | 0.14112713 | Yes |
| W, pre vs post | OTU0076 | Bacteria | Firmicutes | Clostridia | Clostridiales | Lachnospiraceae | Lachnospira | post | 2.84951 | 0.001499 | 0.000009 | 0.000402 | 0.002426 | 0.000016 | 0.000502 | 0.12246396 | Yes |
| W, pre vs post | OTU0110 | Bacteria | Firmicutes | Erysipelotrichia | Erysipelotrichales | Erysipelotrichaceae | Turicibacter | pre | 2.77961 | 0.001668 | 0.000006 | 0.000319 | 0.00125 | 0.000015 | 0.000475 | 0.10076016 | Yes |
| W1, pre vs post | OTU0017 | Bacteria | Firmicutes | Clostridia | Clostridiales | Lachnospiraceae | Dorea | preHDP2 | 3.65353 | 0.022485 | 0.000201 | 0.002503 | 0.012753 | 0.000093 | 0.001658 | 1.171481 | Yes |
| W1, pre vs post | OTU0026 | Bacteria | Firmicutes | Clostridia | Clostridiales | Lachnospiraceae | Mediterraneibacter | preHDP2 | 3.62676 | 0.012583 | 0.000161 | 0.002245 | 0.004789 | 0.00003 | 0.000947 | 0.58505898 | Yes |
| W1, pre vs post | OTU0032 | Bacteria | Firmicutes | Clostridia | Clostridiales | Lachnospiraceae | Mediterraneibacter | preHDP2 | 3.44031 | 0.00669 | 0.000025 | 0.000886 | 0.004299 | 0.000019 | 0.000747 | 0.39391874 | Yes |
| W1, pre vs post | OTU0035 | Bacteria | Firmicutes | Clostridia | Clostridiales | Peptostreptococcaceae | Romboutsia | preHDP2 | 3.52397 | 0.008319 | 0.000077 | 0.001549 | 0.003326 | 0.00002 | 0.000765 | 0.43889908 | Yes |
| W1, pre vs post | OTU0056 | Bacteria | Firmicutes | Clostridia | Clostridiales | Lachnospiraceae | Roseburia | preHDP2 | 3.4756 | 0.004122 | 0.000037 | 0.001082 | 0.001908 | 0.000008 | 0.00049 | 0.19501966 | Yes |
| W1, pre vs post | OTU0057 | Bacteria | Firmicutes | Clostridia | Clostridiales | Lachnospiraceae | Dorea | preHDP2 | 3.19561 | 0.003648 | 0.00001 | 0.000561 | 0.002176 | 0.000003 | 0.000284 | 0.20529489 | Yes |
| W2, pre vs post | OTU0006 | Bacteria | Firmicutes | Clostridia | Clostridiales | Lachnospiraceae | Blautia | preHDP3 | 4.09052 | 0.043787 | 0.000962 | 0.006613 | 0.028561 | 0.000847 | 0.005226 | 2.13473132 | Yes |
| W2, pre vs post | OTU0017 | Bacteria | Firmicutes | Clostridia | Clostridiales | Lachnospiraceae | Dorea | preHDP3 | 3.74755 | 0.027151 | 0.000402 | 0.004276 | 0.010925 | 0.000077 | 0.001579 | 1.171481 | Yes |
| W2, pre vs post | OTU0026 | Bacteria | Firmicutes | Clostridia | Clostridiales | Lachnospiraceae | Mediterraneibacter | preHDP3 | 3.60534 | 0.017132 | 0.000558 | 0.005038 | 0.004519 | 0.000021 | 0.000818 | 0.58505898 | Yes |
| W2, pre vs post | OTU0034 | Bacteria | Firmicutes | Clostridia | Clostridiales | Lachnospiraceae | Lachnospiraceae _unclassified | postHDP2 | 3.80422 | 0.002743 | 0.000014 | 0.000797 | 0.012011 | 0.000266 | 0.00293 | 0.41331586 | Yes |
| W2, pre vs post | OTU0035 | Bacteria | Firmicutes | Clostridia | Clostridiales | Peptostreptococcaceae | Romboutsia | preHDP3 | 2.58201 | 0.009813 | 0.000112 | 0.00226 | 0.005047 | 0.000073 | 0.001533 | 0.43889908 | Yes |
| W2, pre vs post | OTU0038 | Bacteria | Firmicutes | Erysipelotrichia | Erysipelotrichales | Erysipelotrichaceae | Faecalibacillus | preHDP3 | 3.42793 | 0.012583 | 0.00075 | 0.00584 | 0.003218 | 0.000024 | 0.000882 | 0.39454784 | Yes |
| W2, pre vs post | OTU0044 | Bacteria | Firmicutes | Clostridia | Clostridiales | Ruminococcaceae | Ruminococcaceae _unclassified | postHDP2 | 3.54104 | 0.002853 | 0.000024 | 0.001038 | 0.008206 | 0.000137 | 0.002104 | 0.36498034 | Yes |

Supplemental Table S6. continued

| **Group 1 vs Group 2** | **OTU** | **Domain** | **Phylum** | **Class** | **Order** | **Family** | **Genus** | **Abundant in** | **LDA** | **Mean 1** | **Var 1** | **SD 1** | **Mean 2** | **Var 2** | **SD 2** | **RA, %** | **>=0.1 % RA** |
|---|---|---|---|---|---|---|---|---|---|---|---|---|---|---|---|---|---|
| W2, pre vs post | OTU0058 | Bacteria | Bacteroidetes | Bacteroidia | Bacteroidales | Porphyromonadaceae | Parabacteroides | postHDP2 | 3.12151 | 0.001089 | 0.000003 | 0.000366 | 0.005556 | 0.000225 | 0.002694 | 0.16524246 | Yes |
| W2, pre vs post | OTU0076 | Bacteria | Firmicutes | Clostridia | Clostridiales | Lachnospiraceae | Lachnospira | postHDP2 | 2.99944 | 0.001006 | 0.000004 | 0.000444 | 0.002915 | 0.000026 | 0.000921 | 0.12246396 | Yes |
| W2, pre vs post | OTU0110 | Bacteria | Firmicutes | Erysipelotrichia | Erysipelotrichales | Erysipelotrichaceae | Turicibacter | preHDP3 | 3.05119 | 0.002122 | 0.000007 | 0.000552 | 0.000548 | 0.000001 | 0.0002 | 0.10076016 | Yes |
| All_pre vs post | OTU0086 | Bacteria | Firmicutes | Clostridia | Clostridiales | Lachnospiraceae | Lachnospiraceae _unclassified | post | 2.69485 | 0.000739 | 0.000004 | 0.000223 | 0.00193 | 0.000013 | 0.000385 | 0.08094364 | No |
| All_pre vs post | OTU0125 | Bacteria | Firmicutes | Clostridia | Clostridiales | Lachnospiraceae | Ruminococcus2 | pre | 2.74647 | 0.001011 | 0.000002 | 0.000153 | 0.000737 | 0.000003 | 0.000175 | 0.05284404 | No |
| All_pre vs post | OTU0138 | Bacteria | Actinobacteria | Coriobacteriia | Eggerthellales | Eggerthellaceae | Adlercreutzia | pre | 2.55024 | 0.000809 | 0.000002 | 0.000134 | 0.000448 | 0.000001 | 0.000084 | 0.03795544 | No |
| All_pre vs post | OTU0180 | Bacteria | Firmicutes | Clostridia | Clostridiales | Ruminococcaceae | Ruminococcaceae _unclassified | post | 2.62114 | 0.000289 | 0 | 0.000059 | 0.000479 | 0 | 0.000069 | 0.02327654 | No |
| post13, HDP 3 vs HDP 1 | OTU0145 | Bacteria | Firmicutes | Clostridia | Clostridiales | Lachnospiraceae | Lachnospiraceae _unclassified | postHDP1 | 3.03372 | 0.000224 | 0 | 0.000086 | 0.001106 | 0.000005 | 0.000383 | 0.03470511 | No |
| pre12, HDP 2 vs 1 | OTU0115 | Bacteria | Firmicutes | Clostridia | Clostridiales | Lachnospiraceae | Lachnospiraceae _unclassified | preHDP2 | 2.43174 | 0.001232 | 0.000002 | 0.000272 | 0.001057 | 0.000007 | 0.000444 | 0.06783748 | No |
| pre12, HDP 2 vs 1 | OTU0189 | Bacteria | Firmicutes | Clostridia | Clostridiales | Ruminococcaceae | Ruminococcaceae _unclassified | preHDP1 | 2.92913 | 0.000332 | 0 | 0.000115 | 0.000919 | 0.000007 | 0.000462 | 0.02495413 | No |
| pre123 | OTU0167 | Bacteria | Firmicutes | Bacilli | Lactobacillales | Streptococcaceae | Streptococcus | preHDP3 | 3.25409 | NA | NA | NA | NA | NA | NA | 0.0295675 | No |
| pre13, HDP 3 vs 1 | OTU0152 | Bacteria | Bacteroidetes | Bacteroidia | Bacteroidales | Odoribacteraceae | Butyricimonas | preHDP1 | 2.58272 | 0.000289 | 0 | 0.00012 | 0.001002 | 0.000002 | 0.000259 | 0.04214941 | No |
| pre13, HDP 3 vs 1 | OTU0167 | Bacteria | Firmicutes | Bacilli | Lactobacillales | Streptococcaceae | Streptococcus | preHDP3 | 2.65083 | 0.001475 | 0.000012 | 0.000736 | 0.000248 | 0 | 0.000072 | 0.0295675 | No |
| pre13, HDP 3 vs 1 | OTU0189 | Bacteria | Firmicutes | Clostridia | Clostridiales | Ruminococcaceae | Ruminococcaceae _unclassified | preHDP1 | 2.42685 | 0.000221 | 0 | 0.00006 | 0.000919 | 0.000007 | 0.000462 | 0.02495413 | No |
| pre23, HDP 3 vs 2 | OTU0099 | Bacteria | Firmicutes | Clostridia | Clostridiales | Lachnospiraceae | Lachnospiraceae _unclassified | preHDP3 | 2.91277 | 0.002012 | 0.000003 | 0.000363 | 0.001251 | 0.000002 | 0.000263 | 0.0797903 | No |
| pre23, HDP 3 vs 2 | OTU0152 | Bacteria | Bacteroidetes | Bacteroidia | Bacteroidales | Odoribacteraceae | Butyricimonas | preHDP2 | 3.08649 | 0.000289 | 0 | 0.00012 | 0.000749 | 0.000001 | 0.000162 | 0.04214941 | No |

Supplemental Table S6. continued

| Group 1 vs Group 2 | OTU | Domain | Phylum | Class | Order | Family | Genus | Abundant in | LDA | Mean 1 | Var 1 | SD 1 | Mean 2 | Var 2 | SD 2 | RA, % | >=0.1 % RA |
|---|---|---|---|---|---|---|---|---|---|---|---|---|---|---|---|---|---|
| HDP1, pre vs post | OTU0145 | Bacteria | Firmicutes | Clostridia | Clostridiales | Lachnospiraceae | Lachnospiraceae_unclassified | postHDP1 | 2.79261 | 0.000211 | 0 | 0.000081 | 0.001106 | 0.000005 | 0.000383 | 0.03470511 | No |
| HDP1, pre vs post | OTU0233 | Bacteria | Firmicutes | Clostridia | Clostridiales | Ruminococcaceae | Lawsonibacter | postHDP1 | 2.55977 | 0.000147 | 0 | 0.000048 | 0.00041 | 0 | 0.000091 | 0.01614679 | No |
| HDP2, pre vs post | OTU0125 | Bacteria | Firmicutes | Clostridia | Clostridiales | Lachnospiraceae | Ruminococcus2 | preHDP2 | 2.48805 | 0.001042 | 0.000002 | 0.000258 | 0.000606 | 0.000004 | 0.000363 | 0.05284404 | No |
| W, pre vs post | OTU0086 | Bacteria | Firmicutes | Clostridia | Clostridiales | Lachnospiraceae | Lachnospiraceae_unclassified | post | 2.39577 | 0.000584 | 0.000002 | 0.000188 | 0.002164 | 0.000016 | 0.000501 | 0.08094364 | No |
| W, pre vs post | OTU0099 | Bacteria | Firmicutes | Clostridia | Clostridiales | Lachnospiraceae | Lachnospiraceae_unclassified | pre | 2.77788 | 0.001561 | 0.000003 | 0.000219 | 0.000933 | 0.000001 | 0.000113 | 0.0797903 | No |
| W, pre vs post | OTU0125 | Bacteria | Firmicutes | Clostridia | Clostridiales | Lachnospiraceae | Ruminococcus2 | pre | 2.45866 | 0.001134 | 0.000003 | 0.000225 | 0.000695 | 0.000003 | 0.000218 | 0.05284404 | No |
| W, pre vs post | OTU0138 | Bacteria | Actinobacteria | Coriobacteriia | Eggerthellales | Eggerthellaceae | Adlercreutzia | pre | 2.63994 | 0.000887 | 0.000002 | 0.000181 | 0.000359 | 0 | 0.000085 | 0.03795544 | No |
| W, pre vs post | OTU0145 | Bacteria | Firmicutes | Clostridia | Clostridiales | Lachnospiraceae | Lachnospiraceae_unclassified | post | 2.57682 | 0.000522 | 0.000002 | 0.000172 | 0.000924 | 0.000004 | 0.00024 | 0.03470511 | No |
| W, pre vs post | OTU0180 | Bacteria | Firmicutes | Clostridia | Clostridiales | Ruminococcaceae | Ruminococcaceae_unclassified | post | 2.72793 | 0.000314 | 0 | 0.00008 | 0.000518 | 0 | 0.000083 | 0.02327654 | No |
| W, pre vs post | OTU0192 | Bacteria | Firmicutes | Clostridia | Clostridiales | Ruminococcaceae | Dysosmobacter | post | 2.66528 | 0.000258 | 0 | 0.000079 | 0.000411 | 0 | 0.00008 | 0.01950197 | No |
| W1, pre vs post | OTU0143 | Bacteria | Firmicutes | Clostridia | Clostridiales | Ruminococcaceae | Intestinimonas | preHDP2 | 3.07285 | 0.001118 | 0.000002 | 0.000235 | 0.000428 | 0 | 0.000117 | 0.04739187 | No |
| W2, pre vs post | OTU0086 | Bacteria | Firmicutes | Clostridia | Clostridiales | Lachnospiraceae | Lachnospiraceae_unclassified | postHDP2 | 3.00411 | 0.000455 | 0.000002 | 0.000282 | 0.002435 | 0.000021 | 0.000831 | 0.08094364 | No |
| W2, pre vs post | OTU0099 | Bacteria | Firmicutes | Clostridia | Clostridiales | Lachnospiraceae | Lachnospiraceae_unclassified | preHDP3 | 2.90189 | 0.002012 | 0.000003 | 0.000363 | 0.001017 | 0.000001 | 0.000176 | 0.0797903 | No |
| W2, pre vs post | OTU0125 | Bacteria | Firmicutes | Clostridia | Clostridiales | Lachnospiraceae | Ruminococcus2 | preHDP3 | 2.39972 | 0.001268 | 0.000004 | 0.00041 | 0.000606 | 0.000004 | 0.000363 | 0.05284404 | No |
| W2, pre vs post | OTU0138 | Bacteria | Actinobacteria | Coriobacteriia | Eggerthellales | Eggerthellaceae | Adlercreutzia | preHDP3 | 2.79171 | 0.001075 | 0.000002 | 0.0003 | 0.000313 | 0 | 0.000115 | 0.03795544 | No |
| W2, pre vs post | OTU0167 | Bacteria | Firmicutes | Bacilli | Lactobacillales | Streptococcaceae | Streptococcus | preHDP3 | 2.98937 | 0.001475 | 0.000012 | 0.000736 | 0.00044 | 0.000001 | 0.000151 | 0.0295675 | No |
| W2, pre vs post | OTU0180 | Bacteria | Firmicutes | Clostridia | Clostridiales | Ruminococcaceae | Ruminococcaceae_unclassified | postHDP2 | 2.39227 | 0.000248 | 0 | 0.000142 | 0.000469 | 0 | 0.000095 | 0.02327654 | No |
| W2, pre vs post | OTU0246 | Bacteria | Firmicutes | Clostridia | Clostridiales | Ruminococcaceae | Ruminococcaceae_unclassified | preHDP3 | 2.92727 | 0 | 0 | 0 | 0.000489 | 0.000003 | 0.000299 | 0.02222805 | No |
| post12 | None | #N/A | | | | | | NA | NA | NA | NA | NA | NA | NA | NA | NA | NA |

Notes: HDP, healthy dietary pattern.

Supplemental Table S7. Comparison of post/pre fold changes of selected taxa with at least 0.1% of relative abundance among HDPs 1-3 and between washouts 1-2.

| **Phylotype-OTU** | **Domain** | **Phylum** | **Class** | **Order** | **Family** | **Genus** | **Shapiro-Wilk test of post/pre p-value** | **p-value after log2 transformation** | **Levene's test for Homogeneity of Variance p-value** | **Anova p-value, HDP** | **Welch Two Sample t-test p-value, washout** |
|---|---|---|---|---|---|---|---|---|---|---|---|
| OTU003 | Bacteria | Firmicutes | Clostridia | Clostridiales | Lachnospiraceae | Blautia | <0.001 | 0.81 | NA | 0.84 | 0.84 |
| OTU009 | Bacteria | Firmicutes | Clostridia | Clostridiales | Clostridiales_unclassified | Clostridiales_unclassified | <0.001 | 0.33 | NA | 0.09 | 0.39 |
| OTU011 | Bacteria | Firmicutes | Clostridia | Clostridiales | Lachnospiraceae | Anaerostipes | <0.001 | 0.52 | NA | 0.58 | 0.82 |
| OTU013 | Bacteria | Firmicutes | Clostridia | Clostridiales | Lachnospiraceae | Dorea | <0.001 | 0.08 | NA | 0.99 | 0.84 |
| OTU014 | Bacteria | Firmicutes | Clostridia | Clostridiales | Lachnospiraceae | Roseburia | <0.001 | 0.52 | NA | 0.47 | 0.74 |
| OTU016 | Bacteria | Bacteroidetes | Bacteroidia | Bacteroidales | Porphyromonadaceae | Parabacteroides | <0.001 | 0.73 | NA | 0.58 | 0.69 |
| OTU018 | Bacteria | Firmicutes | Clostridia | Clostridiales | Lachnospiraceae | Mediterraneibacter | <0.001 | 0.19 | NA | 0.74 | 0.91 |
| OTU020 | Bacteria | Bacteria_unclassified | Bacteria_unclassified | Bacteria_unclassified | Bacteria_unclassified | Bacteria_unclassified | <0.001 | 0.77 | NA | 0.20 | 0.31 |
| OTU021 | Bacteria | Firmicutes | Clostridia | Clostridiales | Lachnospiraceae | Coprococcus | <0.001 | 0.29 | NA | 0.91 | 0.92 |
| OTU023 | Bacteria | Firmicutes | Bacilli | Lactobacillales | Streptococcaceae | Streptococcus | <0.001 | 0.02 | 0.89 | 0.11 | 0.13 |
| OTU024 | Bacteria | Actinobacteria | Coriobacteriia | Coriobacteriales | Coriobacteriaceae | Collinsella | <0.001 | 0.15 | NA | 0.81 | 0.74 |
| OTU026 | Bacteria | Firmicutes | Clostridia | Clostridiales | Ruminococcaceae | Ruminococcus | <0.001 | 0.15 | NA | 0.91 | 0.74 |
| OTU030 | Bacteria | Firmicutes | Clostridia | Clostridiales | Peptostreptococcaceae | Romboutsia | <0.001 | 0.62 | NA | 0.79 | 0.85 |
| OTU032 | Bacteria | Verrucomicrobia | Verrucomicrobiae | Verrucomicrobiales | Akkermansiaceae | Akkermansia | <0.001 | 0.01 | 0.72 | 0.32 | 0.98 |
| OTU035 | Bacteria | Firmicutes | Erysipelotrichia | Erysipelotrichales | Erysipelotrichaceae | Faecalibacillus | <0.001 | 0.21 | NA | 0.18 | 0.21 |
| OTU038 | Bacteria | Firmicutes | Clostridia | Clostridiales | Lachnospiraceae | Lachnospira | <0.001 | 0.86 | NA | 0.61 | 0.20 |
| OTU049 | Bacteria | Firmicutes | Clostridia | Clostridiales | Ruminococcaceae | Clostridium_IV | <0.001 | 0.21 | NA | 0.55 | 0.76 |
| OTU054 | Bacteria | Firmicutes | Erysipelotrichia | Erysipelotrichales | Erysipelotrichaceae | Turicibacter | <0.001 | <0.01 | 0.98 | 0.88 | 0.44 |
| OTU065 | Bacteria | Bacteroidetes | Bacteroidetes_unclassified | Bacteroidetes_unclassified | Bacteroidetes_unclassified | Bacteroidetes_unclassified | <0.001 | <0.01 | 0.81 | 0.28 | 0.65 |

Notes: HDP, healthy dietary pattern.

Supplemental Table S8. Comparison of post/pre fold changes of selected OTUs with at least 0.1% of relative abundance among HDPs 1-3 and between washouts 1-2.

| OTU | Domain | Phylum | Class | Order | Family | Genus | **Shapiro-Wilk test of post/pre p-value** | **p-value after log2 transformation** | **Levene's test for Homogeneity of Variance p-value** | **Anova p-value, HDP** | **Welch Two Sample t-test p-value, washout** |
|---|---|---|---|---|---|---|---|---|---|---|---|
| OTU0006 | Bacteria | Firmicutes | Clostridia | Clostridiales | Lachnospiraceae | Blautia | <0.001 | <0.01 | 0.66 | 0.24 | 0.51 |
| OTU0008 | Bacteria | Firmicutes | Clostridia | Clostridiales | Lachnospiraceae | Fusicatenibacter | <0.001 | 0.15 | NA | 0.56 | 0.64 |
| OTU0012 | Bacteria | Bacteroidetes | Bacteroidia | Bacteroidales | Bacteroidaceae | Bacteroides | <0.001 | 0.23 | NA | 0.64 | 0.46 |
| OTU0017 | Bacteria | Firmicutes | Clostridia | Clostridiales | Lachnospiraceae | Dorea | <0.001 | 0.34 | NA | 0.44 | 0.55 |
| OTU0023 | Bacteria | Firmicutes | Clostridia | Clostridiales | Lachnospiraceae | Blautia | <0.001 | 0.45 | NA | 0.54 | 0.98 |
| OTU0025 | Bacteria | Actinobacteria | Coriobacteriia | Coriobacteriales | Coriobacteriaceae | Collinsella | <0.001 | 0.36 | NA | 0.81 | 0.70 |
| OTU0026 | Bacteria | Firmicutes | Clostridia | Clostridiales | Lachnospiraceae | Mediterraneibacter | <0.001 | 0.65 | NA | 0.48 | 0.97 |
| OTU0032 | Bacteria | Firmicutes | Clostridia | Clostridiales | Lachnospiraceae | Mediterraneibacter | <0.001 | 0.53 | NA | 0.31 | 0.78 |
| OTU0034 | Bacteria | Firmicutes | Clostridia | Clostridiales | Lachnospiraceae | Lachnospiraceae_unclassified | <0.001 | 0.39 | NA | 0.84 | 0.06 |
| OTU0035 | Bacteria | Firmicutes | Clostridia | Clostridiales | Peptostreptococcaceae | Romboutsia | <0.001 | 0.32 | NA | 0.80 | 0.79 |
| OTU0037 | Bacteria | Firmicutes | Clostridia | Clostridiales | Lachnospiraceae | Lachnospiraceae_unclassified | <0.001 | <0.01 | 0.96 | 0.29 | 0.43 |
| OTU0038 | Bacteria | Firmicutes | Erysipelotrichia | Erysipelotrichales | Erysipelotrichaceae | Faecalibacillus | <0.001 | 0.07 | NA | 0.44 | 0.16 |
| OTU0039 | Bacteria | Bacteroidetes | Bacteroidia | Bacteroidales | Rikenellaceae | Alistipes | <0.001 | 0.61 | NA | 0.83 | 0.44 |
| OTU0043 | Bacteria | Firmicutes | Clostridia | Clostridiales | Ruminococcaceae | Ruminococcaceae_unclassified | <0.001 | <0.01 | 0.50 | 0.56 | 0.18 |
| OTU0044 | Bacteria | Firmicutes | Clostridia | Clostridiales | Ruminococcaceae | Ruminococcaceae_unclassified | <0.001 | 0.40 | NA | 0.57 | 0.60 |
| OTU0050 | Bacteria | Firmicutes | Clostridia | Clostridiales | Lachnospiraceae | Blautia | <0.001 | <0.01 | 0.39 | 0.42 | 0.83 |
| OTU0056 | Bacteria | Firmicutes | Clostridia | Clostridiales | Lachnospiraceae | Roseburia | <0.001 | 0.76 | NA | 0.61 | 0.50 |
| OTU0057 | Bacteria | Firmicutes | Clostridia | Clostridiales | Lachnospiraceae | Dorea | <0.001 | 0.03 | 0.98 | 0.08 | 0.92 |
| OTU0058 | Bacteria | Bacteroidetes | Bacteroidia | Bacteroidales | Porphyromonadaceae | Parabacteroides | <0.001 | 0.04 | 0.70 | 0.82 | 0.69 |
| OTU0061 | Bacteria | Firmicutes | Clostridia | Clostridiales | Lachnospiraceae | Lachnospiraceae_unclassified | <0.001 | 0.60 | NA | 0.70 | 0.16 |
| OTU0063 | Bacteria | Bacteroidetes | Bacteroidia | Bacteroidales | Porphyromonadaceae | Parabacteroides | <0.001 | 0.20 | NA | 0.37 | 0.53 |
| OTU0066 | Bacteria | Firmicutes | Clostridia | Clostridiales | Lachnospiraceae | Roseburia | <0.001 | 0.61 | NA | 0.53 | 0.95 |
| OTU0071 | Bacteria | Firmicutes | Clostridia | Clostridiales | Ruminococcaceae | Ruminococcaceae_unclassified | <0.001 | 0.03 | 0.76 | 0.11 | 0.41 |
| OTU0073 | Bacteria | Firmicutes | Clostridia | Clostridiales | Lachnospiraceae | Lachnospiraceae_unclassified | <0.001 | 0.02 | 0.27 | 0.23 | 0.72 |
| OTU0076 | Bacteria | Firmicutes | Clostridia | Clostridiales | Lachnospiraceae | Lachnospira | <0.001 | 0.66 | NA | 0.40 | 0.14 |
| OTU0078 | Bacteria | Firmicutes | Clostridia | Clostridiales | Ruminococcaceae | Faecalibacterium | <0.001 | <0.01 | 0.76 | 0.77 | 0.59 |

Notes: HDP, healthy dietary pattern.

Supplemental Table S9. Participant responses in cardiovascular risk factors from consuming study diets for 3 times (HDPs 1-3). Results are presented as LS means ± SEM.

| | HDP 1 | | | | | | | | | Washout 1 | | | HDP 2 | | | | | | | | | Washout 2 | | | HDP 3 | | | | | | | | | p-value | |
|---|---|---|---|---|---|---|---|---|---|---|---|---|---|---|---|---|---|---|---|---|---|---|---|---|---|---|---|---|---|---|---|---|---|---|---|
| Outcomes [1] | Pre | SE | N | Post | SE | N | Change | SE | N | Change | SE | N | Pre | SE | N | Post | SE | N | Change | SE | N | Change | SE | N | Pre | SE | N | Post | SE | N | Change | SE | N | time | time by HDP |
| WHC | 0.87 | 0.01 | 17 | 0.86 | 0.01 | 17 | -0.01 | 0.01 | 17 | 0.01 | 0.01 | 17 | 0.87 | 0.01 | 17 | 0.86 | 0.01 | 17 | -0.01 | 0.01 | 17 | 0.0005 | 0.01 | 12 | 0.86 | 0.01 | 12 | 0.86 | 0.01 | 12 | 0.001 | 0.01 | 12 | 0.45 | 0.35 |
| SAD [2] | 18.9 | 0.3 | 17 | 18.9 | 0.3 | 17 | 0.03 | 0.2 | 17 | 0.3 | 0.3 | 17 | 19.3 | 0.3 | 17 | 18.8 | 0.3 | 17 | -0.4 | 0.2 | 17 | 0.3 | 0.3 | 12 | 19.1 | 0.3 | 12 | 19.2 | 0.3 | 12 | 0.02 | 0.2 | 12 | 0.59 | 0.06 |
| SysBP | 108 a,b | 1.2 | 17 | 109 c,d | 1.2 | 17 | 0.9 | 1.4 | 17 | -1.8 | 1.5 | 17 | 107 a | 1.2 | 17 | 106 c | 1.2 | 17 | -1.0 | 1.4 | 17 | 4.2 | 1.6 | 12 | 110 b | 1.3 | 12 | 110 d | 1.4 | 12 | -0.3 | 1.6 | 12 | 0.93 | 0.41 |
| DiaBP | 68 a | 1.3 | 17 | 67 c | 1.3 | 17 | -0.8 | 1.4 | 17 | 0.5 | 1.5 | 17 | 68 a | 1.3 | 17 | 67 c | 1.3 | 17 | -0.8 | 1.4 | 17 | 5.3* | 1.6 | 12 | 72 b | 1.4 | 12 | 70 d | 1.4 | 12 | -2.2 | 1.5 | 12 | 0.33 | 0.45 |
| TC | 163 | 5.3 | 17 | 146 a | 5.1 | 17 | -17.4* | 5.0 | 17 | 19.8* | 5.4 | 17 | 165 | 5.3 | 17 | 150 a | 5.0 | 17 | -15.7* | 5.0 | 17 | 21.6* | 5.7 | 12 | 171 | 5.6 | 12 | 158 b | 5.2 | 12 | -13.3* | 5.2 | 12 | 0.01 | 0.49 |
| TAG [2] | 82.6 | 6.9 | 17 | 71.2 | 6.1 | 17 | -11.4 | 6.8 | 17 | 10.9 | 7.5 | 17 | 82.2 | 6.9 | 17 | 65.5 | 6.0 | 17 | -16.6* | 6.8 | 17 | 13.1 | 8.0 | 12 | 78.7 | 7.4 | 12 | 70.9 | 6.4 | 12 | -7.8 | 7.4 | 12 | 0.08 | 0.59 |
| HDLC | 53 | 1.9 | 17 | 50 | 2.0 | 17 | -3.5 | 1.9 | 17 | 3.6 | 2.1 | 17 | 53 | 1.9 | 17 | 52 | 2.0 | 17 | -1.3 | 1.9 | 17 | 2.1 | 2.3 | 12 | 54 | 2.1 | 12 | 52 | 2.1 | 12 | -2.2 | 2.0 | 12 | 0.20 | 0.29 |
| LDLC | 93 a | 4.6 | 17 | 82 c | 3.7 | 17 | -11.6* | 4.2 | 17 | 13.9* | 4.6 | 17 | 96 a,b | 4.6 | 17 | 85 c | 3.7 | 17 | -11.0* | 4.2 | 17 | 16.7* | 4.8 | 12 | 101 b | 4.8 | 12 | 91.7 d | 3.9 | 12 | -9.7* | 4.5 | 12 | 0.02 | 0.82 |
| CHOL/HDLC [2] | 3.1 | 0.2 | 17 | 3.0 | 0.1 | 17 | -0.1 | 0.1 | 17 | 0.2 | 0.2 | 17 | 3.2 | 0.2 | 17 | 3.0 | 0.1 | 17 | -0.3 | 0.1 | 17 | 0.3 | 0.2 | 12 | 3.3 | 0.2 | 12 | 3.1 | 0.1 | 12 | -0.2 | 0.2 | 12 | 0.18 | 0.51 |
| Glucose | 86.2 | 1.5 | 17 | 84.1 | 1.4 | 17 | -2.1 | 1.8 | 17 | 1.2 | 1.9 | 17 | 85.2 | 1.5 | 17 | 86.5 | 1.3 | 17 | 1.2 | 1.8 | 17 | 1.6 | 2.1 | 12 | 88.1 | 1.8 | 12 | 88.2 | 1.6 | 12 | 0.1 | 2.1 | 12 | 0.85 | 0.29 |
| BUN | 12.9 | 0.5 | 17 | 12.8 | 0.5 | 17 | -0.1 | 0.7 | 17 | 0.4 | 0.7 | 17 | 13.2 | 0.5 | 17 | 13.9 | 0.5 | 17 | 0.8 | 0.7 | 17 | -0.5 | 0.8 | 12 | 13.4 | 0.6 | 12 | 13.5 | 0.6 | 12 | 0.1 | 0.7 | 12 | 0.67 | 0.38 |
| Creatinine [2] | 0.9 | 0.02 | 17 | 0.9 | 0.02 | 17 | 0.01 | 0.02 | 17 | -0.04 | 0.02 | 17 | 0.8 | 0.02 | 17 | 0.9 | 0.02 | 17 | 0.01 | 0.02 | 17 | 0.01 | 0.02 | 12 | 0.9 | 0.02 | 12 | 0.9 | 0.02 | 12 | 0.01 | 0.02 | 12 | 0.54 | 0.84 |

[1] Data were analyzed using repeated-measures ANOVA adjusted for age, sex, and body mass index.
[2] Data were transformed before ANOVA analyses using Box Cox transformation with lambda of -1 for SAD, -0.5 for TAG and CHOL/HDLC ratio, and -1.25 for creatinine. Results are presented using untransformed LS means ± SEM.
Different superscripts denote significant difference among HDPs at pre (a and b) and at post (c and d); * significant change; Abbreviations: HDP, healthy dietary pattern; WHC, waist-to-hip circumference; SAD, sagittal abdominal diamter; SysBP, systolic blood pressure; DiaBP, diastolic blood pressure; TC, total cholesterol; TAG, triglycerides; HDLC, HDL-cholesterol; LDLC, LDL-cholesterol; BUN, blood urea nitrogen.

Supplemental Table S10. Raw (unadjusted) means and SD of participant responses in cardiovascular risk factors from consuming study diets for 3 times (HDPs 1-3).

| | HDP 1 | | | | | | | | | Washout 1 | | | HDP 2 | | | | | | | | | Washout 2 | | | HDP 3 | | | | | | | | |
|---|---|---|---|---|---|---|---|---|---|---|---|---|---|---|---|---|---|---|---|---|---|---|---|---|---|---|---|---|---|---|---|---|---|
| Outcomes | Pre | SD | N | Post | SD | N | Change | SD | N | Change | SD | N | Pre | SD | N | Post | SD | N | Change | SD | N | Change | SD | N | Pre | SD | N | Post | SD | N | Change | SD | N |
| WHC | 0.87 | 0.05 | 17 | 0.86 | 0.06 | 17 | -0.01 | 0.03 | 17 | 0.01 | 0.02 | 17 | 0.87 | 0.05 | 17 | 0.86 | 0.06 | 17 | -0.01 | 0.02 | 17 | -0.003 | 0.03 | 12 | 0.87 | 0.06 | 12 | 0.87 | 0.06 | 12 | -0.001 | 0.01 | 12 |
| SAD (cm) | 19.0 | 2.4 | 17 | 18.9 | 2.3 | 17 | -0.1 | 0.7 | 17 | 0.5 | 0.7 | 17 | 19.4 | 2.6 | 17 | 18,8 | 2.3 | 17 | -0.5 | 0.6 | 17 | 0.2 | 0.4 | 12 | 19.6 | 2.5 | 12 | 19.5 | 2.4 | 12 | -0.01 | 0.5 | 12 |
| SysBP (mm Hg) | 109 | 9.8 | 17 | 110 | 9.6 | 17 | 0.8 | 4.4 | 17 | -1.6 | 3.8 | 17 | 108 | 9.8 | 17 | 107 | 9.6 | 17 | -1.1 | 3.6 | 17 | 4.2 | 3.6 | 12 | 112 | 9.7 | 12 | 111 | 10.3 | 12 | -0.5 | 5.4 | 12 |
| DiaBP (mm Hg) | 68 | 5.6 | 17 | 67 | 6.0 | 17 | -0.7 | 5.1 | 17 | 0.4 | 4.7 | 17 | 68 | 7.0 | 17 | 67 | 5.9 | 17 | -0.8 | 3.2 | 17 | 4.7 | 2.6 | 12 | 72 | 5.2 | 12 | 70 | 4.0 | 12 | -1.8 | 3.5 | 12 |
| TC (mg/dL) | 164 | 23.9 | 17 | 146 | 23.1 | 17 | -17.5 | 13.6 | 17 | 19.9 | 16.3 | 17 | 166 | 26.5 | 17 | 150 | 23.7 | 17 | -15.7 | 15.6 | 17 | 21.4 | 13.1 | 12 | 168 | 20.7 | 12 | 155 | 22.5 | 12 | -12.7 | 9.7 | 12 |
| TAG (mg/dL) | 83 | 32.5 | 17 | 72 | 20.3 | 17 | -11.1 | 24.5 | 17 | 10.6 | 22.4 | 17 | 83 | 35.0 | 17 | 66 | 19.9 | 17 | -16.3 | 21.1 | 17 | 15.7 | 28.0 | 12 | 79 | 36.6 | 12 | 70 | 32.9 | 12 | -9.3 | 15.3 | 12 |
| HDLC (mg/dL) | 53 | 9.2 | 17 | 49 | 9.7 | 17 | -3.4 | 5.7 | 17 | 3.5 | 4.8 | 17 | 53 | 10.2 | 17 | 51 | 9.4 | 17 | -1.2 | 4.3 | 17 | 2.3 | 6.5 | 12 | 52 | 8.5 | 12 | 49 | 8.6 | 12 | -2.4 | 5.4 | 12 |
| LDLC (mg/dL) | 94 | 23.8 | 17 | 82.4 | 20.0 | 17 | -11.90 | 9.6 | 17 | 14.3 | 13.8 | 17 | 96.7 | 23.6 | 17 | 85.4 | 20.4 | 17 | -11.3 | 14.0 | 17 | 16.0 | 11.1 | 12 | 100.3 | 24.1 | 12 | 91.8 | 23.1 | 12 | -8.5 | 10.1 | 12 |
| TC/HDLC | 3.2 | 0.7 | 17 | 3.1 | 0.7 | 17 | -0.1 | 0.3 | 17 | 0.2 | 0.4 | 17 | 3.3 | 0.8 | 17 | 3.0 | 0.7 | 17 | -0.3 | 0.3 | 17 | -0.02 | 0.3 | 12 | 3.4 | 0.9 | 12 | 3.3 | 0.8 | 12 | -0.1 | 0.4 | 12 |
| Glucose (mg/dL) | 86.2 | 5.6 | 17 | 84.0 | 4.9 | 17 | -2.2 | 4.5 | 17 | 1.3 | 6.0 | 17 | 85.3 | 7.3 | 17 | 86.4 | 7.2 | 17 | 1.1 | 8.4 | 17 | 3.8 | 6.7 | 12 | 87.8 | 5.7 | 12 | 87.6 | 3.3 | 12 | -0.1 | 5.9 | 12 |
| BUN (mg/dL) | 13.1 | 2.8 | 17 | 12.9 | 2.8 | 17 | -0.2 | 2.1 | 17 | 0.5 | 2.2 | 17 | 13.4 | 2.7 | 17 | 14.0 | 2.3 | 17 | 0.7 | 2.7 | 17 | -0.8 | 2.3 | 12 | 13.5 | 2.6 | 12 | 13.8 | 1.7 | 12 | 0.2 | 2.0 | 12 |
| Creatinine (mg/dL) | 0.9 | 0.1 | 17 | 0.9 | 0.1 | 17 | 0.01 | 0.06 | 17 | -0.04 | 0.07 | 17 | 0.9 | 0.1 | 17 | 0.9 | 0.1 | 17 | 0.01 | 0.05 | 17 | -0.003 | 0.06 | 12 | 0.9 | 0.1 | 12 | 0.9 | 0.1 | 12 | 0.02 | 0.06 | 12 |

Abbreviations: HDP, healthy dietary pattern; WHC, waist-to-hip circumference; SAD, sagittal abdominal diameter; SysBP, systolic blood pressure; DiaBP, diastolic blood pressure; TC, total cholesterol; TAG, triglycerides; HDLC, high-density lipoprotein-cholesterol; LDLC, low-density lipoprotein cholesterol; BUN, blood urea nitrogen.

Supplemental Table S11. Rho values of significant correlations between changes in blood lipids and bacterial taxa.

| TC | HDPs | HDP 1 | HDP 2 | HDP 3 | Washouts | Washout 1 | Washout 2 |
|---|---|---|---|---|---|---|---|
| OTU0039_Alistipes | n.s. | -0.624 | -0.563 | n.s. | n.s. | n.s. | n.s. |
| OTU0056_Roseburia | n.s. | n.s. | n.s. | n.s. | n.s. | 0.505 | n.s. |
| OTU0037_Lachnospiraceae_unclassified | n.s. | n.s. | n.s. | n.s. | n.s. | n.s. | -0.710 |
| OTU0057_Dorea | n.s. | n.s. | n.s. | n.s. | n.s. | n.s. | -0.624 |
| Romboutsia | n.s. | n.s. | n.s. | n.s. | n.s. | -0.498 | n.s. |
| Bacteroidetes_unclassified | n.s. | n.s. | n.s. | n.s. | n.s. | -0.576 | n.s. |
| Dorea | n.s. | n.s. | n.s. | n.s. | n.s. | n.s. | -0.638 |
| Triglycerides | HDPs | HDP 1 | HDP 2 | HDP 3 | Washouts | Washout 1 | Washout 2 |
| OTU0025_Collinsella | -0.540 | n.s. | n.s. | n.s. | n.s. | -0.548 | n.s. |
| OTU0037_Lachnospiraceae_unclassified | -0.580 | -0.802 | n.s. | n.s. | n.s. | n.s. | n.s. |
| OTU0066_Roseburia | -0.550 | n.s. | n.s. | n.s. | n.s. | n.s. | n.s. |
| OTU0043_Ruminococcaceae_unclassified | n.s. | n.s. | n.s. | n.s. | -0.580 | n.s. | n.s. |
| OTU0026_Mediterraneibacter | n.s. | -0.52 | n.s. | n.s. | n.s. | n.s. | n.s. |
| OTU0017_Dorea | n.s. | n.s. | -0.608 | n.s. | n.s. | n.s. | n.s. |
| OTU0032_Mediterraneibacter | n.s. | n.s. | -0.639 | n.s. | n.s. | n.s. | n.s. |
| OTU0038_Faecalibacillus | n.s. | n.s. | -0.529 | n.s. | n.s. | n.s. | n.s. |
| OTU0057_Dorea | n.s. | n.s. | -0.582 | n.s. | n.s. | n.s. | n.s. |
| OTU0076_Lachnospira | n.s. | n.s. | 0.659 | n.s. | n.s. | n.s. | n.s. |
| OTU0071_Ruminococcaceae_unclassified | n.s. | n.s. | n.s. | n.s. | n.s. | -0.496 | n.s. |
| Parabacteroides | n.s. | n.s. | n.s. | n.s. | 0.490 | n.s. | n.s. |
| Akkermansia | n.s. | n.s. | n.s. | n.s. | -0.490 | n.s. | n.s. |
| Dorea | n.s. | n.s. | -0.503 | n.s. | n.s. | n.s. | n.s. |
| Bacteroidetes_unclassified | n.s. | n.s. | -0.692 | n.s. | n.s. | n.s. | n.s. |

Supplemental Table S11. continued

| HDL-C | HDPs | HDP 1 | HDP 2 | HDP 3 | Washouts | Washout 1 | Washout 2 |
|---|---|---|---|---|---|---|---|
| OTU0066_Roseburia | 0.550 | n.s. | n.s. | n.s. | n.s. | n.s. | n.s. |
| OTU0017_Dorea | n.s. | n.s. | 0.555 | n.s. | n.s. | n.s. | n.s. |
| OTU0038_Faecalibacillus | n.s. | n.s. | 0.591 | n.s. | n.s. | n.s. | n.s. |
| OTU0032_Mediterraneibacter | n.s. | n.s. | n.s. | n.s. | n.s. | 0.557 | n.s. |
| OTU0026_Mediterraneibacter | n.s. | n.s. | n.s. | n.s. | n.s. | n.s. | 0.639 |
| Anaerostipes | 0.500 | n.s. | n.s. | n.s. | 0.740 | 0.557 | n.s. |
| Collinsella | 0.550 | n.s. | n.s. | n.s. | 0.640 | 0.561 | n.s. |
| Romboutsia | 0.570 | n.s. | n.s. | n.s. | n.s. | n.s. | 0.708 |
| Akkermansia | 0.480 | n.s. | n.s. | n.s. | n.s. | 0.53 | n.s. |
| Turicibacter | 0.510 | n.s. | n.s. | n.s. | 0.800 | n.s. | 0.703 |
| Blautia | n.s. | n.s. | n.s. | n.s. | 0.630 | 0.656 | n.s. |
| Dorea | n.s. | n.s. | 0.613 | n.s. | 0.690 | 0.574 | n.s. |
| Mediterraneibacter | n.s. | n.s. | n.s. | n.s. | 0.600 | n.s. | 0.726 |
| Ruminococcus | n.s. | n.s. | n.s. | n.s. | 0.510 | n.s. | n.s. |
| Parabacteroides | n.s. | n.s. | -0.544 | n.s. | n.s. | n.s. | n.s. |
| Bacteroidetes_unclassified | n.s. | n.s. | n.s. | -0.767 | n.s. | n.s. | n.s. |
| LDL-C | HDPs | HDP 1 | HDP 2 | HDP 3 | Washouts | Washout 1 | Washout 2 |
| OTU0008_Fusicatenibacter | -0.500 | n.s. | n.s. | n.s. | n.s. | n.s. | n.s. |
| OTU0057_Dorea | n.s. | n.s. | n.s. | n.s. | -0.480 | n.s. | n.s. |
| OTU0039_Alistipes | n.s. | -0.760 | -0.508 | n.s. | n.s. | n.s. | n.s. |
| OTU0035_Romboutsia | n.s. | n.s. | n.s. | n.s. | n.s. | -0.589 | n.s. |
| OTU0056_Roseburia | n.s. | n.s. | n.s. | n.s. | n.s. | 0.501 | n.s. |
| OTU0006_Blautia | n.s. | n.s. | n.s. | n.s. | n.s. | n.s. | -0.691 |
| OTU0026_Mediterraneibacter | n.s. | n.s. | n.s. | n.s. | n.s. | n.s. | -0.764 |
| Clostridiales_unclassified | n.s. | -0.506 | n.s. | n.s. | n.s. | -0.490 | n.s. |
| Romboutsia | n.s. | n.s. | n.s. | n.s. | n.s. | -0.566 | n.s. |
| Blautia | n.s. | n.s. | n.s. | n.s. | n.s. | n.s. | -0.709 |
| Dorea | n.s. | n.s. | n.s. | n.s. | n.s. | n.s. | -0.745 |
| Mediterraneibacter | n.s. | n.s. | n.s. | n.s. | n.s. | n.s. | -0.891 |
| Turicibacter | n.s. | n.s. | n.s. | n.s. | n.s. | n.s. | -0.665 |

Supplemental Table S11. continued

| TC/HDL-C Ratio | HDPs | HDP 1 | HDP 2 | HDP 3 | Washouts | Washout 1 | Washout 2 |
|---|---|---|---|---|---|---|---|
| OTU0057_Dorea | -0.490 | n.s. | n.s. | n.s. | -0.600 | n.s. | -0.645 |
| OTU0056_Roseburia | n.s. | 0.493 | n.s. | n.s. | n.s. | n.s. | n.s. |
| OTU0066_Roseburia | n.s. | -0.572 | n.s. | n.s. | n.s. | n.s. | n.s. |
| OTU0017_Dorea | n.s. | n.s. | -0.624 | n.s. | n.s. | n.s. | n.s. |
| OTU0038_Faecalibacillus | n.s. | n.s. | -0.563 | n.s. | n.s. | n.s. | n.s. |
| OTU0076_Lachnospira | n.s. | n.s. | 0.721 | n.s. | n.s. | n.s. | n.s. |
| OTU0061_Lachnospiraceae_unclassified | n.s. | n.s. | n.s. | 0.668 | n.s. | n.s. | n.s. |
| OTU0025_Collinsella | n.s. | n.s. | n.s. | n.s. | n.s. | -0.529 | n.s. |
| OTU0026_Mediterraneibacter | n.s. | n.s. | n.s. | n.s. | n.s. | n.s. | -0.755 |
| Blautia | -0.580 | n.s. | n.s. | n.s. | -0.650 | n.s. | -0.709 |
| Mediterraneibacter | -0.490 | n.s. | n.s. | n.s. | -0.640 | n.s. | -0.809 |
| Collinsella | -0.540 | n.s. | n.s. | n.s. | -0.730 | -0.588 | -0.745 |
| Bacteroidetes_unclassified | -0.580 | n.s. | -0.626 | 0.698 | n.s. | n.s. | n.s. |
| Clostridiales_unclassified | n.s. | n.s. | n.s. | n.s. | -0.570 | -0.556 | n.s. |
| Dorea | n.s. | n.s. | n.s. | n.s. | -0.690 | n.s. | -0.664 |
| Akkermansia | n.s. | n.s. | n.s. | n.s. | -0.530 | n.s. | n.s. |
| Turicibacter | n.s. | n.s. | n.s. | n.s. | -0.610 | n.s. | -0.756 |
| Ruminococcus | n.s. | n.s. | -0.648 | n.s. | n.s. | n.s. | n.s. |
| Romboutsia | n.s. | n.s. | n.s. | n.s. | n.s. | -0.650 | n.s. |
| Anaerostipes | n.s. | n.s. | n.s. | n.s. | n.s. | n.s. | -0.736 |

Notes: HDP, healthy dietary pattern; TC, total cholesterol; LDL-C, low-density lipoprotein cholesterol; HDL-C, high-density lipoprotein cholesterol; OTU, operational taxonomic unit; n.s., not significant.
